# Assessment of exposure to pesticides: residues in 24 h duplicate diets versus their metabolites in 24 h urine using suspect screening and target analysis

**DOI:** 10.1007/s00216-023-04918-x

**Published:** 2023-09-22

**Authors:** R. Nijssen, A. Lommen, H. van den Top, R. van Dam, C. Meuleman-Bot, M. Tienstra, P. Zomer, S. Sunarto, F. van Tricht, M. Blokland, H. Mol

**Affiliations:** Wageningen Food Safety Research, part of Wageningen University & Research, Akkermaalsbos 2, 6708 WB Wageningen, The Netherlands

**Keywords:** Food, Exposure assessment, Biomonitoring, Non-target measurement, Metabolites

## Abstract

Human biomonitoring can add value to chemical risk assessment by reducing the assumptions regarding consumption rates, residue occurrence, and processing effects and by integrating exposures from different sources (diet, household use, environmental). However, the relationship between exposure and concentration in human matrices is unknown for most pesticides. Therefore, we conducted a pilot study to gain more insight into the qualitative and quantitative relationship between dietary intake of pesticides (external exposure) and urinary excretion (reflecting internal exposure). In this cross-sectional observational study, 35 healthy consumers aged 18–65 years from the region of Wageningen, Netherlands, collected an exact duplicate portion of their diets during 24 h. On the same day, they also collected all their urine. The duplicate diets were analyzed using target screening by GC- and LC-HRMS; each duplicate diet contained at least five, up to 21, pesticide residues. The 24 h urine samples were analyzed using LC-HRMS in a suspect screening workflow. Metabolites were tentatively detected in all 24 h urine samples, ranging from six metabolites corresponding to four pesticides up to 40 metabolites originating from 16 pesticides in a single urine sample. In total, 65 metabolites originating from 28 pesticides were tentatively detected. After prioritization and additional confirmation experiments, 28 metabolites originating from 10 pesticides were identified with confidence level 1 or 2b. Next, quantitative analysis was performed for a selection of pesticides in duplicate diets and their metabolites in 24 h urine to assess quantitative relationships. In the quantitative comparisons between duplicate diet and 24 h urine, it was found that some metabolites were already present in the duplicate diet, which may give an overestimation of exposure to the parent pesticide based on measurement of the metabolites in urine. Additionally, the quantitative comparisons suggest a background exposure through other exposure routes. We conclude that suspect screening of 24 h urine samples can disclose exposure to mixtures of pesticide on the same day in the general population. However, more research is needed to obtain quantitative relationships between dietary intake and exposure.

**Graphical Abstract:**

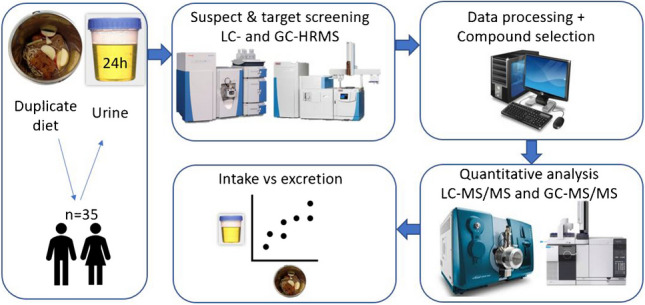

**Supplementary Information:**

The online version contains supplementary material available at 10.1007/s00216-023-04918-x.

## Introduction

External pesticide exposure is typically being assessed using one of the three following approaches. The first approach is through food monitoring data combined with intake estimates, as is for example used in the web-based platform Monte Carlo Risk Assessment (MCRA) [1]. The second approach, slightly closer to actual intake, is through food monitoring data combined with food intake diaries. The third, reflecting the actual intake in 1 day, is the collection and analysis of duplicate diets or total diet studies. Total diet studies (TDS) and duplicate diet analysis are performed using targeted methods to achieve the desired low detection limits. Most of these targeted TDS and duplicate diet studies are focused on a selection of pesticide classes, for example, organophosphates and pyrethroids [2–5]. A drawback of all aforementioned external exposure assessment methods is that only dietary exposure is included, while other sources of exposure may also be important.

An alternative approach to external pesticide exposure assessment is internal exposure assessment using human biomonitoring (HBM). Here the pesticides or their metabolites are measured in human matrices. Urine is the most common matrix used, as it is non-invasive and sufficient amounts are easy to obtain. HBM can provide a more complete exposure assessment, including all routes of exposure. Several HBM studies have been published, focusing on specific pesticides or pesticide classes, for example, pyrethroids [6, 7], organophosphorus pesticides [8], glyphosate [9, 10], or neonicotinoids [11]. HBM of a wider scope of pesticides poses several challenges. The first is that upon intake, many currently used pesticides are rapidly and extenstively metabolized, e.g., by hydroxylation and conjugation, meaning that the target analytes in urine are usually metabolites rather than the parent pesticides. A second challenge is that the human metabolite best reflecting exposure is often not known. Furthermore, analytical reference standards of the metabolites are unavailable in many cases. All this seriously restricts the possibilities for quantitative multi-residue analysis of urine. For this reason, suspect screening methods are the best option for wide-scope HBM studies. Suspect screening involves a generic non-target measurement based on chromatography with high-resolution mass spectrometry, followed by a targeted search using a database with “suspects”, e.g., known and predicted pesticides metabolites [12–14].

Since currently used non-persistent pesticides are rapidly excreted [15–17], a relationship is expected between residues in food consumed on a day and pesticides/metabolites found in 24 h urine. The goal of this study was to investigate such relationship. The primary aim was to assess the qualitative relationship between pesticides/metabolites found in 24-h urine, and pesticides in 24-h duplicate diets. Secondary, for selected pesticides, a quantitative relationship between urinary excretion and dietary intake was investigated, to gain insight in usefulness of urinary data to assess external exposure.

## Materials and methods

### Chemicals and materials

UPLC grade methanol (MeOH), acetonitrile (ACN), ethyl acetate, and water were obtained from Actu-all chemicals (Oss, Netherlands). Formic acid (FA), acetic acid (HAc), sodium acetate, ammonium formate, and ammonium carbonate were obtained from VWR International (Darmstadt, Germany). Magnesium sulfate, dimethyl sulfoxide (DMSO), β-glucoronidase/arylsulfatase from *Helix Pomatia*, dipotassium phosphate, and potassium dihydrogen phosphate were obtained from Sigma-Aldrich (St. Louis, USA). Bondesil PSA was obtained from Agilent Technologies (Santa Clara, USA). Bakerbond C18 was purchased from Avantor Performance Materials (Phillipsburg, USA).

Analytical reference standards and isotope labelled internal standards were purchased from LGC Standards (Teddington, UK), Toronto Research Chemicals (Toronto, Canada), Sigma-Aldrich, HPC Standards (Cunnersdorf, Germany), Bayer CropScience (Monheim am Rhein, Germany), and MerchaChem (Nijmegen, Netherlands). Reference standards of chlorpyrifos-methyl-desmethyl and chlorpyrifos-desethyl were kindly donated by CVUA (Stuttgart, Germany).

Oasis HLB 60 µm/60 mg 96-well plates were purchased from Waters (Milford, USA), Strata-X polymeric reversed phase cartridges (200 mg/6 mL) were purchased from Phenomenex (Utrecht, the Netherlands), and Amicon Ultra 30 kDa centrifugal filter units were purchased from Sigma-Aldrich.

### Study design and sampling

In this study, 35 self-reported healthy consumers participated. The participants (25 female, 10 male) were 19–65 years old, and lived in the Wageningen region in the Netherlands. Ethical approval for this study was obtained from the medical ethical committee of Wageningen University, NL64614.081.18, 20 February 2018.

Sampling was performed in June 2018. Participants collected a duplicate portion of all food and drinks consumed in 1 day in a metal bucket, cooled with dry-ice inside a double-sided wall. For collection of the 24 h urine, the first-morning urine of the food collection day was discarded, all other urine was collected in 24 h urine containers. The first-morning urine of the following day was included in the container. The participants kept a diary of all consumed food and drinks and registered each urine void time and volume.

### Sample pre-treatment

Urine was aliquoted and stored at −80 °C. The average 24 h urine volume was 2.0 L. Duplicate diets were turrax mixed into a slurry, frozen, and lyophilized. The lyophilized powder was stored at −18 °C. The average mass of the unprocessed duplicate diet was 3.32 kg, and the average dry fraction was 14%. Further information on the collected samples is available in the supplementary material. The suspect and target screening analyses were performed in 2018, the confirmatory and quantitative analyses in 2019-mid 2020.

### Target screening duplicate diets

#### Extraction

The duplicate diet was extracted using a QuEChERS method. To 2.5 g lyophilized duplicate diet, 7.5 mL water and 10 mL ACN 1% HAc were added. After shaking for 30 min using a head-over-head shaker, 4 g magnesium sulfate and 1 g sodium acetate were added to induce phase separation. For LC analysis, the ACN extract was diluted 1:1 with water. For GC analysis, an additional dSPE clean-up was performed, 0.5 mL ACN extract and 25 µL PCB-198 (2 µg/mL) were added to a tube containing 50 mg C18 and 150 mg PSA. After centrifugation, the cleaned extract was used for GC analysis.

#### Instrumental analysis

Duplicate diet extracts were measured by LC-HRMS using the method described by Zomer et al. [18]. This method consists of reversed phase chromatography and full scan/vDIA acquisition using a Q-Exactive instrument (Thermo Scientific, Bremen, Germany). Positive and negative ionization modes were measured by separate injections. For GC-HRMS analysis of the duplicate diet extracts, the method described by Mol et al. was performed [19].

#### Data processing

Duplicate diet data from both LC- and GC-HRMS was processed using Tracefinder software version 4.1 (Thermo Scientific). The in-house created target list for the LC data analysis contained precursor ion m/z, retention time, and the m/z of the most intense fragment ion for 212 pesticides. A standard and a spiked sample containing these pesticides were included in the analysis to obtain semi-quantitative results. For GC-HRMS data processing, a target list containing 49 pesticides was created, and a standard containing these 49 pesticides was included in the analysis.

### Suspect screening urine

#### Extraction

Urine samples were extracted using a 96-well plate solid phase extraction method. In this method, 500 µL urine was added to 500 µL of phosphate buffer (pH = 7.4). After equilibration of the SPE material (Oasis HLB 60 µm/60 mg) using MeOH and water, the samples were loaded and washed using 200 µL water. The extracts were eluted using 1 mL of 90% ACN into a well plate, with each well containing 10 µL of DMSO. The extracts were evaporated at 40 °C under a gentle flow of nitrogen and reconstituted in 100 µL 10% MeOH in water containing internal standards 2,4-D-d3, alpha-zearalanol-d4, clenbuterol-d6, salbutamol-d6, and beta-testosteron-d3 at a concentration of 100 ng/mL.

#### Instrumental analysis

Urine suspect screening samples were analyzed using LC-HRMS in full scan mode with a resolution of 140,000 (defined at m/z 200 FWHM). Positive and negative ionization modes were acquired separately. In positive mode eluents consisting of water (A) and MeOH (B), both containing 2 mM ammonium formate and 0.1% FA were used. In negative mode eluents were water (A) and 95% MeOH (B) both containing 10 mM ammonium carbonate. In both modes, the same gradient was used with a flow rate of 0.3 mL/min. The gradient consisted of linear gradient from 0% B to 100% B in 15 min, followed by a 6-min isocratic period at 100% B, return to 0% B in 1 min, and 8 min of equilibration. Dedicated Waters UPLC BEH C18 columns (1.7 µm, 2.1 mm × 100 mm) were used for each ionization mode eluents. The column temperature was 50 °C, autosampler temperature was 10 °C, and the injection volume was 5 µL.

#### Data processing

A suspect list was created in-house for urine suspect screening by aggregating information on pesticide metabolism from Draft Assessment Reports [20] and literature. To create the suspect list, we prioritized 125 pesticides commonly found in food in the Netherlands [21], marketed in high volumes in Dutch agriculture [22], or have dual use as biocides or veterinary drugs. The final suspect list contained the molecular formula of approximately 1700 possible pesticide metabolites (see Supplementary information). Their glucuronide and sulfate conjugates were added resulting in a total of approximately 5000 suspects. As standards for these metabolites are mostly unavailable, only the exact mass of (de)protonated molecules and adducts, and their isotope pattern could be included, no retention time or fragment ion information.

The data processing of the urine suspect screening was performed using MetAlign software suite [23] on a HP Z820 workstation with two Intel® Xeon® E5-2690 CPU 2.90 GHz processors (2 × 8 cores, 2 × 16 virtual) and 64 GB RAM with 64-bit Windows 10 operating system. First, the data was preprocessed for retention time and mass calibration corrections, and data file size was reduced. The mass and retention time corrections were performed based on the isotope labelled internal standards, added after extraction, and endogenous compounds (e.g., endogenous hormones). By including endogenous compounds, this procedure doubles as a control of the sample extraction. The corrected and reduced data was used for automated isotope pattern recognition and adduct and elemental composition analysis. Elemental composition analysis was based on the elements C, H, N, O, P, S, Cl, F, and Br, using a maximum mass error of 1.5 ppm. For Cl and Br, the detection of the characteristic isotope pattern was mandatory. The signal intensity threshold in the preprocessed data was 60,000 in positive mode and 20,000 in negative mode. The data preprocessing resulted in annotations (molecular formula) of all unique retention time-m/z features from the data. Next, suspect screening was performed by matching the molecular formulas of the suspect list with those from the preprocessed and annotated features.

This workflow was later finetuned and used in the HBM4EU Specimen study [12].

### Urine metabolite identification

For metabolite identification, additional experiments were performed. Samples in which phase II metabolites were detected, were subjected to enzymatic deconjugation. The enzyme and procedure chosen was based on previous work [24] and literature [16]. For the conjugates in these references, the deconjugation was shown to be complete. While considered rather generic, this is no guarantee for quantititave conversion of conjugates of other pesticides.

In these deconjugation experiments, 1 mL of urine and 10 µL of β-glucoronidase/arylsulfatase from *Helix pomatia* was used. The sample was deconjugated overnight (16 h) in a water bath at 37 °C. The following morning, the sample was allowed to cool down to room temperature and filtered using an Amicon Ultra 30 kDa centrifugal filter unit. Samples measured without enzymatic deconjugation were filtered using the beforementioned centrifugal filter unit and not subjected to further clean-up.

For instrumental analysis, the urine suspect screening LC methods were used with an adapted HRMS data acquisition method. For metabolite identification, MS^2^ was performed using an inclusion list. The MS^2^ scans were acquired at 35,000 resolution with a stepped collision energy of 30 and 80 normalized collision energy.

Data was reviewed manually using Xcalibur Qualbrowser (Thermo Scientific). In cases where a standard was commercially available, metabolite identity was confirmed with matching m/z, retention time, and fragmentation spectrum to obtain identification at level 1 according to the Schymanski classification [25].

When a standard was not available, the expected fragmentation pattern and retention time, based on the parent compound fragmentation pattern and retention time, were reviewed to explain the molecular structure (level 2b identification). The fragments with the highest intensities were used (up to three fragments); we did not apply an automated strategy for spectrum matching. The metabolite retention time was checked to elute before the parent compound. In case of a conjugated metabolite, both the sample as such and the sample after enzymatic deconjugation were evaluated. The m/z of the conjugated metabolite should be low or not present in the sample after enzymatic deconjugation, while the m/z of the free form should increase after enzymatic deconjugation.

### Quantitative methods

#### Duplicate diets — GC-MS/MS

In duplicate diets, chlorproham, chlorpyrifos, chlorpyrifos-methyl, cyhalothrin-lambda, cypermethrin, deltamethrin, fenvalerate, permethrin, tefluthrin, and transfluthrin were quantified using a GC-MS/MS method. An ethylacetate extraction combined with gel permeation chromatography clean-up was used. More details on extraction, GC and MS settings, and method validation are available in the supplementary material.

#### Duplicate diets metabolites of pyrethroids and chlorpyrifos(/methyl) — LC-MS/MS

In duplicate diets, pyrethroid metabolites (DCCA, DBCA, 3-PBA, 4-F-3-PBA), and metabolites of chlorpyrifos and chlorpyrifos-methyl (TCPy, chlorpyrifos-desethyl, and chlorpyrifos-methyl-desmethyl) were quantified. The samples were extracted using a QuEChERS based method. The extracts were measured on an Sciex Qtrap 6500+ in negative ionization mode. More details on extraction, LC and MS settings, and method validation are available in the supplementary material.

#### Urine method A — chlorpropham metabolite 4-HSA — LC-MS/MS

In method A, chlorpropham metabolite 4-HSA was quantified. This method consisted of addition of internal standard 4-HSA-d7 to the urine sample and ultrafiltration using Amicon Ultra 30kDA filter units. The filtered urine was transferred to an LC vial and injected as such. More details on LC and MS settings and method validation are available in the supplementary material.

#### Urine method B — chlorpyrifos-desethyl and chlopyrrifos-methyl-desmethyl — LC-MS/MS

Method B was used to quantify chlorpyrifos-desethyl and chlorpyrifos-methyl-desmethyl. In this method, the urine sample was diluted 1:1 using ACN 1% FA. Quantification was performed using standard addition at 2 ng/mL and 10 ng/mL. More details on LC and MS settings and method validation are available in the supplementary material.

#### Urine method C — pyrethroid metabolites and TCPy — LC-MS/MS

Method C was used for quantification of pyrethroid metabolites cis-DCCA, trans-DCCA, DBCA, 3-PBA, 4-F-3-PBA, and TCPy (common metabolite of chlorpyrifos and chlorpyrifos-methyl in urine. The samples were deconjugated overnight using β-glucuronidase/arylsulfatase from *Helix pomatia* before SPE clean-up, and were measured in negative ionization mode. More details on extraction, LC and MS settings, and method validation are available in the supplementary material.

## Results and discussion

### Duplicate diets target screening LC-HRMS and GC-HRMS

Duplicate diets were analyzed with LC- and GC-HRMS in a target screening approach. For quality control of the LC-HRMS target screening, the peak area and retention time of all 212 compounds (supplementary material: Duplicate diets LC-HRMS target list) in solvent standards injected before and after the samples were evaluated. The median peak area ratio of compounds in the standard (concentration corresponding to 40 ng/g in duplicate diet) before/after the samples was 1.18, indicating good detection stability. The retention time shifts were between −0.04 min and +0.05 min for 97% of the compounds, showing good chromatographic stability. The screening LODs were not specifically established for the duplicate diet matrix. Based on previous work [18], most pesticides are expected to be detectable reliably (95% confidence) down to 10 µg/kg; however, for certain pesticides, LODs of 50 µg/kg or even higher are expected. Experience has shown that detection is often possible at lower levels, albeit with lower level of confidence. Non-detection in this study is not a full proof of absence, and this was also not the aim of this study.

For quality control in GC-HRMS target screening, the analyte response in standards in wheat extracts injected before and after the samples was evaluated, and the response of the internal standard PCB198 in all samples. The ratio of response in standards in wheat (corresponding to 25 ng/g in extract) measured after and before the samples was between 0.02 and 1.04. This shows that for some compounds, the sensitivity strongly decreased, while for others, the response was stable. The average ratio was 0.65 showing a decline in sensitivity. For most compounds, the detection limit, estimated based on response in standards, was ≤2.5 ng/g. For chlorothalonil, cyphenothrin, fenpropathrin, fipronil, folpet, and tefluthrin, the limit of detection was >25 ng/g. A decline of the internal standard response by 64% was observed, also indicating that sensitivity decreased towards the end of the sample sequence. The response drift was attributed to residual fat in the final QuEChERS extracts. Furthermore, very high abundance of caffeine and theobromine was observed in most samples. The detection of pesticides co-eluting with these compounds may have been affected due to C-trap saturation issues. Despite these challenges, GC-HRMS target screening provided valuable information on pesticide residues present in the diets.

In total, between 5 and 21 pesticide residues were detected in each duplicate diet sample, clearly showing concurrent exposure to mixtures of pesticides during a day. Pirimiphos-methyl (LC-HRMS) was detected in all duplicate diets. Other pesticides with a high detection frequency were chlorpyrifos-methyl (94%, GC-HRMS), deltamethrin (86%, GC-HRMS), chlorpropham (74%, GC-HRMS), and boscalid (71%, LC-HRMS). Most estimated concentrations were below 50 ng/g dry weight; however, levels up to 500 ng/g lyophilized sample were detected for cyprodinil and pyrimethanil. In Table 1, detection frequency and semi-quantitative results are summarized, for compounds detected in >15% of the duplicate diet samples. Further data is available in the supplementary information.

**Table 1:** Semi-quantitative results of LC-HRMS and GC-HRMS target screening, compounds detected in >15% of duplicate diet samples. Estimated concentrations in lyophilized duplicate diet material, median concentration are calculated based on positive samples

Pesticide	*N* pos	% pos	Screening method	Max ng/g	Median ng/g
Pirimiphos-methyl	35	100%	LC	104	17
Chlorpyrifos-methyl	33	94%	GC	12	3.8
Deltamethrin	30	86%	GC	79	22
Chlorpropham	26	74%	GC	82	8.5
Boscalid	25	71%	LC	37	5.3
Trifloxystrobin	23	66%	LC	29	0.3
Tebuconazole	22	63%	LC	9.0	0.9
Carbendazim	15	43%	LC	10	0.7
Pyrimethanil	14	40%	LC	468	39
Tetrahydrophthalimide cis-1,2,3,6-(THPI, metabolite of captan/captafol)	13	37%	GC	5.2	2.1
Cyprodinil	12	34%	LC	509	10
Ortho-phenylphenol	12	34%	GC	15	1.3
Fluopyram*	12	34%	LC	-	-
Fludioxonil	11	31%	LC	252	32
Azoxystrobin	10	29%	LC	15	3.1
Chlorantraniliprole	9	26%	LC	7.8	1.8
Propamocarb	9	26%	LC	151	16
Pyraclostrobin	9	26%	LC	30	6.1
Epoxiconazole	8	23%	LC	2.4	0.4
Iprodion	8	23%	GC	46	11
Acetamiprid	6	17%	LC	20	6.7
Dimethomorph	6	17%	LC	5.9	2.3
Imidacloprid	6	17%	LC	18	3.4
Metalaxyl	6	17%	LC	12	1.6
Thiacloprid	6	17%	LC	6.7	1.9
Bifenthrin	6	17%	GC	868	1.8

*By additional retrospective screening, no semi-quantitative results available

In the EFSA report on pesticides in food sampled in 2018 [26], the top 10 most often quantified pesticides amenable to multi-residue methods in food (mostly fresh fruit and vegetables) were boscalid, imazalil, fluopyram, fludioxonil, acetamiprid, azoxystrobin, pyrimethanil, cyprodinil, pyraclostrobin, and tebuconazole. Eight pesticides from this top 10 were detected in >15% of the duplicate diet samples. Fluopyram was not included in the target screening methods; however, it was retrospectively searched in the duplicate diet datafiles based on m/z, retention time, and fragment ion m/z and was detected in 34% of the samples. Imazalil was detected in 6% of the duplicate diets. The discrepancy for imazalil with the EFSA monitoring data is most probably due to its main use as post-harvest fungicide on citrus, with the residue mostly on the peel which is removed by the consumer.

Pirimiphos-methyl was detected in all duplicate diets, in contrast to the EFSA report in which pirimiphos-methyl was detected in 0.81% of the samples. However, the EFSA report focuses mainly on fruit and vegetables with limited analysis of wheat grain, where pirimiphos-methyl is mainly applied in storage. Wheat is consumed in large volumes in bread and/or pasta, which explains the higher detection frequency in the duplicate diets. This may also explain the high detection rate for chlorpyriphos-methyl, which was also applied to wheat grain during storage in the EU until 2020.

Chlorpropham is mainly used as a sprout inhibitor in potato storage. It was detected in 74% of the duplicate diet samples. The five samples with the highest estimated concentration chlorpropham corresponded to participants that consumed potato products.

The relatively high detection frequency of trifloxystrobin could be caused by consumption of soft fruit (e.g., strawberry). Trifloxystrobin is detected mainly in strawberry, cherry, and grapes in the Netherlands [21]. These types of fruit were in season during the sampling period and were consumed by the participants.

### 24 h Urine suspect screening

Before data preprocessing, the method performance was evaluated through the isotope labelled internal standards in the urine samples. In all 24 h urine samples, all five isotope labelled internal standards were detected. Relative standard deviations of the peak intensity were 26% for salbutamul-d6 (ESI+), 18% for clenbuterol-d6 (ESI+), 9% for beta-testosterone-d3 (ESI+), 9% for 2,4-D-d3 (ESI−), and 14% for alpha-zearalanol-d4 (ESI−), indicating consistent instrument performance.

Next, the data (pre-)processing was done to align retention times, reduce the mass error, and annotate all features (accurate mass to molecular formula) in the full scan data. In data preprocessing, signals of endogenous compounds were used as extraction control of each sample.

The enormous amounts of features is the main challenge in the suspect screening approach. To illustrate this: in one negative mode urine sample datafile, 16,845 unique, adduct grouped accurate m/z–retention time combinations were detected, using a maximum mass error of 1.5 ppm. These unique accurate m/z–retention time features can be solved to 387,678 possible molecular formulas, with the use of the modified seven golden rules [27]. Matching all these possible molecular formulas to the suspect list generated 4024 matches. When requiring detection of at least two isotopes in a predefined intensity range, the number of possible molecular formulas was lowered to 88,944 molecular formulas. The number of matches to the suspect database remained very high with 1183 matches. Moving up to three required isotope peaks, still 8292 molecular formulas are possible. While increasing the number of required isotopes reduces the numbers of molecular formulas (and suspect matches), this also reduces the detectability, as for substances with low MS sensitivities, the lower abundant isotopes will not be detected. As urinary pesticide metabolites are anticipated to be present at low signal intensities, it is clear that alternative filtering is needed to select relevant matches for further annotation and identification.

Since many pesticides contain halogens, and organophosphorus pesticides a PO3 moiety, it was decided to filter on these to start with. Filtering on formulas containing Cl, Br, F, and PO3 lowered the number of matches to the database in this particular example file to 39. Obviously, suspects lacking Cl, Br, F, or PO3 will not be detected at this stage. Especially for pesticides/metabolites only consisting of CHNO, without further a priori information, there are too many tentative matches for follow up for identification. Table 2 shows the number of unique accurate m/z and molecular formulas for one representative negative mode urine datafile, and the effect of the above described filtering on the matches.

**Table 2:** Overview of number of unique m/z in a negative data file for one representative urine sample. All molecular formulas are derived from accurate mass (singly charged, +-1.5ppm mass error), using the modified seven golden rules

Filter:	None	2 isotopes	3 isotopes	Cl	Br	F	PO3
	Number of detects and suspect screening matches						
#unique accurate m/z	13,870						
#unique accurate m/z + RT	16,845						
#possible mol.forms	387,678	88,944	8292	650	31	303,818	13,091
min intensity	35,748	35,748	51,121	40,190	51,729	35,748	35,748
matches with database	4024	1183	292	12	0	26	1
min intensity of database matched signal	50,059	68,035	458,588	120,615	0	53,817	143,701

In this study, an additional filtering strategy was possible by making use of the data obtained from the duplicate diets. Urine samples corresponding to diets with high levels of a certain pesticide were compared to urine samples corresponding to diets where the selected pesticide was not detected. In these urine samples, searches were performed specifically for metabolites of these pesticides. This approach led to the tentative detection of 25 additional pesticide metabolites, including CHNO compounds originating from pyrimethanil, cyprodinil, and propamocarb. In several cases, multiple metabolites of the same pesticide were found in a urine sample, providing additional support for these detects. Using the now known retention time for each of the tentatively detected metabolites, all urine samples were re-searched in a targeted way based on exact mass and retention time. This enabled detection in other urine samples with lower signal intensities.

In total, 65 metabolites corresponding to 28 pesticides were tentatively detected in the 24 h urine samples. Metabolites were tentatively detected in all 24 h urine samples, ranging from six metabolites corresponding to four pesticides up to 40 metabolites originating from 16 pesticides in a single urine sample. All results of the urine suspect screening are summarized in Table 3.

**Table 3:** Overview of tentatively detected metabolites in 24 h urine samples. In some cases, multiple isomeric metabolites were detected and some metabolites were detected in both ionization modes

Parent pesticide	Metabolite name or composition change	Metabolite molecular formula	ESI	detection frequency
2,4-D	2,4-D	C8H6Cl2O3	Neg	3%
Acetamiprid/chlorpropam	Acetamiprid-C4H5N3+O+SO3 chlorpropham-C4H6O+SO3	C6H6ClNO4S	Neg	80%
Acetamiprid	Acetamiprid-N-desmethyl	C9H9ClN4	Neg	83%
Boscalid	Boscalid-OH+C6H8O6	C22H20Cl2N2O8	Neg	17%
Boscalid	Boscalid-OH+SO3	C18H12Cl2N2O5S	Neg	63%
Chlorpropham	4-Hydroxychlorpropham+C6H8O6	C16H20ClNO9	Neg	26%
Chlorpropham	4-HSA (hydroxychlorpropham+SO3)	C10H12ClNO6S	Neg	66%
Chlorpyrifos/chlorpyrifos-methyl	TCPy+C6H8O6	C11H10Cl3NO7	Neg	40%
Chlorpyrifos/chlorpyrifos-methyl	Chlorpyrifos-C4H9O2PS fragment	C5H2NOCl3	Neg	54%
Chlorpyrifos/chlorpyrifos-methyl	3,5,6-Trichloro-2-pyridinol (TCPy)	C5H2Cl3NO	Pos	66%
Chlorpyrifos-methyl	Chlorpyrifos-methyl-desmethyl	C6H5Cl3NO3PS	Neg	83%
Cyfluthrin/cypermethrin/permethrin/transfluthrin	cyfluthrin-C14H8FNO+C6H8O6 cypermethrin-C14H9NO+C6H8O6 permethrin-C13H10O+C6H8O6 transfluthrin-C7H2F4+C6H8O6	C14H18Cl2O8	Neg	14%
Cyprodinil	+O+C6H8O6 (isomer 1)	C20H23N3O7	Pos	11%
Cyprodinil	+O+C6H8O6 (isomer 2)	C20H23N3O7	Pos	11%
Cyprodinil	+O+C6H8O6 (isomer 3)	C20H23N3O7	Pos	23%
Cyprodinil	+O+SO3 (isomer 1)	C14H15N3O4S	Neg	29%
Cyprodinil	+O+SO3 (isomer 2)	C14H15N3O4S	Neg	54%
Cyprodinil	+O+SO3	C14H15N3O4S	Pos	29%
Cyprodinil	+O2	C14H15N3O2	Pos	14%
Cyprodinil	+O2+C6H8O6	C20H23N3O8	Pos	9%
Cyprodinil	+O2+SO3 (isomer 1)	C14H15N3O5S	Neg	17%
Cyprodinil	+O2+SO3 (isomer 2)	C14H15N3O5S	Neg	23%
Cyprodinil	+O3+SO3	C14H15N3O6S	Neg	31%
Dimethomorph	-CH2+SO3	C20H20ClNO7S	Neg	3%
Dimethomorph	-CH2+C6H8O6	C26H28ClNO10	Neg	3%
Fenhexamid	+O+SO3	C14H17Cl2NO6S	Neg	17%
Fludioxonil	+O+C6H8O6 (NH3-adduct)	C18H14N2O9F2	Pos	20%
Fludioxonil	+O+C6H8O6	C18H14N2O9F2	Neg	37%
Imazalil	+H2O2+C6H8O6	C20H24N2O9Cl2	Pos	3%
Imidacloprid	Imidacloprid 5-hydroxy	C9H10Cl1N5O3	Pos	14%
Imidacloprid	Imidacloprid 5-hydroxy	C9H10Cl1N5O3	Neg	20%
Imidacloprid	Imidacloprid olefin	C9H8Cl1N5O2	Pos	29%
Imidacloprid	-NO2+H	C9H11ClN4	Pos	31%
Iprodione	Iprodione metabolite M650F06	C10H7Cl2N3O3	Neg	46%
Linuron	Linuron (Na-adduct)	C9H10N2O2Cl2	Pos	3%
MCPA	+O	C9H9O4Cl	Neg	6%
Paraclox	paraclox	C8H6ClNO3	Neg	9%
Pencycuron	+O	C19H21N2O2Cl	Pos	11%
Pencycuron	+O	C19H21N2O2Cl	Neg	14%
Pencycuron	chlorohippuric acid (pencycuron-C10H13N+O2)	C9H8ClNO3	Neg	60%
Pirimiphos-methyl	-C3H6	C8H14N3O3PS	Neg	20%
Pirimiphos-methyl	Pirimiphos-methyl-desmethyl	C10H18N3O3PS	Neg	66%
Propamocarb	+O	C9H20N2O3	Pos	63%
Propamocarb	Propamocarb	C9H20N2O2	Pos	71%
Propyzamide	+H2O3	C12H13N1O4Cl2	Neg	3%
Propyzamide/dichlormate	Dichlorohippuric acid	C9H7Cl2NO3	Neg	23%
Pyraclostrobin	-CH2O+O+SO3	C18H16ClN3SO7	Neg	3%
Pyrimethanil	+O	C12H13N3O	Pos	9%
Pyrimethanil	+O+C6H8O6	C18H21N3O7	Neg	11%
Pyrimethanil	+O+SO3 (isomer 1)	C12H13N3SO4	Neg	40%
Pyrimethanil	+O+SO3 (isomer 2)	C12H13N3SO4	Neg	71%
Pyrimethanil	+O+SO3	C12H13N3SO4	Pos	57%
Pyrimethanil	+O2+SO3	C12H13N3SO5	Pos	20%
Pyrimethanil	+O2+SO3 (isomer 1)	C12H13N3SO5	Neg	29%
Pyrimethanil	+O2+SO3 (isomer 2)	C12H13N3SO5	Neg	34%
Pyrimethanil	+O2+SO3 (isomer 3)	C12H13N3SO5	Neg	43%
Spirodiclofen	-C6H10O	C15H14Cl2O3	Neg	9%
Sulcotrione	-C6H6O	C8H7O4SCl	Neg	46%
Tebuconazole	-H2+O2	C16H20ClN3O3	Pos	6%
Tebuconazole	+O2+C6H8O6	C22H30ClN3O9	Neg	6%
Tebuconazole	-H2+O2+C6H8O6	C22H28ClN3O9	Neg	6%
Tebuconazole	Hydroxytebuconazole+C6H8O6	C22H30ClN3O8	Neg	14%
Tebuconazole	-H2+O2	C16H20ClN3O3	Neg	17%
Thiamethoxam/clothianidin	Thiamethoxam-C2H2O clothianidin	C8H10ClN5O3S	Neg	40%
Thiametoxam	-NO2+H	C8H11ClN4OS	Pos	6%

Our data shows high detection frequencies for acetamiprid-N-desmethyl (83%), chlorpyrifos-methyl-desmethyl (83%), 4-HSA (66%), and pirimiphos-methyl-desmethyl (66%) in 24 h urine samples. In the HBM4EU specimen study, comparable detection frequencies were found for acetamiprid-N-desmethyl (87%); however, the detection frequency in the specimen study was lower for chlorpyrifos-methyl-desmethyl (15%), 4-HSA (44%), and pirimiphos-methyl-desmethyl (36%) [12]. Allthough the detection frequencies were lower, 4-HSA and pirimiphos-methyl compounds were among the four most frequently detected compounds in the specimen study. The difference in detection frequency of chlorpyrifos-methyl-desmethyl could be explained by the ban on chlorpyrifos-methyl use in Europe since 2020, which was after the samples of this study were collected, but before most of the samples in HBM4EU Specimen study were collected.

### Metabolite identification

A selection of the metabolites detected in the suspect screening method was subjected to further identification by acquiring LC-HRMS^2^ spectra. The sample with the highest signal for the selected metabolite was used in these experiments. However, for some metabolites, the signal intensity was still too low to acquire a suitable MS^2^ spectrum for compound identification.

In total, 28 metabolites were identified at level 1 or 2 on the confidence scale of Schymanski et al. [25]. Six of these 28 metabolites were identified in both positive and negative ionization modes. Level 1 identification is a full identification against a reference standard. Level 2b corresponds to a “probable structure” based on diagnostic experimental evidence. Here this included diagnostic MS/MS fragments and ionization behavior as expected based on the parent compound (as far as the metabolite was structurally similar), retention time information (metabolites being more polar and eluting before the parent compound), results from the deconjugation experiments, and co-occurrence of other metabolites from the same parent pesticide in the same urine sample. All identified metabolites at level 1 and 2b are summarized in Table 4.

**Table 4:** Metabolites with identification level

Metabolite	Molecular formula	Mode	RT	Identification level
Acetamiprid-N-desmethyl	C9H9N4Cl	ES+	8.14	1
Acetamiprid-N-desmethyl	C9H9N4Cl	ES−	8.45	1
Boscalid-OH*	C18H12O2N2Cl2	ES+	11.43	1
Boscalid-OH*	C18H12O2N2Cl2	ES−	11.46	1
Boscalid-OH-GlcA	C24H20O8N2Cl2	ES−	8.78	2b
Boscalid-OH-SO3	C18H12N2Cl2O5S	ES−	9.70	2b
Chlorpropham-OH*	C10H12ClNO3	ES+	10.92	1
Chlorpropham-OH*	C10H12ClNO3	ES−	10.73	1
Chlorpropham-OH-SO3 (4-HSA)	C10H12O6NClS	ES−	9.00	1
Chlorpropham-OH-GlcA	C16H20ClNO9	ES−	8.02	2b
Chlorpyrifos: TCPy	C5H2Cl3NO	ES−	9.73	1
Chlorpyrifos-methyl-desmethyl	C6H5NO3PSCl3	ES−	10.20	1
Cyprodinil-di-OH-mono-SO3	C14H14O5N3S	ES−	10.17	2b
Cyprodinil-OH*	C14H15ON3	ES+	9.35	1
Cyprodinil-OH*	C14H15ON3	ES−	12.49	1
Cyprodinil-OH-GlcA	C20H23O7N3	ES+	8.22	2b
Cyprodinil-OH-SO3	C14H14O4N3S	ES−	10.57	2b
Cyprodinil-tri-OH*	C14H15O3N3	ES+	5.47	2b
Cyprodinil-tri-OH-mono-SO3	C14H15O6N3S	ES−	8.36	2b
Imidacloprid olefin (-H2)	C9H8ClN5O2	ES+	7.14	1
Imidacloprid olefin (-H2)	C9H8ClN5O2	ES−	6.67	1
Imidacloprid, 5-hydroxy	C9H10O3N5Cl	ES+	7.30	1
Imidacloprid, 5-hydroxy	C9H10O3N5Cl	ES−	7.41	1
Imidacloprid, desnitro	C9H11N4Cl	ES+	5.60	1
Pirimiphos-methyl-N-desmethyl	C10H18O3N3PS	ES−	10.22	2b
Propamocarb	C9H20O2N2	ES+	5.38	1
Propamocarb-N-oxide	C9H20O3N2	ES+	5.95	1
Pyrimethanil-di-OH-mono-SO3	C12H13O5N3S	ES−	8.23	2b
Pyrimethanil-OH (M605F002)	C12H13N3O	ES+	7.56	1
Pyrimethanil-OH (M605F002)	C12H13N3O	ES−	10.39	1
Pyrimethanil-OH-GlcA	C18H21N3O7	ES+	6.80	2b
Pyrimethanil-OH-SO3	C12H13O4N3S	ES−	8.68	2b
Tebuconazole-OH*	C16H22ClN3O2	ES+	13.30	1
Tebuconazole-OH-GlcA	C22H30ClN3O8	ES+	12.51	2b

*Detected after enzymatic deconjugation

### Qualitative comparison duplicate diet vs urine

In order to qualitatively compare the detection of pesticide/metabolites in urine with the pesticide residues found in the duplicate diets, the overall occurrence in the diet was plotted against that in urine. For urine, the parent pesticides related to the metabolites were counted, i.e., in case of detection of multiple metabolites for the same pesticide, this is counted as one in urine.

**Fig. 1:**
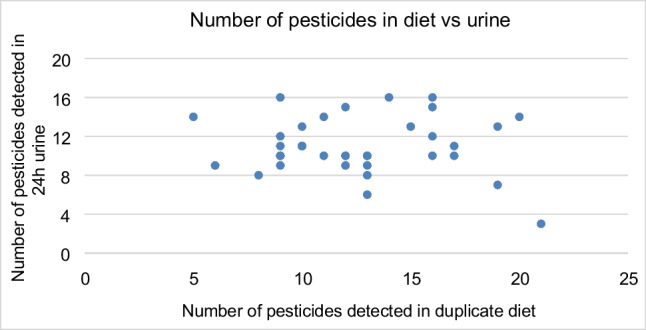
Qualitative comparison of number of pesticides detected in duplicate diet vs 24 h urine

In Fig. 1, the number of detected pesticides in a duplicate diet (target screening by LC-HRMS and GC-HRMS) compared to the number of pesticides for which metabolites were detected in the corresponding 24 h urine (LC-HRMS suspect screening) is shown. There was no clear qualitative correlation in number of pesticides between intake and exposure.

However, overall for most pesticides, the detection frequencies in duplicate diet and 24 h urines were similar. For example, boscalid was detected in 71% of duplicate diets, and one or more boscalid metabolites were detected in 66% of 24 h urine samples, and fludioxonil was detected in 31% of duplicate diets and in 37% of 24 h urines. In contrast, for some other pesticides, differences in detection rates in duplicate diet and 24 h urine were found. Acetamiprid was detected in 17% of the duplicate diets, while one or more metabolites of acetamiprid were present in 97% of 24 h urines. A similar result was found for imidacloprid with 17% detection frequency in diets and 46% detection frequency of one or more metabolites of imidacloprid in 24 h urine. For imidacloprid, a possible explanation could be additional exposure through household uses, for example, flea treatments for pets. Another cause for higher detection frequency in urine might be that complete excretion of a pesticide is not reached after 24 h, which means some of the metabolites in urine could originate from food consumed in the days prior to the duplicate diet collection. An example where the detection frequency in duplicate diets was higher than in 24 h urine was tebuconazole, with 63% detection frequency in duplicate diets and 26% detection frequency of one or more tebuconazole metabolites in 24 h urine. Lower detection frequencies in urine might be caused by lower detectability in urine due to incomplete uptake, urinary excretion of the parent pesticide as multiple metabolites, and lower sensitivity of the metabolites compared to the parent compound.

### Quantitative analysis

For selected pesticides (chlorpropham, pyrethroids, chlorpyrifos and chlorpyrifos-methyl), quantitative analyses were performed in both duplicate diets and urine to obtain accurate concentrations for quantitative comparisons. Below, for each of the pesticides, first the quantitative results in each matrix are described and compared to available existing data. Then investigations into quantitative relationships diet/urine are discussed. The full results of all quantitative methods are included in the supplementary material.

#### Duplicate diets

Chlorpropham was quantitatively determined by GC-MS/MS. It was found in 100% of the duplicate diets in a concentration ranging from 0.5 to 92.6 ng/g. The highest total intake of chlorpropham was 0.58 µg/kg bw per day which was well below the acceptable daily intake (ADI) of 50 µg/kg bw per day [28]. In the GC-HRMS suspect screening method, chlorpropham was detected in 74% in duplicate diet samples. The higher detection frequency in the quantitative GC-MS/MS method is caused by a lower detection limit. In a French total diet study, chlorpropham was detected in almost 10% of all measured foods, while it was detected in 87.5% of the subgroup potatoes and potato products [29]. In our study, 45% of the participants consumed potato products, which indicates that consumption of potato products on the collection day does not explain the complete dietary intake of chlorpropam.

Pyrethroids: Cypermethrin was detected in 100% of duplicate diets (0.79–11.8 ng/g). For cypermethrin, the highest intake corresponded to 0.11 µg/kg bw per day, below the ADI for cypermethrin of 5 µg/kg bw per day [30]. Deltamethrin was detected in 74% of duplicate diets (1.21–13.6 ng/g). The maximum intake of deltamethrin was 0.12 µg/kg bw per day, below the ADI of 10 µg/kg bw per day [31]. Permethrin was detected in 91% of duplicate diets (0.23–1.38 ng/g), with a maximum intake of 0.01 µg/kg bw per day, below the ADI of 50 µg/kg bw per day. Cyhalothrin lambda, fenvalerate, tefluthrin, and transfluthrin were not detected in the duplicate diets. The detection frequencies of cypermethrin and deltamethrin in this study are higher than those presented by Morgan et al. [5], where cypermethrin was detected in 7% and deltamethrin in 17% of duplicate diet samples; however, Morgan et al. used higher detection limits compared to our study, since the foods in the Morgan study were not lyophilized. The maximum concentrations found by Morgan et al. were much higher, 154 ng/g and 16.3 ng/g in non-lyophilized foods for cypermethrin and deltamethrin, respectively. In the duplicate diet study by Melnyk et al., 6 out of 9 (67%) duplicate diets contained cypermethrin and 1 sample (11%) contained deltamethrin [4]. In both the Morgan and Melnyk studies, the samples were taken in the USA. There is no recent data on pyrethroids in duplicate diets in Europe. However, both cypermethrin and deltamethrin are relatively frequently found in raw agricultural commomdities [32].

Low levels of the pyrethroid metabolites 3-PBA (0.20–0.74 ng/g) and DCCA (0.27–0.75 ng/g) were detected in 54% and 23% duplicate diets. DBCA and 4-F-3-PBA were not detected in the duplicate diets.

Chlorpyrifos-methyl was detected in all duplicate diets (0.69–13.2 ng/g) in the GC-MS/MS method. The maximum intake of chlorpyrifos-methyl was 0.08 µg/kg bw per day. Chlorpyrifos was also detected in all duplicate diets. The concentration range of chlorpyrifos in duplicate diets was 0.13 to 1.43 ng/g. The maximum intake of chlorpyrifos was 0.01 µg/kg bw per day. In 2019, EFSA concluded that for chlorpyrifos and chlorpyrifos-methyl, no safe exposure levels (e.g., ADI) can be set, as the genotoxic potential of chlorpyrifos and chlorpyrifos-methyl is inconclusive [33]. However, the ADI established in 2015 was 10 µg/kg bw per day [20].

We performed additional measurements of chlorpyrifos-methyl metabolites in duplicate diets because, as described by Hernandez et al. [34], chlorpyrifos-methyl transformation products that are also human metabolites can be present in food products. Both TCPy and chlorpyrifos-methyl-desmethyl were detected in all duplicate diet samples, in concentrations ranging from 0.7 to 19.6 ng/g for TCPy and 0.14 to 34.4 ng/g for chlorpyrifos-methyl-desmethyl. Chlorpyrifos-desethyl was not detected in duplicate diet samples.

### 24 h Urine

For chlorpropham, several metabolites were found in urine (see Table 4). From a previous study [24], it was known that 4-HSA is the best detectable human urinary metabolite; therefore, the quantitative urine analysis was limited to this. In the quantitative LC-MS/MS method A, 4-HSA was detected in 69% of the urine samples in a concentration range of 0.1–89.6 ng/mL. The detection frequency corresponds with the detection frequency of 66% in the suspect screening method. In the Dutch pesticides and residents study, 4-HSA was analyzed in 309 urine samples with a detection frequency of 81%, in a concentration range of 0.1–175 ng/mL [24].

In humans, most pyrethroids are metabolized by hydrolysis of the molecule into a phenoxybenzoic acid moiety and a carboxylic acid moiety, which are then (partially) conjugated. The quantitative analysis method included an enzymatic deconjugation, hence the phase I metabolites were the target compounds measured. It should be noted that these metabolites are not specific, with the exeption of DBCA (deltamethrin). DCCA is a common metabolite for cypermethrin, cyfluthrin, permethrin, and transfluthrin. 3-PBA is a common metabolite for many pyrethroids. 4-F-3-PBA is a metabolite for cyfluthrin and flumethrin.

The deltamethrin metabolite DBCA was detected in 94% of 24 h urine samples using LC-MS/MS, in the concentration range 0.06–3.12 ng/mL. The detection frequency of DBCA found by Rodzaj et al. in Polish men was 32% and the maximum detected concentration was 8.26 ng/mL [6]. The HBM guidance value for DBCA is 130 ng/mL [35]. The HBM guidance value represents the estimated concentration at which the ADI is not exceeded, and therefore may be considered safe.

DCCA (sum of cis and trans isomers) was detected in all 24 h urine samples using LC-MS/MS method C, in the concentration range 0.05–1.59 ng/mL. These concentrations are below the HBM guidance value for DCCA, which is 45 ng/mL [35]. The detection frequency of DCCA in the quantitative method aligns with the 99.7% detection frequency reported by Li et al. in children in New Zealand [36]. Rodzaj et al. reported detection frequencies of 36% for cis-DCCA and 76% for trans-DCCA in Polish men [6].

Additionally, the common pyrethroid metabolite 3-PBA was detected in all 24 h urine samples (0.05–1.21 ng/mL), whereas the cyfluthrin/flumethrin metabolite 4-F-3-PBA was not detected in any of the 24 h urine samples. The detection frequency of 3-PBA is commonly high, it was quantified in nearly all urine samples in the Flemish Environment and Health study 2016–2020 [37] and also in children from New Zealand, the detection frequency was nearly 100% [36]. The concentrations found for 3-PBA in this study are far below the HBM guidance values of 9.6 to 33 ng/mL [35].

Chlorpyrifos and chlorpyrifos-methyl undergo metabolic activation to their oxon-form and are then hydrolized to form TCPy. Other known metabolites include chlorpyrifos-desethyl and chlorpyrifos-methyl-desmethyl. All these metabolites are not necessarily human metabolites, they may already be present in food as plant or processing metabolites. TCPy (0.55–10.2 ng/mL) and chlorpyrifos-methyl-desmethyl (0.12–8.34 ng/mL) were detected in 100% and 97% of the 24 h urine samples. Chlorpyrifos-desethyl was not detected in 24 h urine samples. The detection frequency of TCPy aligns with the results of the Flemish Environment and Health study 2016–2020 where TCPy was quantified in all samples [37] and the study of Morgan et al. in American preschool children where TCPy was quantified in 99% of the urine samples [38].

### Quantitative comparison duplicate diets vs urine

For chlorpropham (4-HSA), cypermethrin+permethrin (DCCA), deltamethrin (DBCA), and chlorpryrifos/chlorpyrifos-methyl (TCPy, chlorpyrifos-methyl-desmethyl), quantitative relationships between duplicate diet concentration and 24 h urine concentration were investigated. To assess these relationships, the total intake and excretion were calculated. The urinary metabolites were expressed as the parent compound. Only the sample pairs in which the pesticide/metabolite was detected in both diet and urine were used for the quantitative comparison.

**Fig. 2:**
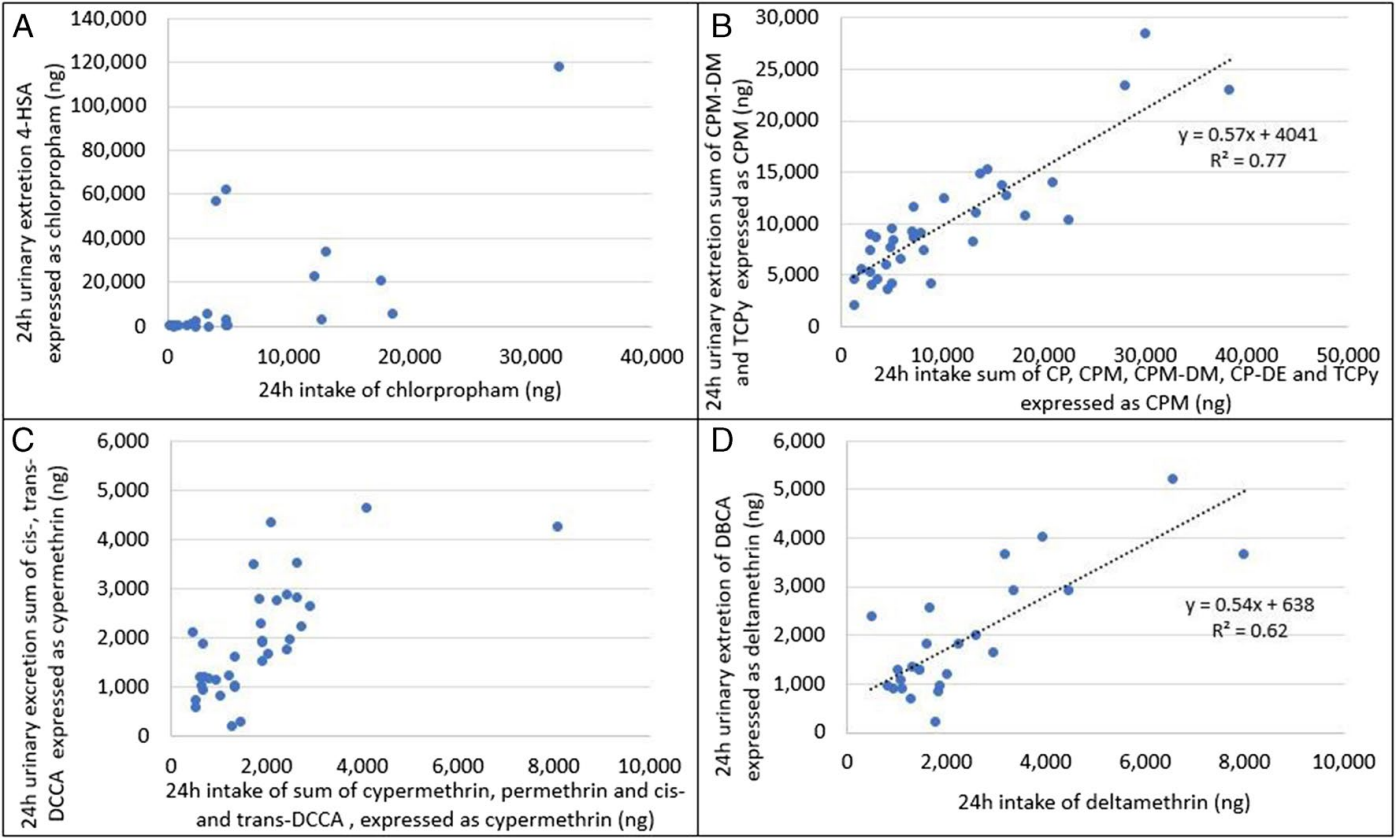
Quantitative relation between excretion and intake for chlorpropham (**A**), chlorpyrifos+chlorpyrifos-methyl (**B**), cypermethrin+permethrin (**C**), and deltamethrin (**D**)

The quantitative comparison for chlorpropham and its metabolite 4-HSA, expressed as chorpropham, is shown in panel A in Fig. 2. In 12 of the 24 sample pairs included in this analysis, the excretion is higher than the dietary intake. Alternative exposure routes are unlikely for chlorpropham, as it is not registered for household use or as veterinary drug. As chlorpropham is mainly present in potatoes, which are usually consumed at dinner in the Dutch diet, it is possible that chlorpropham intake from the day before duplicate diet collection is detected in the urine, and that the intake during the collection day is excreted after the last collected urine void. From the sample set in this study, it was not possible to determine a quantitative intake/exposure relationship for chlorpropham.

In order to make a quantitative comparison for chlorpyrifos/chlorpyrifos-methyl, all metabolites in duplicate diets and urine need to be considered. Therefore, we summed the excretion of the measured metabolites TCPy and chlorpyrifos-methyl-desmethyl in urine and compared this to the summed intake of chlorpyrifos, chlorpyrifos-methyl, TCPy, and chlorpyrifos-methyl-desmethyl in duplicate diet. All compounds were expressed as chlorpyrifos-methyl. The quantitative comparison of intake and excretion of chlorpyrifos-methyl is shown in panel B in Fig. 2. The excretion (*y*) can be related to the intake (*x*) as *y* = 0.57*x* + 4041 with a correlation coefficient *R*^2^ = 0.77. The intercept suggests a urinary background of chlorpyrifos/chlorpyrifos-methyl, which could be caused by additional exposure through an unknown exposure route.

To assess the relation between intake of cypermethrin and permethrin to the excretion, the sum of cis- and trans-DCCA was used and expressed as cypermethrin. The common pyrethroid metabolite 3-PBA was not included in the excretion, as it is a non-specific metabolite for all pyrethroids. In some of the duplicate diets, traces of DCCA were detected, which were added to the intake, expressed as cypermethrin. In panel C in Fig. 2, the quantitative relation of intake vs excretion of cypermethrin/permethrin is shown. No clear relationship between dietary intake and urinary excretion is found. This might be explained by exposure through other routes, as permethrin is also registered for household use, in head lice treatments (shampoo), and as flea treatments for pets.

The excretion of DBCA versus intake of deltamethrin is plotted in panel D in Fig. 2. DBCA is a specific metabolite for deltamethrin; therefore, common pyrethroid metabolite 3-PBA was not included. DBCA was not detected in duplicate diets. The excretion (*y*) of DBCA can be related to the intake (*x*) of deltamethrin as *y* = 0.54*x* + 637 with a correlation coefficient *R*^2^ = 0.62. The intercept suggests a background urinary exposure to deltamethrin, through a different exposure route. Deltamethrin is registered for use in flea treatment in dogs, and as insecticide for household use. These could be possible sources of background exposure.

## Conclusion

Both duplicate diet and 24 h urine provide insight in exposure to pesticides and both have their strengths and limitations. For duplicate diets, qualitative and quantitative multi-residue analyses (parent compounds) are well established, but sample collection is a burden for study participants. Collecting urine samples, even 24 h, may be easier for participants; however, it requires ethical approval and the urine analysis currently is more challenging because metabolites are the typical target compounds for which standards are often not yet available.

The combined approach using both duplicate diets and 24 h urine for wide-scope suspect and target screening proved to be very favourable, allowing us to obtain a more comprehensive overview of pesticide intake and excretion. The combination of duplicate diet and 24 h urine made it possible to search for metabolites in urine based on a high concentration of the pesticide in the corresponding duplicate diet. Without the duplicate diet information, identifying metabolites consisting of only CHNO is difficult due to the high number of tentative detects.

Additionally, this study allowed for assessment of both qualitative and quantitative correlations between intake and excretion. For many pesticides, a good qualitative correlation between duplicate diet and 24 h urine was found. Therefore, it is concluded that urine provides valuable information on concurrent dietary exposure to pesticides.

In the quantitative comparisons between duplicate diet and 24 h urine, it was found that some metabolites in urine are already present in duplicate diet, which may give an overestimation of exposure to the parent pesticide based on measurement of the metabolites in urine samples. Determination of urinary metabolites in (processed) food, also when not part of the residue definition, should be done to improve exposure assessment through biomonitoring.

Quantitative relationships between intake and exposure for chlorpropham/4-HSA and cypermethrin+permethrin/DCCA were inconclusive. A relationship between intake and exposure of chlorpyrifos+chlorpyrifos-methyl and their metabolites in duplicate diet and urine was found; however, a urinary background was observed indicating an additional contribution to the 24 h dietary exposure. A similar result was found for deltamethrin/DBCA. For pyrethroids, additional exposure through household use or flea treatment for pets was hypothesized.

We conclude that suspect screening of 24 h urine samples has high potential to disclose concurrent pesticide exposure information in general population; however, more research on quantitative relationships between intake and exposure is needed.

### Supplementary Information

Below is the link to the electronic supplementary material.

Supplementary file1 (DOCX 221 KB)

## References

[1] van der Voet H, de Boer WJ, Kruisselbrink JW, Goedhart PW, van der Heijden GWAM, Kennedy MC (2015). The MCRA model for probabilistic single-compound and cumulative risk assessment of pesticides. Food and Chemical Toxicology..

[2] Nougadère A, Sirot V, Cravedi JP, Vasseur P, Feidt C, Fussell RJ (2020). Dietary exposure to pesticide residues and associated health risks in infants and young children — results of the French infant total diet study. Environ Int..

[3] Bouktif Zarrouk M, Gharbi E, Maatouk I, Leblanc JC, Landoulsi A (2020). Dietary exposure of Tunisian adult population aged from 19 to 65 years old to pesticides residues. Food Addit Contam Part A Chem Anal Control Expo Risk Assess..

[4] Melnyk LJ, Xue J, Brown GG, McCombs M, Nishioka M, Michael LC (2014). Dietary intakes of pesticides based on community duplicate diet samples. Science of the Total Environment..

[5] Morgan MK, MacMillan DK, Zehr D, Sobus JR (2018). Pyrethroid insecticides and their environmental degradates in repeated duplicate-diet solid food samples of 50 adults. J Expo Sci Environ Epidemiol..

[6] Rodzaj W, Wileńska M, Klimowska A, Dziewirska E, Jurewicz J, Walczak-Jędrzejowska R (2021). Concentrations of urinary biomarkers and predictors of exposure to pyrethroid insecticides in young, Polish, urban-dwelling men. Scie0nce of the Total Environment..

[7] Tarazona JV, Cattaneo I, Niemann L, Pedraza-Diaz S, Carmen González-Caballero M, De Alba-Gonzalez M, et al. A tiered approach for assessing individual and combined risk of pyrethroids using human biomonitoring data. Marike Kolossa-Gehring [Internet]. 2022;15. Available from: 10.3390/toxics10080451 10.3390/toxics10080451 PMC9416723 36006130

[8] Bradman A, Kogut K, Eisen EA, Jewell NP, Quirós-Alcalá L, Castorina R (2013). Variability of organophosphorous pesticide metabolite levels in spot and 24-hr urine samples collected from young children during 1 week. Environ Health Perspect..

[9] Knudsen LE, Hansen PW, Mizrak S, Hansen HK, Mørck TA, Nielsen F (2017). Biomonitoring of Danish school children and mothers including biomarkers of PBDE and glyphosate. Rev Environ Health..

[10] Buekers J, Remy S, Bessems J, Govarts E, Rambaud L, Riou M, et al. Glyphosate and AMPA in human urine of HBM4EU-aligned studies: part B adults. 2022; Available from: 10.3390/toxics10100552 10.3390/toxics10100552 PMC9612135 36287833

[11] Wrobel SA, Bury D, Belov VN, Klenk JM, Hauer B, Hayen H (2023). Rapid quantification of seven major neonicotinoids and neonicotinoid-like compounds and their key metabolites in human urine. Anal Chim Acta..

[12] Huber C, Nijssen R, Mol H, Philippe Antignac J, Krauss M, Brack W (2022). A large scale multi-laboratory suspect screening of pesticide metabolites in human biomonitoring: from tentative annotations to verified occurrences. Environ Int..

[13] Pourchet M, Debrauwer L, Klanova J, Price EJ, Covaci A, Caballero-Casero N (2020). Suspect and non-targeted screening of chemicals of emerging concern for human biomonitoring, environmental health studies and support to risk assessment: from promises to challenges and harmonisation issues. Environ Int..

[14] Bonvallot N, Jamin EL, Regnaut L, Chevrier C, Martin JF, Mercier F (2021). Suspect screening and targeted analyses: two complementary approaches to characterize human exposure to pesticides. Science of the Total Environment..

[15] Oerlemans A, Verscheijden LFM, Mol JGJ, Vermeulen RCH, Westerhout J, Roeleveld N (2019). Toxicokinetics of a urinary metabolite of tebuconazole following controlled oral and dermal administration in human volunteers. Arch Toxicol..

[16] Ratelle M, Coté J, Bouchard M (2015). Time profiles and toxicokinetic parameters of key biomarkers of exposure to cypermethrin in orally exposed volunteers compared with previously available kinetic data following permethrin exposure. Journal of Applied Toxicology..

[17] Meuling WJA, Opdam JJG, de Kort WLAM. Dose-excretion study with the fungicide carbendazim in volunteers [Internet]. 1993 [cited 2023 Jun 28]. Available from: http://resolver.tudelft.nl/uuid:7186ff19-9920-4dcf-88d6-8b5521bd01b8

[18] Zomer P, Mol HGJ. Simultaneous quantitative determination, identification and qualitative screening of pesticides in fruits and vegetables using LC-Q-Orbitrap^TM^-MS. Food Additives & Contaminants: Part A [Internet]. 2015 Oct 3;32(10):1628–36. Available from: http://www.tandfonline.com/doi/full/10.1080/19440049.2015.1085652 10.1080/19440049.2015.1085652 26301946

[19] Mol HGJ, Tienstra M, Zomer P (2016). Evaluation of gas chromatography – electron ionization – full scan high resolution Orbitrap mass spectrometry for pesticide residue analysis. Anal Chim Acta..

[20] EFSA. https://www.efsa.europa.eu/en/calls/consultations. Public consultations | EFSA. [internet]. [Cited 2023 Apr 24]

[21] NVWA. Individuele analyseresultaten van producten, bemonsterd bij de groothandel (januari 2018 - december 2018) (Individual analysis results of produce, wholesale, January 2018-December 2018) [Internet]. [cited 2023 Apr 24]. Available from: https://www.nvwa.nl/onderwerpen/residuen-van-bestrijdingsmiddelen-in-levensmiddelen/documenten/consument/eten-drinken-roken/bestrijdingsmiddelen/publicaties/individuele-analyseresultaten-van-producten-bemonsterd-bij-de-groothandel-januari-2018-december-2018

[22] Dutch Ministry of Agriculture N and FQ. Afzetgegevens van gewasbeschermingsmiddelen in Nederland in 2018 per werkzame stof in kg (Sales of plant protection products in the Netherlands in 2018) [Internet]. [cited 2023 Apr 25]. Available from: https://www.rijksoverheid.nl/documenten/publicaties/2022/05/19/afzetgegevens-gewasbeschermingsmiddelen-in-nederland

[23] Lommen A, Kools HJ. MetAlign 3.0: performance enhancement by efficient use of advances in computer hardware. Metabolomics. 2012;8(4):719–26. 10.1007/s11306-011-0369-1 PMC3397215 22833710

[24] Vermeulen R. Research on exposure of residents to pesticides in the Netherlands OBO flower bulbs [Internet]. [cited 2023 Apr 25]. Available from: https://www.bestrijdingsmiddelen-omwonenden.nl/documenten/onderzoeksrapport-obo-1

[25] Schymanski EL, Jeon J, Gulde R, Fenner K, Ruff M, Singer HP, et al. Identifying small molecules via high resolution mass spectrometry: communicating confidence. Vol. 48, Environ Sci Technol. 2014. p. 2097–8. 10.1021/es5002105 24476540

[26] Medina-Pastor P, Triacchini G. The 2018 European Union report on pesticide residues in food. EFSA Journal [Internet]. 2020 Apr 1 [cited 2023 Feb 23];18(4):e06057. Available from: https://onlinelibrary.wiley.com/doi/full/10.2903/j.efsa.2020.6057 10.2903/j.efsa.2020.6057 PMC7447915 32874271

[27] Lommen A (2014). Ultrafast PubChem searching combined with improved filtering rules for elemental composition analysis. Anal Chem..

[28] Arena M, Auteri D, Barmaz S, Bellisai G, Brancato A, Brocca D, et al. Peer review of the pesticide risk assessment of the active substance chlorpropham. EFSA Journal. 2017;15(7). 10.2903/j.efsa.2017.4903 PMC7009977 32625564

[29] Nougadère A, Sirot V, Kadar A, Fastier A, Truchot E, Vergnet C (2012). Total diet study on pesticide residues in France: levels in food as consumed and chronic dietary risk to consumers. Environ Int..

[30] Arena M, Auteri D, Barmaz S, Brancato A, Brocca D, Bura L, et al. Peer review of the pesticide risk assessment of the active substance cypermethrin. EFSA J. 2018 Aug;16(8). 10.2903/j.efsa.2018.5402 PMC7009394 32626035

[31] World Health Organization. A long-lasting mosquito net treated with permethrin [Internet]. [cited 2023 May 3]. Available from: https://extranet.who.int/pqweb/sites/default/files/documents/WHO_VCP_Chemicals-PERMETHRIN.pdf

[32] Medina-Pastor P, Triacchini G (2020). The 2018 European Union report on pesticide residues in food. EFSA Journal..

[33] Updated statement on the available outcomes of the human health assessment in the context of the pesticides peer review of the active substance chlorpyrifos‐methyl. EFSA J. 2019 Nov;17(11). 10.2903/j.efsa.2019.5908 PMC7008899 32626191

[34] Hernández F, Grimalt S, Pozo ÓJ, Sancho JV (2009). Use of ultra-high-pressure liquid chromatography-quadrupole time-of-flight MS to discover the presence of pesticide metabolites in food samples. J Sep Sci..

[35] Tarazona J V., Cattaneo I, Niemann L, Pedraza-Diaz S, González-Caballero MC, de Alba-Gonzalez M, et al. A tiered approach for assessing individual and combined risk of pyrethroids using human biomonitoring data. Toxics. 2022;10(8). 10.3390/toxics10080451 PMC9416723 36006130

[36] Li Y, Wang X, Feary McKenzie J, ’t Mannetje A, Cheng S, He C, et al. Pesticide exposure in New Zealand school-aged children: urinary concentrations of biomarkers and assessment of determinants. Environ Int. 2022 May 1;163. 10.1016/j.envint.2022.107206 35395578

[37] Schoeters G, Verheyen VJ, Colles A, Remy S, Martin LR, Govarts E (2022). Internal exposure of Flemish teenagers to environmental pollutants: results of the Flemish Environment and Health Study 2016–2020 (FLEHS IV). Int J Hyg Environ Health..

[38] Morgan MK, Sheldon LS, Croghan CW, Jones PA, Robertson GL, Chuang JC (2005). Exposures of preschool children to chlorpyrifos and its degradation product 3,5,6-trichloro-2-pyridinol in their everyday environments. J Expo Anal Environ Epidemiol..

