# Supplementary material for: Assessment of exposure to pesticides: residues in 24 h duplicate diets versus their metabolites in 24 h urine using suspect screening and target analysis
Source: Anal Bioanal Chem. 2023 Sep 22;416(3):635–50. doi: 10.1007/s00216-023-04918-x (PMC10766712; doi:10.1007/s00216-023-04918-x)
Supplement: Supplementary file 1 — Supplementary file1 (DOCX 221 KB) [file 216_2023_4918_MOESM1_ESM.docx]

Assessment of exposure to pesticides: residues in 24 h duplicate diets versus their metabolites in 24 h urine using suspect screening and target analysis

Nijssen R.*, Lommen A., van den Top H., van Dam R., Meuleman-Bot C., Tienstra M., Zomer P., Sunarto S., van Tricht F., Blokland M., Mol H.

Wageningen Food Safety Research, part of Wageningen University and Research, Akkermaalsbos 2, 6708 WB Wageningen, the Netherlands
*correspondence: rosalie.nijssen@wur.nl

Contents

[Sample characteristics 2](#_Toc143067653)

[Target lists duplicate diet analysis 3](#_Toc143067654)

[Duplicate diet LC-HRMS 3](#_Toc143067655)

[Duplicate diet GC-HRMS 4](#_Toc143067656)

[All detected compounds target screening duplicate diets 5](#_Toc143067657)

[Quantitative methods 7](#_Toc143067658)

[Duplicate diets – GC-MS/MS (Agilent 7890B GC + Agilent 7010B Quad MS/MS EI) 7](#_Toc143067659)

[Duplicate diets - LC/MS-MS (Shimadzu Nexera + Sciex Qtrap 6500^+^) 11](#_Toc143067660)

[24h urine – method A - LC/MSMS (Waters Acquity Classic LC +Waters Xevo TQS) 13](#_Toc143067661)

[24h urine – method B - LC/MSMS (Shimadzu Nexera + Sciex Qtrap 6500^+^) 17](#_Toc143067662)

[24h urine – method C - LC/MSMS (Shimadzu Nexera + Sciex Qtrap 6500^+^) 20](#_Toc143067663)

[Suspect list urine 24](#_Toc143067664)

# Sample characteristics

| participant# | **Net weight dup diet (g)** | **dry weight (g)** | **solid residue (%)** | **net weight urine (g)** |
| --- | --- | --- | --- | --- |
| 1 | 2153.4 | 245.5 | 16% | 1908.2 |
| 2 | 2836.9 | 252.4 | 17% | 1080.2 |
| 3 | 4469.6 | 159.4 | 10% | 2207.2 |
| 4 | 4300.8 | 143.1 | 9% | 4313.8 |
| 5 | 2917.4 | 115.2 | 7% | 2424.7 |
| 6 | 3804.5 | 223.2 | 15% | 2034.7 |
| 7 | 3745.1 | 153.9 | 10% | 2647.5 |
| 8 | 2531.2 | 200.7 | 13% | 2298.3 |
| 9 | 3920.5 | 132.3 | 8% | 1250 |
| 10 | 1545.9 | 120.6 | 8% | 3899.1 |
| 11 | 1714.8 | 190.8 | 16% | 979.4 |
| 12 | 3770.6 | 192.2 | 12% | 2903.5 |
| 13 | 3552 | 257.9 | 16% | 1634.2 |
| 14 | 4378.8 | 175.8 | 11% | 984.6 |
| 15 | 4246.3 | 167.5 | 11% | 1933.4 |
| 16 | 2278.7 | 183.9 | 12% | 1521.7 |
| 17 | 3461.2 | 303.3 | 19% | 1634.7 |
| 18 | 3586.8 | 225 | 15% | 2263 |
| 19 | 2739.1 | 333.9 | 22% | 1215.5 |
| 20 | 3666.8 | 202.3 | 13% | 2653.8 |
| 21 | 2420.2 | 254.4 | 17% | 1759.5 |
| 22 | 2695.1 | 365.6 | 23% | 1711.4 |
| 23 | 4668.5 | 148.5 | 9% | 2457.8 |
| 24 | 2556.3 | 248.4 | 16% | 2038.5 |
| 25 | 2715.6 | 213.9 | 14% | 1402 |
| 26 | 3154.3 | 203.6 | 13% | 1846.2 |
| 27 | 2408.9 | 283.4 | 18% | 1632.9 |
| 28 | 4371.1 | 162.5 | 10% | 2313.3 |
| 29 | 4498.7 | 217.8 | 14% | 910 |
| 30 | 2589.6 | 175.2 | 11% | 1281.8 |
| 31 | 2480.1 | 299.9 | 19% | 750.6 |
| 32 | 4130.4 | 154.9 | 10% | 3016.7 |
| 33 | 4637.7 | 173 | 11% | 1131 |
| 34 | 2977.1 | 180.6 | 12% | 2134.1 |
| 35 | 4342.2 | 235.9 | 15% | 2184.1 |

# Target lists duplicate diet analysis

## Duplicate diet LC-HRMS

| Abamectin | Cyazofamid | Florasulam | Mesotrione | Propamocarb | Thiacloprid |
| --- | --- | --- | --- | --- | --- |
| Acephate | Cybutryne | Fluazifop | Metalaxyl | Propiconazole | Thiamethoxam |
| Acequinocyl | Cyflufenamide | Flubendiamide | Metamitron | Propyzamide | Thiencarbazone-methyl |
| Acetamiprid | Cyflumetofen | Flucycloxuron | Metazachlor | Prosulfocarb | Thifensulfuron-methyl |
| Aclonifen | Cymoxanil | Flufenacet | Metconazole | Prosulfuron | Thiophanate-methyl |
| Aldicarb | Cyproconazole | Flufenoxuron | Methabenzthiazuron | Pymetrozine | Tolclofos-methyl |
| Ametoctradin | Cyprodinil | Flumioxazin | Methamidophos | Pyraclostrobin | Tolylfluanid |
| Amidosulfuron | Cythioate | Fluopicolide | Methiocarb | Pyridaben | Topramezone |
| Amisulbrom | Desmedipham | Fluoxastrobin | Methomyl | Pyridate | Tri-allate |
| Asulam | Dichlofluanid | Flupyrsulfuron-methyl | Methoxyfenozide | Pyrimethanil | Tribenuron-methyl |
| Azadirachtin | Difenoconazole | Fluroxypyr-meptylester | Metolachlor | Pyriproxyfen | Trifloxystrobin |
| Azamethiphos | Diflubenzuron | Flutolanil | Metoxuron | Pyroxsulam | Triflumizole |
| Azoxystrobin | Diflufenican | Fluxapyroxad | Metrafenone | Quinmerac | Triflumuron |
| Bendiocarb | Dimethenamid | Foramsulfuron | Metribuzin | Quinoclamine | Triflusulfuron-methyl |
| Bifenazate | Dimethoate | Fosthiazate | Metsulfuron-methyl | Quinoxyfen | Triforine |
| Bifenthrin | Dimethomorph | Haloxyfop-P-methyl | Mevinphos | Quizalofop-ethyl | Trinexapac-ethyl |
| Bixafen | Diuron | Hexythiazox | Myclobutanil | Rimsulfuron | Tritosulfuron |
| Boscalid | Dodemorph | Imazalil | Napropamide | Silthiofam | Zoxamide |
| Brodifacoum | Dodine | Imazamox | Nicosulfuron | Simazine | 2,4-D |
| Bromuconazole | Emamectin | Imidacloprid | Omethoate | Spinosyn-A | Bentazone |
| Bupirimate | Epoxiconazole | Indoxacarb | Oxamyl | Spinosyn-D | Bromadiolone |
| Buprofezin | Ethirimol | Iprovalicarb | Oxydemeton-methyl | Spirodiclofen | Bromoxynil |
| Carbaryl | Ethoprophos | Isoproturon | Paclobutrazol | Spiromesifen | Dinoterb |
| Carbendazim | Etoxazole | Isopyrazam | Penconazole | Spirotetramat | DNOC |
| Carbetamide | Famoxadone | Isoxaben | Pencycuron | Spiroxamine | Fluazinam |
| Carbofuran | Fenamidone | Isoxadifen-ethyl | Pendimethalin | Sulcotrione | Flubendiamide |
| Carfentrazone-ethyl | Fenamiphos | Isoxaflutole | Penflufen | Tebuconazole | Fludioxonil |
| Chlorantraniliprole | Fenhexamid | Kresoxim-methyl | Penthiopyrad | Tebufenpyrad | Fluroxypyr |
| Chlorbromuron | Fenoxaprop-p-ethyl | Lenacil | Phenmedipham | Teflubenzuron | Haloxyfop |
| Chloridazon | Fenoxycarb | Linuron | Picoxystrobin | Tembotrione | Iodosulfuron-methyl |
| Chlorpyrifos | Fenpropidin | Lufenuron | Pinoxaden | Tepraloxydim | Ioxynil |
| Clodinafop-propargyl | Fenpropimorph | Malathion | Pirimicarb | Terbuthylazine | MCPA |
| Clofentezine | Fenpyrazamine | Mandipropamid | Pirimiphos-methyl | Terbutryn | MCPP |
| Clomazone | Fipronil | Mepanipyrim | Prochloraz | Tetraconazole | Naftylacetic acid 1- |
| Clothianidin | Flonicamid | Mesosulfuron-methyl | Profenofos | Thiabendazole | Triclopyr |

## Duplicate diet GC-HRMS

| chlorpyrifos-methyl | flucythrinate |
| --- | --- |
| deltamethrin | flucythrinate II |
| chlorpropham | fluvalinate I |
| Tetrahydrophthalimide cis-1,2,3,6- (THPI) | fluvalinate (tau-) |
| ortho-phenylphenol | folpet |
| iprodion | HCB |
| bifenthrin | HCH gamma- (Lindane) |
| chlorpyrifos | o'p'-DDE |
| cyhalothrin (lambda) | o'p'-DDT |
| cypermethrin | o'p'-TDE |
| captan | Parathion |
| cyfluthrin | Parathion-methyl |
| chlorobenzilate | permethrin I |
| chloropropylate | permethrin II |
| chlorothalonil | phenothrin |
| cyphenothrin | phosmet |
| dieldrin | p'p'-DDE |
| Endosulfan-alpha | p'p'-DDT |
| Endosulfan-beta | p'p'-TDE |
| Endosulfan-sulphate | Procymidone |
| etofenprox | tefluthrin |
| fenpropathrin | tetramethrin |
| fenvalerate (esfenvalerate) | transfluthrin |
| fenvalerate | Vinclozolin |
| fipronil |  |

# All detected compounds target screening duplicate diets

| Pesticide | N pos | % pos | LC or GC | max ng/g** | median ng/g** |
| --- | --- | --- | --- | --- | --- |
| Pirimiphos-methyl | 35 | 100% | LC | 104.2 | 17.4 |
| chlorpyrifos-methyl | 33 | 94% | GC | 12.3 | 3.8 |
| deltamethrin | 30 | 86% | GC | 79.1 | 21.6 |
| chlorpropham | 26 | 74% | GC | 81.7 | 8.5 |
| Boscalid | 25 | 71% | LC | 36.7 | 5.3 |
| Trifloxystrobin | 23 | 66% | LC | 28.7 | 0.3 |
| Tebuconazole | 22 | 63% | LC | 9.0 | 0.9 |
| Carbendazim | 15 | 43% | LC | 10.2 | 0.7 |
| Pyrimethanil | 14 | 40% | LC | 468.4 | 38.6 |
| Tetrahydrophthalimide cis-1,2,3,6- (THPI) | 13 | 37% | GC | 5.2 | 2.1 |
| Cyprodinil | 12 | 34% | LC | 508.6 | 10.1 |
| ortho-phenylphenol | 12 | 34% | GC | 15.4 | 1.3 |
| Fluopyram* | 12 | 34% | LC | - | - |
| Fludioxonil | 11 | 31% | LC | 252.1 | 31.8 |
| Azoxystrobin | 10 | 29% | LC | 14.9 | 3.1 |
| Chlorantraniliprole | 9 | 26% | LC | 7.8 | 1.8 |
| Propamocarb | 9 | 26% | LC | 151.0 | 15.8 |
| Pyraclostrobin | 9 | 26% | LC | 29.8 | 6.1 |
| Epoxiconazole | 8 | 23% | LC | 2.4 | 0.4 |
| iprodion | 8 | 23% | GC | 46.2 | 10.6 |
| Acetamiprid | 6 | 17% | LC | 20.1 | 6.7 |
| Dimethomorph | 6 | 17% | LC | 5.9 | 2.3 |
| Imidacloprid | 6 | 17% | LC | 17.7 | 3.4 |
| Metalaxyl | 6 | 17% | LC | 11.6 | 1.6 |
| Thiacloprid | 6 | 17% | LC | 6.7 | 1.9 |
| bifenthrin | 6 | 17% | GC | 867.8 | 1.8 |
| Fluopicolide | 5 | 14% | LC | 3.0 | 1.9 |
| Mandipropamid | 5 | 14% | LC | 5.9 | 3.7 |
| Prosulfocarb | 5 | 14% | LC | 4.1 | 0.4 |
| chlorpyrifos | 5 | 14% | GC | 3.3 | 1.5 |
| Methoxyfenozide | 4 | 11% | LC | 11.9 | 5.0 |
| Penconazole | 4 | 11% | LC | 25.1 | 2.2 |
| Ametoctradin | 3 | 9% | LC | 40.5 | 38.7 |
| Cyproconazole | 3 | 9% | LC | 0.6 | 0.6 |
| Metrafenone | 3 | 9% | LC | 2.2 | 0.7 |
| Myclobutanil | 3 | 9% | LC | 38.2 | 9.9 |
| Thiabendazole | 3 | 9% | LC | 43.7 | 31.0 |
| Thiophanate-methyl | 3 | 9% | LC | 2.8 | 1.1 |
| cypermethrin | 2 | 6% | GC | 5.0 | 4.4 |
| Carbofuran | 2 | 6% | LC | 10.7 | 10.0 |
| Imazalil | 2 | 6% | LC | 35.4 | 33.6 |
| Thiamethoxam | 2 | 6% | LC | 9.5 | 5.4 |
| cyhalothrin (lambda) | 2 | 6% | GC | 1.8 | 1.4 |
| cyfluthrin | 1 | 3% | GC | 1.4 | 1.4 |
| Bifenazate | 1 | 3% | LC | 36.9 | 36.9 |
| Desmedipham | 1 | 3% | LC | 0.3 | 0.3 |
| Difenoconazole | 1 | 3% | LC | 1.5 | 1.5 |
| Famoxadone | 1 | 3% | LC | 1.6 | 1.6 |
| Fluxapyroxad | 1 | 3% | LC | 2.3 | 2.3 |
| Indoxacarb | 1 | 3% | LC | 2.6 | 2.6 |
| Kresoxim-methyl | 1 | 3% | LC | 14.1 | 14.1 |
| Linuron | 1 | 3% | LC | 0.6 | 0.6 |
| Metolachlor | 1 | 3% | LC | 0.0 | 0.0 |
| Metribuzin | 1 | 3% | LC | 5.3 | 5.3 |
| Phenmedipham | 1 | 3% | LC | 0.2 | 0.2 |
| Spinosyn-A | 1 | 3% | LC | 139.7 | 139.7 |
| Spiroxamine | 1 | 3% | LC | 2.0 | 2.0 |
| Sulcotrione | 1 | 3% | LC | 41.8 | 41.8 |
| captan | 1 | 3% | GC | 3.3 | 3.3 |

*) by additional retrospective screening, no semi-quantitative results available

**) concentration in freeze dried sample material (semi-quantitative)

# Quantitative methods

## Duplicate diets – GC-MS/MS (Agilent 7890B GC + Agilent 7010B Quad MS/MS EI)

chlorproham, chlorpyrifos, chlorpyrifos-methyl, cyhalothrin lambda, cypermethrin, deltamethrin, fenpropathrin, (es)fenvalerate, fluvalinate-tau, permethrin, phenothrin, tefluthrin, tetramethrin, transfluthrin

*Extraction*

A 2.5 g lyophilized duplicate diet sample was extracted with 10 ml ethyl acetate and mixed head-over-head for 30 minutes. Then, 1 g MgSO_4_ was added, the extract was vortex mixed and centrifuged 5 minutes at 3500 rpm. An aliquot of 4 mL was concentrated to 1 mL (40°C, N2 flow). PCB-137 was added as internal standard. For GPC clean up, 0.5 mL was injected on an Envirogel GPC column (19x150 mm + 19x300 mm, eluent ethyl acetate/cyclohexane 1/1, flow rate 5 mL/min). A fraction of 40 mL was collected and evaporated to 1.0 mL. PCB-198 was added as second internal standard in the GC-vial.

*Chromatography*

Inlet mode: Solvent vent

Inj. Volume: 5 µl

Front inlet temp: 70 °C

Front inlet pressure: 14.4 psi

Septum purge flow: 3 ml/min

Column: Restek Rtx-CL Pesticides (30 m x 250 µm x 0.25 µm)

Oven temp.:

|  | Rate °C/min | Value °C | Hold time | Run time min |
| --- | --- | --- | --- | --- |
| Initial |  | 60 | 2 | 2 |
| Ramp 1 | 20 | 150 | 0 | 6.5 |
| Ramp 2 | 10 | 280 | 0 | 19.5 |
| Ramp 3 | 25 | 320 | 5 | 26.1 |

Flow: 1.2 ml/min

EI source temp: 250 °C

Electron energy: 70 eV

*Detection*

MS/MS fragmentation settings:

| Component | Precursor ion | Product ion | CE | RT (min) |
| --- | --- | --- | --- | --- |
| Chlorpropham (qn) | 171 | 127 | 10 | 9.78 |
| Chlorpropham (ql) | 213 | 171 | 2 | 9.78 |
| Chlorpyrifos (qn) | 314 | 258 | 15 | 12.56 |
| Chlorpyrifos (ql) | 197 | 169 | 15 | 12.56 |
| Chlorpyrifos methyl- (qn) | 286 | 93 | 25 | 11.75 |
| Chlorpyrifos methyl- (qn) | 286 | 271 | 18 | 11.75 |
| Cypermethrin (qn) | 163 | 127 | 9 | 19.56 |
| Cypermethrin (qn) | 165 | 127 | 9 | 19.56 |
| Cyhalothrin lambda (qn) | 208 | 181 | 2 | 18.45 |
| Cyhalothrin lambda (qn) | 197 | 141 | 18 | 18.45 |
| Deltamethrin (qn) | 253 | 174 | 10 | 20.65 |
| Deltamethrin (ql) | 253 | 93 | 14 | 20.65 |
| Fenpropathrin (qn) | 265 | 210 | 10 | 17.01 |
| Fenpropathrin (ql) | 265 | 89 | 26 | 17.01 |
| (es)Fenvalerate (qn) | 419 | 225 | 2 | 20.25 |
| (es)Fenvalerate (ql) | 225 | 119 | 16 | 20.25 |
| Fluvalinate-tau (qn) | 250 | 55 | 14 | 20.67 |
| Fluvalinate-tau (ql) | 250 | 200 | 18 | 20.67 |
| Permethrin (qn) | 181 | 168 | 16 | 18.05 |
| Permethrin (ql) | 163 | 127 | 10 | 18.05 |
| Phenothrin (qn) | 123 | 81 | 5 | 16.60 |
| Phenothrin (ql) | 183 | 153 | 12 | 16.60 |
| Tefluthrin (qn) | 177 | 127 | 15 | 11.73 |
| Tefluthrin (ql) | 197 | 141 | 10 | 11.73 |
| Tetramethrin (qn) | 164 | 136 | 2 | 19.20 |
| Tetramethrin (ql) | 164 | 107 | 14 | 19.20 |
| Transfluthrin (qn) | 163 | 143 | 5 | 12.30 |
| Transfluthrin (ql) | 335 | 163 | 10 | 12.30 |

*Quantification*

Quantification was performed by bracketing 1-point calibration, using a matrix-standard prepared from a wheat sample (5 ng/mL, corresponding to 10 ng/g). Linearity of the pesticide response vs concentration had been verified in the range 0.5-100 ng/mL.

*Verification method performance*

For quantitative GC-MS/MS-based analysis an existing validated method for determination of pesticides in feed materials (intermediate fat/low water) was used. To demonstrate its applicability to the freeze dried duplicate diet material, sub-portions of two duplicate diet samples were spiked in triplicate, one set at 2 µg/kg, and the other set at 10 µg/kg were included in the batch.

The average recovery and repeatability (n=3) at the two spike levels are summarized in the table below. In most cases, these complied with the criteria outline in SANTE/12682-2019 (70-120% for recovery, RSD≤20%), and based on that it was concluded that the method was fit-for-purpose.

The method LOD for the duplicate diet samples was estimated from the lower spike level, using the signal to noise ratio of 3 as definition. The LODs ranged from 0.2-1 ng/g (individual LODs are provided in the results table).

|  | 2 ng/g | | 10 ng/g | |
| --- | --- | --- | --- | --- |
|  | Recovery | RSD | Recovery | RSD |
| Chlorpropham | - (blank too high) | | 90% | 8% |
| Chlorpyrifos | 90% | 2% | 74% | 6% |
| Chlorpyrifos methyl- | 85% | 2% | 91% | 8% |
| Cyhalothrin lambda | 78% | 10% | 73% | 13% |
| Cypermethrin | 99% | 11% | 70% | 13% |
| Deltamethrin | 115% | 14% | 77% | 14% |
| Fenpropathrin | 79% | 10% | 71% | 12% |
| Fenvalerate | 53% | 14% | 73% | 14% |
| Fluvalinate-tau | 26% | 7% | 20% | 15% |
| Permethrin | 91% | 8% | 72% | 12% |
| Phenothrin | 80% | 15% | 75% | 16% |
| Tefluthrin | 35% | 7% | 32% | 14% |
| Tetramethrin | 69% | 7% | 71% | 16% |
| Transfluthrin | 79% | 9% | 67% | 12% |

*Results*

| sample | chlorproham | chlorpyrifos | chlorpyrifos-methyl | cyhalothrin lambda | cypermethrin | deltamethrin | fenpropathrin | fenvalerate | fluvalinate tau | permethrin | phenothrin | tetramethrin | tefluthrin | transfluthrin |
| --- | --- | --- | --- | --- | --- | --- | --- | --- | --- | --- | --- | --- | --- | --- |
| Concentration in ng/g lyophilized duplicate diet material* | | | | | | | | | | | | | | |
| 1 | 92.5 | 0.20 | 8.64 | < | 0.95 | 5.35 | < | < | < | 0.87 | < | < | < | < |
| 2 | 2.44 | 0.28 | 1.16 | < | 0.97 | < | < | < | < | 0.64 | < | < | < | < |
| 3 | 15.6 | 0.18 | 11.0 | < | 4.66 | 3.15 | < | < | < | 1.00 | < | < | < | < |
| 4 | 35.1 | 0.32 | 0.69 | < | 0.92 | < | < | < | < | 0.39 | < | < | < | < |
| 5 | 4.48 | 0.34 | 2.84 | < | 2.64 | 4.27 | < | < | < | 0.48 | < | < | < | < |
| 6 | 3.50 | 0.28 | 2.65 | < | 3.18 | 2.95 | < | < | < | 0.71 | < | < | < | < |
| 7 | 2.51 | 0.31 | 1.61 | < | 4.48 | < | < | < | < | 0.99 | < | < | < | < |
| 8 | 9.15 | 0.22 | 4.76 | < | 1.02 | 3.20 | < | < | < | 0.57 | < | < | < | < |
| 9 | 14.6 | 0.22 | 13.2 | < | 5.03 | 3.86 | < | < | < | 0.72 | < | < | < | < |
| 10 | 1.33 | 0.28 | 2.26 | < | 1.48 | 1.79 | < | < | < | 1.38 | < | < | < | < |
| 11 | 12.0 | 0.17 | 10.4 | < | 1.81 | 5.15 | < | < | < | 0.44 | < | < | < | < |
| 12 | 0.63 | 0.22 | 2.49 | < | 3.45 | < | < | < | < | 0.53 | < | < | < | < |
| 13 | 1.25 | 0.13 | 2.25 | < | 2.73 | 13.6 | < | < | < | 0.50 | < | < | < | < |
| 14 | 0.83 | 0.15 | 4.16 | < | 3.29 | 13.4 | < | < | < | 0.58 | < | < | < | < |
| 15 | 1.22 | 0.38 | 2.77 | < | 1.70 | 3.05 | < | < | < | 1.05 | < | < | < | < |
| 16 | 1.56 | 0.39 | 3.17 | < | 2.73 | < | < | < | < | 0.74 | < | < | < | < |
| 17 | 7.43 | 0.33 | 3.28 | < | 11.75 | 5.03 | < | < | < | 0.35 | < | < | < | < |
| 18 | 4.53 | 0.68 | 4.77 | < | 4.35 | 3.51 | < | < | < | 0.40 | < | < | < | < |
| 19 | 0.53 | 0.22 | 6.11 | < | 1.20 | 2.22 | < | < | < | 0.50 | < | < | < | < |
| 20 | 0.46 | 0.24 | 4.38 | < | 2.11 | < | < | < | < | 0.35 | < | < | < | < |
| 21 | 0.52 | 0.26 | 4.67 | < | 0.80 | 1.21 | < | < | < | < | < | < | < | < |
| 22 | 6.29 | 0.61 | 6.19 | < | 2.18 | 7.11 | < | < | < | 0.23 | < | < | < | < |
| 23 | 0.53 | 0.86 | 11.3 | < | 4.22 | 6.74 | < | < | < | 0.33 | < | < | < | < |
| 24 | 12.1 | 0.30 | 5.83 | < | 3.95 | 2.67 | < | < | < | 0.32 | < | < | < | < |
| 25 | 8.52 | 0.30 | 6.65 | < | 6.13 | 5.86 | < | < | < | 0.25 | < | < | < | < |
| 26 | 5.49 | 0.24 | 3.72 | < | 5.04 | 3.89 | < | < | < | < | < | < | < | < |
| 27 | 30.7 | 0.26 | 4.62 | < | 6.05 | 2.56 | < | < | < | 0.30 | < | < | < | < |
| 28 | 10.8 | 0.24 | 3.83 | < | 3.95 | 4.35 | < | < | < | 0.73 | < | < | < | < |
| 29 | 19.7 | 0.89 | 10.3 | < | 3.41 | 6.13 | < | < | < | 0.35 | < | < | < | < |
| 30 | 5.39 | 0.43 | 2.12 | < | 1.16 | 10.8 | < | < | < | 0.51 | < | < | < | < |
| 31 | 39.2 | 0.21 | 3.90 | < | 1.69 | < | < | < | < | 0.37 | < | < | < | < |
| 32 | 11.8 | 0.31 | 3.41 | < | 0.79 | < | < | < | < | 0.30 | < | < | < | < |
| 33 | 23.3 | 0.44 | 3.46 | < | 2.12 | 5.00 | < | < | < | 0.39 | < | < | < | < |
| 34 | 6.47 | 0.85 | 5.27 | < | 3.66 | 5.07 | < | < | < | 0.48 | < | < | < | < |
| 35 | 7.45 | 1.43 | 4.13 | < | 5.64 | < | < | < | < | < | < | < | < | < |
| LOD | 0.5 | 0.2 | 0.2 | 2 | 0.3 | 1 | 0.1 | 5 | 0.1 | 0.2 | 2 | 0.5 | 0.25 | 0.25 |

* concentrations < 1 ng/g are below the lowest verified range of linearity, and should be considered as semi-quantitative.

## Duplicate diets - LC/MS-MS (Shimadzu Nexera + Sciex Qtrap 6500^+^)

3-PBA, 4-F-3-PBA, TCPy, chlorpyrifos-desethyl, chlorpyrifos-methyl-desmethyl, DCCA, DBCA,

*Extraction*

Isotope labelled internal standards trans-DCCA-d6, cis-DCCA-13C2-D1, DBCA-13C2-D1, 13C6-3-PBA, 13C6-4-F-3-PBA and 13C3_TCPy were added at 10 ng/g level to 2.5 g sample, before 5 ml water and 5 ml ACN 1% HAc were added. After shaking head-over-head for 30 minutes, 2 g MgSO_4_ and 0.5 g NaAc were added and extracts were vortex mixed for 30 seconds and centrifuged for 10 min at 3500 rpm. 200 µl of the ACN extract was diluted with 200 µl water and filtered before injection in LC-MS/MS.

*Chromatography*

Column: Waters Acquity HSS T3, 2.1x100 mm, 1.7 µm

Column temp.: 40 °C

Inj. Vol.: 5 µl

Flow: 0.4 ml/min

Mobile phase A: 0.1 % Formic Acid in water

Mobile phase B: 0.1 % Formic Acid in acetonitrile

Gradient:

| Time (min) | % A | % B |
| --- | --- | --- |
| 0 | 80 | 20 |
| 1 | 80 | 20 |
| 6 | 0 | 100 |
| 7 | 0 | 100 |
| 7.5 | 80 | 20 |
| 9.5 | 80 | 20 |

*Detection*

Ionisation mode: negative

Curtain gas: 30

Collision gas: -2

Ion spray voltage: -4000 V

Temperature: 300 °C

Ion Source gas 1: 60

Ion Source gas 2: 60

MS/MS fragmentation settings:

| Component | Q1 mass | Q3 mass | EP | DP | CE | CXP |
| --- | --- | --- | --- | --- | --- | --- |
| CP-DE (qn) | 319.8 | 195.8 | -10 | -20 | -22 | -15 |
| CP-DE (ql) | 321.8 | 197.8 | -10 | -20 | -22 | -15 |
| CPM-DM (qn) | 305.8 | 195.8 | -10 | -20 | -22 | -15 |
| CPM-DM (ql) | 307.8 | 197.8 | -10 | -20 | -22 | -15 |
| cis/trans-DCCA (qn) | 207 | 35 | -10 | -50 | -36 | -7 |
| cis/trans-DCCA (ql) | 209 | 37 | -10 | -50 | -36 | -7 |
| trans-DCCA-D6 | 213 | 35 | -10 | -50 | -36 | -9 |
| cis-DCCA-13C2-D1 | 210 | 35 | -10 | -50 | -36 | -7 |
| DBCA (qn) | 297 | 79 | -10 | -30 | -30 | -11 |
| DBCA (ql) | 295 | 79 | -10 | -30 | -30 | -15 |
| DBCA-13C2-D1 | 300 | 79 | -10 | -30 | -30 | -11 |
| 3-PBA (qn) | 213 | 93 | -10 | -40 | -36 | -9 |
| 3-PBA (ql) | 214 | 94 | -10 | -40 | -36 | -9 |
| 13C6_3-PBA | 219 | 99 | -10 | -40 | -36 | -9 |
| 4-F-3-PBA (qn) | 230.8 | 93 | -10 | -35 | -40 | -7 |
| 4-F-3-PBA (ql) | 230.8 | 187 | -10 | -35 | -18 | -23 |
| 13C6_4-F-3-PBA | 236.8 | 99 | -10 | -35 | -40 | -7 |
| TCPy (qn) | 195.8 | 35 | -10 | -40 | -38 | -9 |
| TCPy (ql) | 197.8 | 35 | -10 | -40 | -42 | -15 |
| 13C3_TCPy | 198.8 | 35 | -10 | -40 | -38 | -9 |

*Quantification*

Pyrethroids metabolites and TCPy were quantified against solvent standards, after normalization of the response to the isotope labelled internal standards. For quantification of chlorpyrifos-desethyl (CP-DE) and chlopryrifos-methyl-desmethyl (CPM-DM) standard addition to the extracts was performed at 10 µg/kg and 50 µg/kg, as no internal standards for these compounds were available.

*Validation*

A simplified method validation was performed for this method. One duplicate diet sample was spiked in triplicate at three concentration levels. The low level was 1 ng/g for all compounds, the mid-level was 2 ng/g for pyrethroid metabolites and 5 ng/g for CP-DE and CPM-DM, and the high level was 10 ng/g for pyrethroid metabolites and 50 ng/g for CP-DE and CPM-DM.

A calibration curve (0, 0.05, 0.1, 0.25, 0.5, 1, 2.5, 5, 10 and 25 ng/ml) in solvent (1% HAc in ACN/water 50/50) was used to evaluate linearity. The criterion for linearity is that back calculated concentrations should be ≤20%. DCCA-trans, 3-PBA and 4-F-3-PBA were linear in the complete evaluated range. For CP-DE, CPM-DM and TPCy the response was linear between 0-10 ng/g. For DCCA-cis the response was linear between 0.1 and 10 ng/ml and for DBCA from 0.5-5 ng/ml.

Trueness was determined through recovery experiment. The average recovery should be between 70-120%. The precision was determined as the repeatability relative standard deviation (RSD) of the recovery obtained should be ≤20% (SANTE/12682-2019). The criteria were met in most cases, the method was considered fit-for-purpose.

|  | DCCA-cis | DCCA-trans | DBCA | 3-PBA | 4-F-3-PBA | TCPy | CP-DE | CPM-DM |
| --- | --- | --- | --- | --- | --- | --- | --- | --- |
| Low (1 ng/g) | | | | | | | | |
| recovery | 109% | 123% | 138% | 100% | 108% | 103% | 96% | 91% |
| RSD | 8.9% | 14.3% | 12.1% | 8.6% | 2.2% | 5.3% | 6.7% | 7.9% |
| Mid (2 / 5 ng/g) | | | | | | | | |
| recovery | 96% | 122% | 115% | 113% | 109% | 104% | 87% | 91% |
| RSD | 13.7% | 8.5% | 21.8% | 1.7% | 1.2% | 2.8% | 11.4% | 12.8% |
| High (10 / 50 ng/g) | | | | | | | | |
| recovery | 105% | 130% | 88% | 109% | 107% | 96% | 99% | 101% |
| RSD | 10.6% | 4.6% | 8.9% | 2.6% | 3.3% | 1.3% | 11.8% | 3.1% |

The method LOD for the duplicate diet samples was estimated from the lower spike level, using the signal to noise ratio of 3 as definition. The LODs ranged from 0.1-0.5 ng/g (individual LODs are provided in the results table).

*Results*

| Sample | DCCA cis | DCCA trans | DBCA | 3-PBA | 4-F-3-PBA | TCPy | CP-DE | CPM-DM |
| --- | --- | --- | --- | --- | --- | --- | --- | --- |
| 1 | < | < | < | < | < | 4.14 | 0.08 | 6.50 |
| 2 | < | < | < | 0.21 | < | 1.66 | < | 0.14 |
| 3 | < | 0.27 | < | 0.30 | < | 7.77 | < | 20.3 |
| 4 | < | < | < | < | < | 0.9 | 0.07 | 0.19 |
| 5 | < | < | < | < | < | 6.11 | < | 9.25 |
| 6 | < | < | < | 0.35 | < | 4.01 | < | 8.36 |
| 7 | < | < | < | 0.28 | < | 2.52 | < | 1.69 |
| 8 | < | < | < | 0.21 | < | 1.33 | < | 2.33 |
| 9 | < | < | < | 0.24 | < | 11.3 | < | 34.4 |
| 10 | < | < | < | 0.35 | < | 2.58 | < | 2.94 |
| 11 | < | < | < | 0.21 | < | 6.81 | < | 33.1 |
| 12 | < | < | < | 0.29 | < | 1.89 | < | 1.60 |
| 13 | < | < | < | < | < | 1.01 | < | 1.94 |
| 14 | < | < | < | < | < | 4.67 | < | 13.9 |
| 15 | < | < | < | 0.20 | < | 4.93 | 0.19 | 6.86 |
| 16 | < | < | < | < | < | 4.64 | < | 7.24 |
| 17 | < | < | < | 0.23 | < | 1.83 | < | 3.87 |
| 18 | < | < | < | 0.23 | < | 8.58 | 0.15 | 14.9 |
| 19 | < | < | < | < | < | 4.93 | < | 9.49 |
| 20 | < | < | < | < | < | 2.44 | < | 3.38 |
| 21 | < | 0.34 | < | < | < | 0.7 | < | 0.87 |
| 22 | < | 0.28 | < | 0.28 | < | 11.4 | 0.10 | 18.7 |
| 23 | < | 0.75 | < | 0.74 | < | 19.6 | < | 23.8 |
| 24 | < | < | < | < | < | 7.76 | < | 13.6 |
| 25 | < | 0.27 | < | 0.23 | < | 9.30 | < | 19.8 |
| 26 | < | < | < | < | < | 2.24 | < | 9.47 |
| 27 | < | < | < | < | < | 2.30 | 0.06 | 3.34 |
| 28 | < | 0.27 | < | < | < | 1.78 | < | 3.48 |
| 29 | < | < | < | < | < | 10.4 | 0.29 | 30.3 |
| 30 | < | < | < | 0.30 | < | 1.03 | < | 0.17 |
| 31 | < | 0.38 | < | < | < | 3.76 | < | 8.05 |
| 32 | < | < | < | < | < | 1.09 | < | 1.58 |
| 33 | < | < | < | 0.32 | < | 2.41 | < | 0.94 |
| 34 | < | < | < | 0.32 | < | 11.2 | 0.10 | 14.1 |
| 35 | < | 0.34 | < | 0.27 | < | 2.27 | 0.10 | 1.82 |
| LOD | 0.5 | 0.25 | 0.5 | 0.2 | 0.1 | 0.25 | 0.1 | 0.1 |

* concentrations < 0.2 ng/g are below the lowest verified range of linearity, and should be considered as semi-quantitative.

## 24h urine – method A - LC/MSMS (Waters Acquity Classic LC +Waters Xevo TQS)

4-HSA (biomarker of chlorpropham)

*Extraction*

This method consisted of addition of internal standard 4-HSA-d7 at 10 ng/ml to the urine sample and ultrafiltration using Amicon Ultra 30kDA filter units. The filtered urine was transferred to an LC vial and injected as such.

*Chromatography*

Column: Waters Acquity HSS T3, 2.1x100 mm, 1.7 µm

Column temp.: 45 °C

Inj. Vol.: 20 µl

Flow: 0.4 ml/min

Mobile phase A: 2 mM ammonium formate 1 mM ammonium fluoride in MilliQ water + 20 µl formic acid/liter

Mobile phase B: 2 mM ammonium formate 1 mM ammonium fluoride in methanol/MilliQ water (95/5 v/v) + 20 µl formic acid/liter

Gradient:

| Time (min) | % A | % B |
| --- | --- | --- |
| 0 | 100 | 0 |
| 8.5 | 0 | 100 |
| 11.5 | 0 | 100 |
| 12 | 100 | 0 |
| 14 | 100 | 0 |

*Detection*

Ionization mode: Negative

Capillary (kV) 2.50

Source Temp (°C) 120

Desolvation Temp (°C) 350

Cone Gas Flow (L/Hr) 207

Desolvation Gas Flow (L/Hr) 510

| Name | tr | Quantifier | Cone | Collision | Qualifier | Cone | Collision |
| --- | --- | --- | --- | --- | --- | --- | --- |
| 4-HSA | 5.73 | 308.2>141 | 20 | 34 | 310.2>143 | 20 | 20 |
| 4-HSA-d7 | 5.83 | 317.2>143 | 20 | 20 | 315.2>141 | 20 | 34 |

*Quantification*

4-HSA was quantified against matrix matched standards, after normalization of the response to the isotope labelled internal standards. For results outside the validated linear range, dilution and reanalyzes were performed.

*Validation*

This method was validated according to SANTE/12682-2019. In the method validation urine samples were analyzed as such and spiked at 0.1ng/ml; 1 ng/ml and 10 ng/ml level. Quantitation was performed using standards in matrix.

Six urine samples were used in the validation. In method development it was noted that most samples contain at least a small amount of 4-HSA. The samples used for validation were analyzed to determine the level of 4-HSA. For the lowest validation level, only 2 suitable samples were found. These were spiked and analyzed in duplicate and triplicate to yield 5 samples at the 0.1 ng/ml level. At 1 ng/ml level, 3 samples were suitable. Therefore, two samples were spiked and analyzed in duplicate. At 10 ng/ml level, 5 different samples were used.

Calibration curves in water and in urine were used to evaluate linearity. 4-HSA was linear for the evaluated linear range. The specificity criterion requires that the signal (peak area) of interfering compounds in the non-fortified sample should not exceed 30% of the peak in the spiked samples. No specificity issues were observed, peaks in reagent blanks were <5%.

Trueness was determined through recovery experiments at 0.1, 1 and 10 ng/ml level. For quantification a standard in urine at 2 ng/ml level was used. The average recovery should be between 70-120%. The precision during this initial validation was determined as the repeatability relative standard deviation (RSD) of the recovery obtained for the fortified samples. The repeatability should be ≤20%. For 4-HSA, trueness and precision criteria were met at the three validation levels.

| Compound | 0.1 ng/ml | | 1 ng/ml | | 10 ng/ml | |
| --- | --- | --- | --- | --- | --- | --- |
|  | recovery | rsd | recovery | rsd | recovery | rsd |
| 4-HSA | 117% | 19% | 95% | 19% | 90% | 15% |

The method performance criteria are met for analysis of 4-HSA in human urine for all 3 validated levels.

*Results*

| Sample | 4-HSA ng/ml |
| --- | --- |
| 1 | 89.61 |
| 2 | < |
| 3 | < |
| 4 | 7.04 |
| 5 | < |
| 6 | 0.74 |
| 7 | 0.26 |
| 8 | < |
| 9 | 71.9 |
| 10 | 0.25 |
| 11 | 0.11 |
| 12 | < |
| 13 | < |
| 14 | 0.40 |
| 15 | 0.16 |
| 16 | 0.79 |
| 17 | 0.36 |
| 18 | 1.72 |
| 19 | < |
| 20 | 0.42 |
| 21 | 0.59 |
| 22 | 48.1 |
| 23 | < |
| 24 | 2.08 |
| 25 | 6.12 |
| 26 | < |
| 27 | 30.5 |
| 28 | < |
| 29 | 4.69 |
| 30 | 0.30 |
| 31 | 11.7 |
| 32 | < |
| 33 | 29.3 |
| 34 | 0.16 |
| 35 | 0.24 |
| LOQ | 0.10 |

## 24h urine – method B - LC/MSMS (Shimadzu Nexera + Sciex Qtrap 6500^+^)

chlorpyrifos-desethyl (CP-DE) and chlopryrifos-methyl-desmethyl (CPM-DM)

*Extraction*

Urine (200 µL) was diluted with an equal volume of ACN 1% FA. After filtration, the sample was injected in the LC-MS/MS.

*Chromatography*

Column: Acquity HSS T3, 2.1x100 mm,

Column temp.: 40 °C

Inj. Vol.: 5 µl

Flow: 0.4 ml/min

Eluents: A : 0.1 % formic acid in water

B: 0.1 % formic acid in acetonitrile

Gradient:

| Time (min) | % A | % B |
| --- | --- | --- |
| 0 | 80 | 20 |
| 1 | 80 | 20 |
| 6 | 0 | 100 |
| 7 | 0 | 100 |
| 7.5 | 80 | 20 |
| 9.5 | 80 | 20 |

*Detection*

Ionisation mode: negative

Curtain gas: 30

Collision gas: -2

Ion spray voltage: -4000 V

Temperature: 300 °C

Ion Source gas 1: 60

Ion Source gas 2: 60

MS/MS fragmentation settings

| Component | Q1 mass | Q3 mass | EP | DP | CE | CXP |
| --- | --- | --- | --- | --- | --- | --- |
| CP-DE (qn) | 319.8 | 195.8 | -10 | -20 | -22 | -15 |
| CP-DE (ql) | 321.8 | 197.8 | -10 | -20 | -22 | -15 |
| CPM-DM (qn) | 305.8 | 195.8 | -10 | -20 | -22 | -15 |
| CPM-DM (ql) | 307.8 | 197.8 | -10 | -20 | -22 | -15 |

*Quantification*

Quantification was performed using standard addition at 2 ng/mL and 10 ng/mL.

*Validation*

A simplified method validation was performed for this method. One sample containing CMP-DM was analyzed in triplicate and one other sample was spiked at 0.5 ng/ml in triplicate. Additionally, linearity and specificity were assessed.

A calibration curve (0, 0.05, 0.1, 0.25, 0.5, 1, 2.5, 5 and 10 ng/ml) in solvent (1% FA in ACN/water 50/50) was used to evaluate linearity. The criterion for linearity is that residuals should be ≤20%. For CP-DE the response was linear over a range 0.05-10 ng/ml, and for CPM-DM over a range of 0.1-10 ng/ml.

The signal (peak area) of interfering compounds in the non-fortified sample should not exceed 30% of the peak in the spiked samples. No specificity issues were observed, peaks in reagent blanks were <5%.

Trueness was determined through recovery experiment at 0.5 ng/ml. The average recovery should be between 70-120%. The precision was determined as the repeatability relative standard deviation (RSD) of the recovery obtained should be below 20%. For the triplicate analysis of the positive CPM-DM sample, the RSD was also calculated.

| Compound | 0.5 ng/ml | | Pos sample triplicate | |
| --- | --- | --- | --- | --- |
|  | recovery | rsd |  | rsd |
| CPM-DM | 73% | 8.3% |  |  |
| CP-DE | 102% | 3.1% |  | 2.6% |

Conclusions

The method is fit for purpose.

*Results*

| Sample | CP-DE | CPM-DM |
| --- | --- | --- |
| 1 | < | 0.52 |
| 2 | < | 0.31 |
| 3 | < | 2.18 |
| 4 | < | 0.17 |
| 5 | < | 0.57 |
| 6 | < | 1.35 |
| 7 | < | 0.12 |
| 8 | < | 0.31 |
| 9 | < | 1.65 |
| 10 | < | 0.16 |
| 11 | < | 4.35 |
| 12 | < | 0.53 |
| 13 | < | 0.27 |
| 14 | < | 2.23 |
| 15 | < | 0.53 |
| 16 | < | 0.8 |
| 17 | < | 0.96 |
| 18 | < | 1.39 |
| 19 | < | 3.74 |
| 20 | < | 0.64 |
| 21 | < | 0.27 |
| 22 | < | 4.01 |
| 23 | < | 2.26 |
| 24 | < | 1.09 |
| 25 | < | 2.88 |
| 26 | < | 1.03 |
| 27 | < | 0.57 |
| 28 | < | 0.35 |
| 29 | < | 8.34 |
| 30 | < | < |
| 31 | < | 1.15 |
| 32 | < | 0.22 |
| 33 | < | 0.56 |
| 34 | < | 1.28 |
| 35 | < | 0.65 |
| LOD | 0.1 | 0.1 |

## 24h urine – method C - LC/MSMS (Shimadzu Nexera + Sciex Qtrap 6500^+^)

3-PBA, 4-F-3-PBA, DBCA, cis-DCCA, trans-DDCA, TCPy

*Extraction*

In method C 5 ml of urine was added to 0,5 ml acetate buffer (pH=4.5), 50 µl of β-glucuronidase/arylsulfatase from Helix Pomatia and internal standards for each of the analytes. Samples were deconjugated in a water bath overnight at 37 °C. The following morning, samples were allowed to cool to room temperature and an SPE clean-up was performed using Phenomenex Strata-X polymeric reversed phase cartridges (200 mg/6 ml). After conditioning of the cartridge with methanol and water, the sample was loaded. The cartridge was washed with 10 ml water 1% FA and 10 mL 50% MeOH 1% FA and eluted with 6 ml acetone. To the acetone extract, 10 µl DMSO was added and the samples were evaporated at 40 °C under a gentle stream of nitrogen. The extracts were reconstituted in 0.5 ml 20% ACN.

*Chromatography*

Column: Waters Acquity HSS T3, 2.1x100 mm, 1.7 µm

Column Temp: 40 °C

Injection volume: 50 µL

Flow: 0.4 mL/min

Eluents: A: 0.1% formic acid in water
 B: 0.1% formic acid in acetonitrile

Gradient:

| Time (min) | % A | % B |
| --- | --- | --- |
| 0 | 80 | 20 |
| 1 | 80 | 20 |
| 6 | 0 | 100 |
| 7 | 0 | 100 |
| 7.5 | 80 | 20 |
| 9.5 | 80 | 20 |

*Detection*

Ionisation mode: negative

Curtain gas: 30

Collision gas: -2

Ion spray voltage: -4000 V

Temperature: 300 °C

Ion Source gas 1: 60

Ion Source gas 2: 60

| Analyte | Q1 (m/z) | Q3 (m/z | DP (V) | EP (V) | CE (V) | CXP (V) |
| --- | --- | --- | --- | --- | --- | --- |
| ClF3CA (qn) | 241.2 | 204.9 | -20 | -10 | -10 | -10 |
| ClF3CA (ql) | 241.2 | 35 | -20 | -10 | -25 | -10 |
| cis/trans-DCCA (qn) | 207 | 35 | -50 | -10 | -36 | -7 |
| cis/trans-DCCA (ql) | 209 | 37 | -50 | -10 | -36 | -7 |
| trans-DCCA-D6 | 213 | 35 | -50 | -10 | -36 | -9 |
| cis-DCCA-13C2-D1 | 210 | 35 | -50 | -10 | -36 | -7 |
| DBCA (qn) | 297 | 79 | -30 | -10 | -30 | -11 |
| DBCA (ql) | 295 | 79 | -30 | -10 | -30 | -15 |
| DBCA-13C2-D1 | 300 | 79 | -30 | -10 | -30 | -11 |
| 3-PBA (qn) | 213 | 93 | -40 | -10 | -36 | -9 |
| 3-PBA (ql) | 214 | 94 | -40 | -10 | -36 | -9 |
| 13C6_3-PBA | 219 | 99 | -40 | -10 | -36 | -9 |
| 4-F-3-PBA (qn) | 230.8 | 93 | -35 | -10 | -40 | -7 |
| 4-F-3-PBA (ql) | 230.8 | 187 | -35 | -10 | -18 | -23 |
| 13C6_4-F-3-PBA | 236.8 | 99 | -35 | -10 | -40 | -7 |
| TCPy (qn) | 195.8 | 35 | -40 | -10 | -38 | -9 |
| TCPy (ql) | 197.8 | 35 | -40 | -10 | -42 | -15 |
| 13C3_TCPy | 198.8 | 35 | -40 | -10 | -38 | -9 |

*Quantification*

Pyrethroids metabolites and TCPy were quantified against solvent standards, after normalization of the response to the isotope labelled internal standards.

*Validation*

This method was validated according to the criteria in SANTE/12682-2019. In initial method validation urine samples were analyzed as such and spiked at 0.1ng/ml; 0.5 ng/ml and 2.5 ng/ml level. A reagent blank was included to assess selectivity.

In method development it was noted that most samples contain at least a small amount of metabolites. Therefore, the lowest validation level was performed using synthetic urine. The other levels were validated using urine samples, 1 urine sample was spiked in fivefold for 0.5 ng/ml level and 6 other urine samples were spiked at 2.5 ng/ml level. All urines are also analyzed without spike (blank).

Linearity was assessed in a range of 0.5-50 ng/ml in solvent, corresponding to 0.05-5 ng/ml in urine, using seven calibration points. The criterion for linearity is that the back-calculated concentrations should deviate less than 20% from the actual concentrations. For all compounds, the response was linear over the concentration range tested.

The selectivity criterion requires that the signal (peak area) of interfering compounds in the non-fortified sample should not exceed 30% of the peak in the spiked samples. No selectivity issues were observed.

Trueness was determined through recovery experiments at the 0.1, 0.5 and 2.5 ng/ml level. The average recovery should be between 70-120%. The precision during this initial validation was determined as the repeatability relative standard deviation (RSD) of the recovery obtained for the fortified samples, and should be ≤20%.

| Compound | Concentration interval / range | Unit | Recovery (%) | St. dev. Repeatability (s_r_) | Rel st. dev. Repeatability  (RSDr-%_r_) | LOQ (ng/ml) |
| --- | --- | --- | --- | --- | --- | --- |
| cis-DCCA | 0.1 | ng/ml | 119 | 8.2 | 6.8 | 0.1 |
|  | 0.5 | ng/ml | 107 | 6.3 | 5.9 |  |
|  | 2.5 | ng/ml | 101 | 6.4 | 6.3 |  |
| trans-DCCA | 0.1 | ng/ml | 64 | 5.6 | 8.8 | 0.1 |
|  | 0.5 | ng/ml | 89 | 3.6 | 4.1 |  |
|  | 2.5 | ng/ml | 78 | 9.7 | 12.4 |  |
| DBCA | 0.1 | ng/ml | 107 | 8.4 | 7.9 | 0.1 |
|  | 0.5 | ng/ml | 114 | 4.8 | 4.2 |  |
|  | 2.5 | ng/ml | 95 | 12.3 | 16.1 |  |
| 3-PBA | 0.1 | ng/ml | 102 | 2.0 | 1.9 | 0.1 |
|  | 0.5 | ng/ml | 110 | 3.5 | 3.2 |  |
|  | 2.5 | ng/ml | 111 | 1.7 | 1.5 |  |
| 4-F-3_PBA | 0.1 | ng/ml | 101 | 3.2 | 3.1 | 0.1 |
|  | 0.5 | ng/ml | 112 | 2.3 | 2.0 |  |
|  | 2.5 | ng/ml | 111 | 2.2 | 2.0 |  |
| TCPy | 0.1 | ng/ml | 109 | 4.5 | 4.2 | 0.1 |
|  | 0.5 | ng/ml | 91 | 3.2 | 3.5 |  |
|  | 2.5 | ng/ml | 76 | 9.5 | 12 |  |

Conclusions

The method performance criteria are met for analysis of all listed compounds.*Results*

| Sample | DCCA_trans | DCCA_cis | DCCA sum | DBCA | 3-PBA | TCPy | 4-F-3-PBA |
| --- | --- | --- | --- | --- | --- | --- | --- |
| 1 | 0.32 | 0.14 | 0.49 | 0.30 | 0.33 | 2.61 | < |
| 2 | 0.31 | 0.21 | 0.54 | 0.15 | 0.30 | 2.98 | < |
| 3 | 0.35 | 0.23 | 0.60 | 0.34 | 0.43 | 2.52 | < |
| 4 | < | < | 0.11 | < | < | 0.55 | < |
| 5 | 0.14 | 0.10 | 0.25 | 0.22 | 0.25 | 2.04 | < |
| 6 | 0.40 | 0.25 | 0.68 | 0.74 | 0.51 | 2.90 | < |
| 7 | 0.19 | 0.11 | 0.32 | < | 0.19 | 1.17 | < |
| 8 | < | < | 0.16 | 0.33 | 0.19 | 0.87 | < |
| 9 | 0.45 | 0.30 | 0.77 | 0.33 | 0.54 | 4.02 | < |
| 10 | < | < | 0.13 | 0.15 | 0.14 | 0.84 | < |
| 11 | 0.28 | 0.23 | 0.52 | 0.78 | 0.93 | 5.82 | < |
| 12 | 0.26 | 0.22 | 0.48 | 0.12 | 0.20 | 1.50 | < |
| 13 | 0.28 | 0.17 | 0.47 | 1.33 | 0.63 | 1.57 | < |
| 14 | 0.53 | 0.43 | 0.97 | 3.12 | 1.21 | 3.69 | < |
| 15 | < | < | < | < | 0.17 | 2.04 | < |
| 16 | 0.21 | 0.16 | 0.38 | 0.18 | 0.45 | 1.18 | < |
| 17 | 0.71 | 0.57 | 1.30 | 1.05 | 0.97 | 2.87 | < |
| 18 | 0.23 | 0.20 | 0.44 | 0.22 | 0.32 | 2.05 | < |
| 19 | 0.18 | 0.15 | 0.34 | 0.66 | 0.43 | 5.35 | < |
| 20 | 0.13 | 0.10 | 0.23 | 0.28 | 0.23 | 1.11 | < |
| 21 | 0.21 | 0.11 | 0.34 | 0.81 | 0.55 | 2.44 | < |
| 22 | 0.39 | 0.27 | 0.67 | 1.01 | 0.70 | 5.86 | < |
| 23 | 0.35 | 0.38 | 0.72 | 0.40 | 0.58 | 5.68 | < |
| 24 | 0.48 | 0.36 | 0.86 | 0.31 | 0.44 | 2.66 | < |
| 25 | 0.59 | 0.39 | 1.01 | 0.77 | 0.59 | 3.75 | < |
| 26 | 0.73 | 0.40 | 1.18 | 0.58 | 0.50 | 3.24 | < |
| 27 | 0.42 | 0.23 | 0.69 | 0.33 | 0.35 | 2.83 | < |
| 28 | 0.21 | 0.16 | 0.38 | 0.31 | 0.26 | 1.84 | < |
| 29 | 1.00 | 0.52 | 1.59 | 2.62 | 1.01 | 10.20 | < |
| 30 | 0.14 | < | 0.23 | 1.69 | 0.49 | 1.00 | < |
| 31 | 0.43 | 0.22 | 0.68 | 0.15 | 0.67 | 2.70 | < |
| 32 | 0.20 | 0.15 | 0.35 | 0.15 | 0.34 | 1.69 | < |
| 33 | 0.43 | 0.26 | 0.72 | 1.05 | 0.99 | 1.60 | < |
| 34 | < | < | < | < | 0.22 | 3.45 | < |
| 35 | 0.69 | 0.32 | 1.06 | 0.55 | 0.71 | 2.03 | < |
| LOQ | 0.10 | 0.10 | 0.10 | 0.10 | 0.10 | 0.10 | 0.10 |

# Suspect list urine

| Compound | Formula | |
| --- | --- | --- |
| 2,4,5-T | | C8H5Cl3O3 |
| 2,4,6-trichlorophenoxyacetic acid | C8H5Cl3O3 | |
| 2,4-DB | | C10H10Cl2O3 |
| 2-chloro-1,3-thiazole-5-carboxylic acid | C4H2ClNO2S | |
| 2-diethylamino-6-methyl pyrimidin-4-ol | C9H15N3O | |
| 3,5,6-trichloro-2-pyridinol (TCPy) | C5H2Cl3NO | |
| 3,5-Dichloro-4-hydroxybenzoic acid (tolclophos-methyl) | C7H4Cl2O3 | |
| 4-HSA | C10H12ClNSO6 | |
| 4-Hydroxychlorpropham | C10H12ClNO3 | |
| 5-HBC | C9H9N3O3 | |
| 5-OH-thiabendazole | C10H7N3OS | |
| 6-chloronicotinic acid | C6H4ClNO2 | |
| abamectin/avermectin B1a | C48H72O14 | |
| abamectin/avermectin B1a#pCH2O | C49H74O15 | |
| abamectin/avermectin B1a#pH2 | | C48H74O14 |
| abamectin/avermectin B1a_nCH2 | C47H70O14 | |
| abamectin/avermectin B1a_nCH2#pCH2O | C48H72O15 | |
| abamectin/avermectin B1a_nCH2#pO | | C47H70O15 |
| abamectin/avermectin B1b | C47H70O14 | |
| abamectin/avermectin B1b#pCH2O | C48H72O15 | |
| abamectin/avermectin B1b#pH2 | | C47H72O14 |
| abamectin/avermectin B1b_nCH2 | C46H68O14 | |
| abamectin/avermectin B1b_nCH2#pCH2O | C47H70O15 | |
| abamectin/avermectin B1b_nCH2#pO | | C46H68O15 |
| Acetamiprid | | C10H11ClN4 |
| acetamiprid | C10H11ClN4 | |
| acetamiprid#nC3H2N2 | C7H9N2Cl1 | |
| acetamiprid#nC4H4N2 | C6H7N2Cl1 | |
| acetamiprid#nC6H4ClN | C4H7N3 | |
| acetamiprid#nC7H6ClN | C3H5N3 | |
| acetamiprid#nCH2 | | C9H9N4Cl1 |
| acetamiprid_IC_O | C6H4ClNO2 | |
| acetamiprid_IC_O#pC2H3NO | C8H7N2O3Cl1 | |
| acetamiprid_IM-O | C6H6ClNO | |
| acetamiprid_IM-O#pC2H3N | | C8H9N2O1Cl1 |
| acetamiprid_IM-O#pC3H5N | C9H11N2O1Cl1 | |
| Acetamiprid-N-desmethyl | C9H9ClN4 | |
| Alkyltrimethyl ammonium (ATMAC-10) | C12H27N | |
| Alkyltrimethyl ammonium (ATMAC-12) | C14H31N | |
| Alkyltrimethyl ammonium (ATMAC-14) | C16H35N | |
| Alkyltrimethyl ammonium (ATMAC-16) | C18H39N | |
| Alkyltrimethyl ammonium (ATMAC-18) | C20H43N | |
| Alkyltrimethyl ammonium (ATMAC-20) | C22H47N | |
| Alkyltrimethyl ammonium (ATMAC-8) | C10H23N | |
| allethrin I | C19H26O3 | |
| allethrin I#pO | C19H26O4 | |
| allethrin I#pO2 | C19H26O5 | |
| allethrin I_nC10H14O | C9H12O2 | |
| allethrin I_nC10H14O#pH2O2 | C9H14O4 | |
| allethrin I_nC9H10O | C10H16O2 | |
| allethrin I_nC9H10O#pH2O2 | C10H18O4 | |
| allethrin I_nC9H10O#pO | | C10H16O3 |
| allethrin I_nC9H12_pO | C10H14O4 | |
| allethrin I_nH2_pO2 | C19H24O5 | |
| allethrin I_nH2_pO2#pH2O2 | C19H26O7 | |
| allethrin II | C20H26O5 | |
| allethrin II#pH2O2 | C20H28O7 | |
| allethrin II#pO | | C20H26O6 |
| allethrin II#pO2 | C20H26O7 | |
| allethrin II_nC11H14O3 | C9H12O2 | |
| allethrin II_nC11H14O3#pH2O2 | C9H14O4 | |
| allethrin II_nC9H10O | C11H16O4 | |
| allethrin II_nC9H10O#nCH2 | C10H14O4 | |
| allethrin II_nCH2 | C19H24O5 | |
| allethrin II_nCH2#pH2O2 | C19H26O7 | |
| ametoctradin | | C15H25N5 |
| Ametoctradin metabolite RP32490 | | C13H19N5O2 |
| ametoctradin#nC2H6pO2 | | C13H19N5O2 |
| ametoctradin#nC2H6pO2nH2OpC2H7NSO3 | | C15H24N6O4S1 |
| ametoctradin#nC3H8pO2 | | C12H17N5O2 |
| ametoctradin#nC4H10pO2 | | C11H15N5O2 |
| ametoctradin#nH2pO2 | | C15H23N5O2 |
| ametoctradin#nH2pO2nH2OpC2H7NSO3 | | C17H28N6O4S1 |
| Asulam | | C8H10N2O4S |
| asulam | C8H10N2O4S | |
| asulam#nC2H2O2 | | C6H8N2O2S1 |
| asulam#pC2H2O2 | C10H12N2O6S1 | |
| azithiram | C6H14N4S4 | |
| azithiram#nC3H6N2S2 | C3H8N2S2 | |
| azithiram#nC4H6N2S4 | C2H8N2 | |
| azithiram#nC5H14N4S2 | C1S2 | |
| azithram_dimethylhydrazide_dithiocarbamic acid | C3H8N2S2 | |
| azithram_dimethylhydrazide_dithiocarbamic acid#pCH2 | C4H10N2S2 | |
| azithram_dimethylhydrazide_dithiocarbamic acid#pOCH2 | C4H10N2O1S2 | |
| azithram_dimethylhydrazide_dithiocarbamic acid_nSpOCH2 | C4H10N2OS | |
| azithram_dimethylhydrazide_dithiocarbamic acid_nSpOCH2#pC2H3NO2 | | C6H13N3O3S1 |
| azithram_dimethylhydrazide_dithiocarbamic acid_nSpOCH2#pC4H5NO3 | | C8H15N3O4S1 |
| azithram_dimethylhydrazide_dithiocarbamic acid_nSpOCH2#pO | C4H10N2O2S1 | |
| azithram_dimethylhydrazide_dithiocarbamic acid_nSpOCH2#pO2 | C4H10N2O3S1 | |
| azomate/cuprobam/ferbam/ziram/thiram/tecoram_dimethyldithiocarbamic acid | C3H7NS2 | |
| azomate/cuprobam/ferbam/ziram/thiram/tecoram_dimethyldithiocarbamic acid#nC2H7N | C1S2 | |
| azomate/cuprobam/ferbam/ziram/thiram/tecoram_dimethyldithiocarbamic acid#nCS2 | C2H7N1 | |
| azomate/cuprobam/ferbam/ziram/thiram/tecoram_dimethyldithiocarbamic acid#pCH2 | C4H9N1S2 | |
| azomate/cuprobam/ferbam/ziram/thiram/tecoram_dimethyldithiocarbamic acid#pOCH2 | C4H9N1O1S2 | |
| azomate/cuprobam/ferbam/ziram/thiram/tecoram_dimethyldithiocarbamic acid_nSpOCH2 | C4H9NOS | |
| azomate/cuprobam/ferbam/ziram/thiram/tecoram_dimethyldithiocarbamic acid_nSpOCH2#pO | C4H9N1O2S1 | |
| azomate/cuprobam/ferbam/ziram/thiram/tecoram_dimethyldithiocarbamic acid_nSpOCH2#pO2 | C4H9N1O3S1 | |
| azomate/cuprobam/ferbam/ziram/thiram/tecoram_dimethyldithiocarbamic acid_nSpOCH2#pC4H5NO3 | C8H14N2O4S1 | |
| azomate/cuprobam/ferbam/ziram/thiram/tecoram_dimethyldithiocarbamic acid_nSpOCH2#pC2H3NO2 | C6H12N2O3S1 | |
| azoxystrobin | C22H17N3O5 | |
| azoxystrobin#nC11H10O3 | C11H7N3O2 | |
| azoxystrobin#nC11H5N3O2 | C11H12O3 | |
| azoxystrobin#nC15H12N2O4 | C7H5N1O1 | |
| azoxystrobin#nC7H3N | | C15H14N2O5 |
| azoxystrobin#nCH2 | | C21H15N3O5 |
| azoxystrobin#pC3H5NO2S | C25H22N4O7S1 | |
| azoxystrobin#pC5H7NO3S | | C27H24N4O8S1 |
| azoxystrobin#pO | | C22H17N3O6 |
| azoxystrobin#pS | | C22H17N3O5S1 |
| azoxystrobin#pSCH2 | C23H19N3O5S1 | |
| azoxystrobin#pSCH2O | C23H19N3O6S1 | |
| azoxystrobin#pSO | | C22H17N3O6S1 |
| azoxystrobin_nC2H2O | C20H15N3O4 | |
| azoxystrobin_nC2H2O#nCH2 | C19H13N3O4 | |
| azoxystrobin_nC2H2O#pO | | C20H15N3O5 |
| benomyl_nC5H9N2O | C9H9N3O2 | |
| benomyl_nC5H9N2O#pC3H7NO3S | C12H16N4O5S1 | |
| benomyl_nC5H9N2O#pC5H9NO4S | | C14H18N4O6S1 |
| benomyl_nC5H9N2O#pCH4OS | C10H13N3O3S1 | |
| benomyl_nC5H9N2O#pH2O2 | | C9H11N3O4 |
| benomyl_nC5H9N2O#pH2OS | C9H11N3O3S1 | |
| benomyl_nC5H9N2O#pO | C9H9N3O3 | |
| benomyl_nC5H9N2O#pO2 | C9H9N3O4 | |
| benomyl_nC5H9N2O_56HOBC_Noxide | C9H7N3O5 | |
| Benzalkyl ammonium (BAC-C18) | C27H49N | |
| Benzalkyl ammonium (BAC-C18)#nC7H6 | C20H43N1 | |
| Benzalkyl ammonium (BAC-C6) | C15H25N | |
| Benzalkyl ammonium (BAC-C6)#nC7H6 | C8H19N1 | |
| Benzalkyl ammonium (BAC-C8) | C17H29N | |
| Benzalkyl ammonium (BAC-C8)#nC7H6 | C10H23N1 | |
| Benzalkyl ammonium (BAC-C12) | C21H37N | |
| Benzalkyl ammonium (BAC-C12)#nC7H6 | C14H31N1 | |
| Benzalkyl ammonium (BAC-C14) | C23H41N | |
| Benzalkyl ammonium (BAC-C14)#nC7H6 | C16H35N1 | |
| Benzalkyl ammonium (BAC-C16) | C25H45N | |
| Benzalkyl ammonium (BAC-C16)#nC7H6 | C18H39N1 | |
| Benzalkyl ammonium(BAC-C10) | C19H33N | |
| Benzalkyl ammonium(BAC-C10)#nC7H6 | C12H27N1 | |
| bifenazate | C17H20N2O3 | |
| bifenazate#nC3H6O | C14H14N2O2 | |
| bifenazate#nC4H8N2O2 | C13H12O1 | |
| bifenazate#nC5H10N2O | | C12H10O2 |
| bifenazate#nC5H10N2O2 | C12H10O1 | |
| bifenazate#nCH2 | C16H18N2O3 | |
| bifenazate#pO | | C17H20N2O4 |
| bifenazate_nH2(diazene) | C17H18N2O3 | |
| bifenazate_nH2(diazene)#nC3H6O | | C14H12N2O2 |
| bifenazate_nH2(diazene)#nCH2 | C16H16N2O3 | |
| bifenazate_nH2(diazene)#pO | C17H18N2O4 | |
| bifenthrin | C23H22ClF3O2 | |
| bifenthrin#pO | C23H22O3F3Cl1 | |
| bifenthrin_nC14H12 | C9H10ClF3O2 | |
| bifenthrin_nC14H12#pO | C9H10O3F3Cl1 | |
| bifenthrin_nC9H10F3Cl | C14H12O2 | |
| bifenthrin_nC9H10F3Cl#pC2H3NO | C16H15N1O3 | |
| bifenthrin_nC9H10F3Cl#pC2H5NO2S | C16H17N1O4S1 | |
| bifenthrin_nC9H10F3Cl#pO | | C14H12O3 |
| bifenthrin_nC9H8OF3Cl | C14H14O | |
| bifenthrin_nC9H8OF3Cl#pO | C14H14O2 | |
| bixafen | C18H12Cl2F3N3O | |
| bixafen#pO | C18H12N3O2F3Cl2 | |
| bixafen_nCH2 | C17H10Cl2F3N3O | |
| bixafen_nCH2#pO | C17H10N3O2F3Cl2 | |
| bixafen_nCH2_nF_pOH | C17H11Cl2F2N3O2 | |
| bixafen_nCH2_nF_pOH#pC3H5NO2S | C20H16N4O4F2S1Cl2 | |
| bixafen_nCH2_nF_pOH#pC5H7NO3S | | C22H18N4O5F2S1Cl2 |
| bixafen_nCH2_nF_pOH#pS | C17H11N3O2F2S1Cl2 | |
| bixafen_nCH2_nF_pOH#pSCH2 | C18H13N3O2F2S1Cl2 | |
| bixafen_nCH2_nF_pOH#pSCH2O | C18H13N3O3F2S1Cl2 | |
| bixafen_nCH2_nF_pOH_nCl_pSCH3 | C18H14ClF2N3O2S | |
| bixafen_nF_pOH | C18H13Cl2F2N3O2 | |
| bixafen_nF_pOH#pC3H5NO2S | C21H18N4O4F2S1Cl2 | |
| bixafen_nF_pOH#pC5H7NO3S | | C23H20N4O5F2S1Cl2 |
| bixafen_nF_pOH#pS | C18H13N3O2F2S1Cl2 | |
| bixafen_nF_pOH#pSCH2 | C19H15N3O2F2S1Cl2 | |
| bixafen_nF_pOH#pSCH2O | C19H15N3O3F2S1Cl2 | |
| bixafen_pyrazole_carboxamide | C6H7F2N3O | |
| bixafen_pyrazole_carboxamide#nCH2 | C5H5N3O1F2 | |
| bixafen_pyrazole_carboxylic | C6H6F2N2O2 | |
| bixafen_pyrazole_carboxylic#nCH2 | C5H4N2O2F2 | |
| boscalid | C18H12Cl2N2O | |
| boscalid#pC3H5NO2S | | C21H17N3O3S1Cl2 |
| boscalid#pC3H5NO3S | | C21H17N3O4S1Cl2 |
| boscalid#pC5H7NO3S | | C23H19N3O4S1Cl2 |
| boscalid#pC5H7NO4S | | C23H19N3O5S1Cl2 |
| boscalid#pO | C18H12N2O2Cl2 | |
| boscalid#pO2 | C18H12N2O3Cl2 | |
| boscalid#pO2CH2 | C19H14N2O3Cl2 | |
| boscalid#pS | C18H12N2O1S1Cl2 | |
| boscalid#pSCH2 | C19H14N2O1S1Cl2 | |
| boscalid#pSCH2O | C19H14N2O2S1Cl2 | |
| boscalid#pSO | | C18H12N2O2S1Cl2 |
| boscalid_n510F47 | C6H4ClNO2 | |
| boscalid_nCl_pH | C18H13ClN2O | |
| boscalid_nCl_pH#pC2H2O3 | C20H15N2O4Cl1 | |
| boscalid_nCl_pH#pC2H2O4 | C20H15N2O5Cl1 | |
| boscalid_nCl_pH#pC3H5NO2S | C21H18N3O3S1Cl1 | |
| boscalid_nCl_pH#pC3H5NO3S | | C21H18N3O4S1Cl1 |
| boscalid_nCl_pH#pC5H7NO3S | | C23H20N3O4S1Cl1 |
| boscalid_nCl_pH#pC5H7NO4S | C23H20N3O5S1Cl1 | |
| boscalid_nCl_pH#pO | C18H13N2O2Cl1 | |
| boscalid_nCl_pH#pS | C18H13N2O1S1Cl1 | |
| boscalid_nCl_pH#pSCH2 | C19H15N2O1S1Cl1 | |
| boscalid_nCl_pH#pSCH2O | C19H15N2O2S1Cl1 | |
| boscalid_nCl_pH#pSO | | C18H13N2O2S1Cl1 |
| boscalid_nCl_pH#pSO2 | C18H13N2O3S1Cl1 | |
| boscalid_nCl_pH#pSO3CH2 | C19H15N2O4S1Cl1 | |
| Boscalid-OH (metabolite M510F01) | | C18H12Cl2N2O2 |
| bromoxynil (just conjugation; amide in soil) | | C7H3Br2NO |
| bromoxynil (just conjugation; amide in soil)#pH2O | | C7H5N1O2Br2 |
| bupirimate | C13H24N4O3S | |
| bupirimate#nC2H5NO2S | C11H19N3O1 | |
| bupirimate_nC2H5NO2S | C11H19N3O | |
| bupirimate_nC2H5NO2S#nC2H4 | C9H15N3O1 | |
| bupirimate_nC2H5NO2S#pO | C11H19N3O2 | |
| bupirimate_nC2H5NO2S_nC2H4 | C9H15N3O | |
| bupirimate_nC2H5NO2S_nC2H4#pCH2O | C10H17N3O2 | |
| bupirimate_nC2H5NO2S_nC2H4#pO | | C9H15N3O2 |
| buprofesin_nH2_pO2 | C16H21N3O4S | |
| buprofezin | C16H23N3OS | |
| buprofezin#nC10H16N2S | C6H7N1O1 | |
| buprofezin#nC14H18 | C2H5N3O1S1 | |
| buprofezin#nC6H9NS | | C10H14N2O1 |
| buprofezin#pC2H4O2 | | C18H27N3O3S1 |
| buprofezin#pCH2O2 | | C17H25N3O3S1 |
| buprofezin#pH2O | | C16H25N3O2S1 |
| buprofezin#pO | | C16H23N3O2S1 |
| buprofezin#pO2 | | C16H23N3O3S1 |
| buprofezin_pO | C16H23N3O2S | |
| buprofezin_pO#nC14H18S | C2H5N3O2 | |
| buprofezin_pO#nC6H9NS | C10H14N2O2 | |
| buprofezin_pO#nC8H14N2S | C8H9N1O2 | |
| captan | C9H8Cl3NO2S | |
| captan#pO | C9H8N1O3S1Cl3 | |
| captan_DMS_acid | C4H10O6S4 | |
| captan_DMS_acid#pO | C4H10O7S4 | |
| captan_THPAI | C8H10O4 | |
| captan_THPI/THCY | C8H9NO2 | |
| captan_THPI/THCY#nH4 | C8H5N1O2 | |
| captan_THPI/THCY#pH2O | | C8H11N1O3 |
| captan_THPI/THCY#pH2O2 | C8H11N1O4 | |
| captan_THPI/THCY#pH2O3 | C8H11N1O5 | |
| captan_THPI/THCY#pO | C8H9N1O3 | |
| captan_TTC | C3H5NO2S2 | |
| carbamorph | C8H16N2OS2 | |
| carbamorph_mercapturic | C6H12N2O2S2 | |
| carbamorph_mercapturic#pC2H2O | C8H14N2O3S2 | |
| carbendazim | C9H9N3O2 | |
| carbendazim | C9H9N3O2 | |
| carbendazim#pC3H7NO3S | C12H16N4O5S1 | |
| carbendazim#pC5H9NO4S | | C14H18N4O6S1 |
| carbendazim#pCH4OS | C10H13N3O3S1 | |
| carbendazim#pH2O2 | | C9H11N3O4 |
| carbendazim#pH2OS | C9H11N3O3S1 | |
| carbendazim#pO | C9H9N3O3 | |
| carbendazim#pO2 | C9H9N3O4 | |
| carbendazim_56HOBC_Noxide | C9H7N3O5 | |
| chlorantraniliprole | C18H14BrCl2N5O2 | |
| chlorantraniliprole#nH2O | C18H12N5O1Cl2Br1 | |
| chlorantraniliprole_nCH2 | C17H12BrCl2N5O2 | |
| chlorantraniliprole_nCH2#nH2O | C17H10N5O1Cl2Br1 | |
| chlorantraniliprole_nCH2#pCO2 | C18H12N5O4Cl2Br1 | |
| chlorantraniliprole_pO | C18H14BrCl2N5O3 | |
| chlorantraniliprole_pO#nC9H5BrClN3 | C9H9N2O3Cl1 | |
| chlorantraniliprole_pO#nC9H9ClN2O | | C9H5N3O2Cl1Br1 |
| chlorantraniliprole_pO#nCH2 | C17H12N5O3Cl2Br1 | |
| chlorantraniliprole_pO#nCH3N | C17H11N4O3Cl2Br1 | |
| chlorantraniliprole_pO#nCH4O | C17H10N5O2Cl2Br1 | |
| chlorantraniliprole_pO#nCH6 | | C17H8N5O3Cl2Br1 |
| chlorantraniliprole_pO#nH2O | | C18H12N5O2Cl2Br1 |
| chlorantraniliprole_pO#nH4 | C18H10N5O3Cl2Br1 | |
| chlorantraniliprole_pO#pO | C18H14N5O4Cl2Br1 | |
| chlorazifop | C14H11Cl2NO4 | |
| chlorazifop#nC3H4O2 | | C11H7N1O2Cl2 |
| chlorazifop#nC9H8O3 | | C5H3N1O1Cl2 |
| chlorazifop-propargyl | | C17H13Cl2NO4 |
| chlorazifop-propargyl#nC3H2 | | C14H11N1O4Cl2 |
| chlorbufam | | C11H10ClNO2 |
| chlorbufam#pO | C11H10N1O3Cl1 | |
| chloridazon | C10H8ClN3O | |
| chloridazon#pO | C10H8N3O2Cl1 | |
| chloridazon#pO2 | C10H8N3O3Cl1 | |
| chloridazon_nClpH | C10H9N3O | |
| chloridazon_nClpH#pO | C10H9N3O2 | |
| chlorocresol | | C7H7ClO |
| chlorophenol | | C6H5ClO |
| chlorothalonil | C8Cl4N2 | |
| chlorothalonil#pH2O | C8H2N2O1Cl4 | |
| chlorothalonil_nClNpH4O3 | C8H4Cl3NO3 | |
| Chlorothalonil_nClpO | | C8Cl3N2O |
| chlorothalonil_nClpOH | C8HCl3N2O | |
| chlorothalonil_nClpOH#pH2O | C8H3N2O2Cl3 | |
| chloroxuron | C15H15ClN2O2 | |
| chloroxuron#nC2H4 | C13H11N2O2Cl1 | |
| chloroxuron#nC3H5NO | C12H10N1O1Cl1 | |
| chloroxuron#nCH2 | | C14H13N2O2Cl1 |
| chloroxylenol | | C8H9ClO |
| chloroxynil (just conjugation; amide in soil) | | C7H3Cl2NO |
| chloroxynil (just conjugation; amide in soil)#pH2O | | C7H5N1O2Cl2 |
| chlorpropham | C10H12ClNO2 | |
| chlorpropham#nC4H6O | | C6H6N1O1Cl1 |
| chlorpropham#nC4H6O2 | | C6H6N1Cl1 |
| chlorpropham#nH2pO2 | | C10H10N1O4Cl1 |
| chlorpropham#pO | C10H12N1O3Cl1 | |
| chlorpropham#pO2 | C10H12N1O4Cl1 | |
| chlorpyrifos | C9H11Cl3NO3PS | |
| chlorpyrifos#nC2H4 | C7H7N1O3P1S1Cl3 | |
| chlorpyrifos#nC4H9O2PS | | C5H2N1O1Cl3 |
| chlorpyrifos#nC5Cl3N | | C4H11O3P1S1 |
| chlorpyrifos_nSpO | C9H11Cl3NO4P | |
| chlorpyrifos_nSpO#nC2H4 | C7H7N1O4P1Cl3 | |
| chlorpyrifos_nSpO#nC5Cl3N | C4H11O4P1 | |
| chlorpyrifos_TCPy_nClpH | C5Cl2H2NO | |
| chlorpyrifos_TCPy_nClpH#pC3H5NO2S | | C8H7N2O3S1Cl2 |
| chlorpyrifos_TCPy_nClpH#pC5H7NO3S | C10H9N2O4S1Cl2 | |
| chlorpyrifos_TCPy_nClpH#pS | C5H2N1O1S1Cl2 | |
| chlorpyrifos_TCPy_nClpH#pSCH2 | C6H4N1O1S1Cl2 | |
| chlorpyrifos_TCPy_nClpH#pSCH2O | C6H4N1O2S1Cl2 | |
| chlorpyrifos-methyl | | C7H7Cl3NO3PS |
| chlorpyrifos-methyl#nC2H5O2PS | | C5H2N1O1Cl3 |
| chlorpyrifos-methyl#nC5Cl3N | | C2H7O3P1S1 |
| chlorpyrifos-methyl#nCH2 | | C6H5N1O3P1S1Cl3 |
| chlorpyrifos-methyl_nSpO | C7H7Cl3NO4P | |
| chlorpyrifos-methyl_nSpO#nC5Cl3N | | C2H7O4P1 |
| chlorpyrifos-methyl_nSpO#nCH2 | C6H5N1O4P1Cl3 | |
| cinerin I | C20H28O3 | |
| cinerin I#pO | C20H28O4 | |
| cinerin I#pO2 | C20H28O5 | |
| cinerin I_nC10H12O | C10H16O2 | |
| cinerin I_nC10H12O#pH2O2 | C10H18O4 | |
| cinerin I_nC10H12O#pO | | C10H16O3 |
| cinerin I_nC10H14_pO | C10H14O4 | |
| cinerin I_nC10H14O | C10H14O2 | |
| cinerin I_nC10H14O#pH2O2 | C10H16O4 | |
| cinerin I_nH2_pO2 | C20H26O5 | |
| cinerin I_nH2_pO2#pH2O2 | C20H28O7 | |
| cinerin II | C21H28O5 | |
| cinerin II#pH2O2 | C21H30O7 | |
| cinerin II#pO | | C21H28O6 |
| cinerin II#pO2 | C21H28O7 | |
| cinerin II_C11H14O3 | C10H14O2 | |
| cinerin II_C11H14O3#pH2O2 | C10H16O4 | |
| cinerin II_nC10H12O | C11H16O4 | |
| cinerin II_nC10H12O#nCH2 | C10H14O4 | |
| cinerin II_nCH2 | C20H26O5 | |
| cinerin II_nCH2#pH2O2 | C20H28O7 | |
| clodinafop | | C14H11ClFNO4 |
| clodinafop#nC3H4O2 | | C11H7N1O2F1Cl1 |
| clodinafop#nC9H8O3 | | C5H3N1O1F1Cl1 |
| clodinafop-propargyl | | C17H13ClFNO4 |
| clodinafop-propargyl#nC3H2 | | C14H11N1O4F1Cl1 |
| clomazone | | C12H14ClNO2 |
| clomazone#nC5NH9 | | C7H5O2Cl1 |
| clomazone#pH2 | | C12H16N1O2Cl1 |
| clomazone#pH2pO2 | | C12H16N1O4Cl1 |
| clomazone#pH2pO3 | | C12H16N1O5Cl1 |
| clomazone#pO | | C12H14N1O3Cl1 |
| clomazone#pO2 | | C12H14N1O4Cl1 |
| clomazone#pOnH2 | | C12H12N1O3Cl1 |
| clomazone#pOnH2nCO2 | | C11H12N1O1Cl1 |
| clomazone#pOpH2 | | C12H16N1O3Cl1 |
| Cloprop | | C9H9ClO3 |
| Cloprop#pO | | C9H9O4Cl1 |
| clopyralid | | C6H3Cl2NO2 |
| CTMA | | C10H12ClNO3 |
| cyfluthrin | C22H18Cl2FNO3 | |
| cyfluthrin_nC14H8FNO | C8H10Cl2O2 | |
| cyfluthrin_nC14H8FNO#pO | C8H10O3Cl2 | |
| cyfluthrin_nC8H8Cl2O2 | C14H10FNO | |
| cyfluthrin_nC8H8Cl2O2#pO | C14H10N1O2F1 | |
| cyfluthrin_nC9H9Cl2N | C13H9FO3 | |
| cyfluthrin_nC9H9Cl2N#pC2H3NO | C15H12N1O4F1 | |
| cyfluthrin_nC9H9Cl2N#pC2H5NO2S | C15H14N1O5F1S1 | |
| cyfluthrin_nC9H9Cl2N#pO | | C13H9O4F1 |
| cyhalofop | | C16H12FNO4 |
| cyhalofop#nC3H4O2 | | C13H8N1O2F1 |
| cyhalofop#nC9H8O3 | | C7H4N1O1F1 |
| cyhalofop-butyl | | C20H20FNO4 |
| cyhalofop-butyl#nC4H8 | | C16H12N1O4F1 |
| cyhalothrin | C23H19ClF3NO3 | |
| cyhalothrin#pO | C23H19N1O4F3Cl1 | |
| cyhalothrin_nC10H9ClF3N | C13H10O3 | |
| cyhalothrin_nC10H9ClF3N#pC2H3NO | C15H13N1O4 | |
| cyhalothrin_nC10H9ClF3N#pC2H5NO2S | C15H15N1O5S1 | |
| cyhalothrin_nC10H9ClF3N#pO | | C13H10O4 |
| cyhalothrin_nC14H9NO | C9H10ClF3O2 | |
| cyhalothrin_nC14H9NO#pO | C9H10O3F3Cl1 | |
| cyhalothrin_nC9H8ClF3O | C14H11NO2 | |
| cyhalothrin_nC9H8ClF3O#pO | C14H11N1O3 | |
| cypermethrin | C22H19Cl2NO3 | |
| cypermethrin#pO | C22H19N1O4Cl2 | |
| cypermethrin_nC14H9NO | C8H10Cl2O2 | |
| cypermethrin_nC14H9NO#pO | C8H10O3Cl2 | |
| cypermethrin_nC8H8Cl2O | C14H11NO2 | |
| cypermethrin_nC8H8Cl2O#pO | C14H11N1O3 | |
| cypermethrin_nC9H9Cl2N | C13H10O3 | |
| cypermethrin_nC9H9Cl2N#pC2H3NO | C15H13N1O4 | |
| cypermethrin_nC9H9Cl2N#pC2H5NO2S | C15H15N1O5S1 | |
| cypermethrin_nC9H9Cl2N#pO | | C13H10O4 |
| cyphenothrin | C24H25NO3 | |
| cyphenothrin#pO | C24H25N1O4 | |
| cyphenothrin_nC10H14O | C14H11NO2 | |
| cyphenothrin_nC10H14O#pO | C14H11N1O3 | |
| cyphenothrin_nC11H15N | C13H10O3 | |
| cyphenothrin_nC11H15N#pC2H3NO | C15H13N1O4 | |
| cyphenothrin_nC11H15N#pC2H5NO2S | C15H15N1O5S1 | |
| cyphenothrin_nC11H15N#pO | | C13H10O4 |
| cyphenothrin_nC14H9NO | C10H16O2 | |
| cyphenothrin_nC14H9NO#pO | C10H16O3 | |
| cyprodinil | C14H15N3 | |
| cyprodinil#nC6H4 | | C8H11N3 |
| cyprodinil#pO | C14H15N3O1 | |
| cyprodinil#pO2 | C14H15N3O2 | |
| cyprodinil#pO3 | C14H15N3O3 | |
| cyprodinil_nC7H6 | C7H9N3 | |
| cyprodinil_nC7H6#pO | C7H9N3O1 | |
| D, 2,4- | C8H6Cl2O3 | |
| D, 2,4-#nC2H2O2 | C6H4O1Cl2 | |
| D, 2,4-#nCO2 | | C7H6O1Cl2 |
| D, 2,4-#pC2H3NO | C10H9N1O4Cl2 | |
| D, 2,4-#pC2H5NO2S | C10H11N1O5S1Cl2 | |
| D, 2,4-_nClpH | C8H7ClO3 | |
| DADMAC-10-10 | C22H47N | |
| DADMAC-10-12 | C24H51N | |
| DADMAC-10-14 | C26H55N | |
| DADMAC-10-16 | C28H59N | |
| DADMAC-10-18 | C30H63N | |
| DADMAC-12-12 | C26H55N | |
| DADMAC-12-14 | C28H59N | |
| DADMAC-12-16 | C30H63N | |
| DADMAC-12-18 | C32H67N | |
| DADMAC-14-14 | C30H63N | |
| DADMAC-14-16 | C32H67N | |
| DADMAC-14-18 | C34H71N | |
| DADMAC-16-16 | C34H71N | |
| DADMAC-16-18 | C36H75N | |
| DADMAC-18-18 | C38H79N | |
| DADMAC-6-10 | C18H39N | |
| DADMAC-6-12 | C20H43N | |
| DADMAC-6-14 | C22H47N | |
| DADMAC-6-16 | C24H51N | |
| DADMAC-6-18 | C26H55N | |
| DADMAC-6-6 | C14H31N | |
| DADMAC-6-8 | C16H35N | |
| DADMAC-8-10 | C20H43N | |
| DADMAC-8-12 | C22H47N | |
| DADMAC-8-14 | C24H51N | |
| DADMAC-8-16 | C26H55N | |
| DADMAC-8-18 | C28H59N | |
| DADMAC-8-8 | C18H39N | |
| dazomet | C5H10N2S2 | |
| dazomet_metham_methylisothiocyanate | C2H3NS | |
| dazomet_metham_methylisothiocyanate#pC3H7NO2S | C5H10N2O2S2 | |
| dazomet_metham_methylisothiocyanate#pC5H9NO3S | | C7H12N2O3S2 |
| deltamethrin | C22H19Br2NO3 | |
| deltamethrin#pO | C22H19N1O4Br2 | |
| deltamethrin_nC14H9NO | C8H10Br2O2 | |
| deltamethrin_nC14H9NO#pO | C8H10O3Br2 | |
| deltamethrin_nC8H8Br2O | C14H11NO2 | |
| deltamethrin_nC8H8Br2O#pO | C14H11N1O3 | |
| deltamethrin_nC9H9Br2N | C13H10O3 | |
| deltamethrin_nC9H9Br2N#pC2H3NO | C15H13N1O4 | |
| deltamethrin_nC9H9Br2N#pC2H5NO2S | C15H15N1O5S1 | |
| deltamethrin_nC9H9Br2N#pO | | C13H10O4 |
| desamino-metamitron | | C10H9N3O |
| dicamba | | C8H6Cl2O3 |
| dichlorophenol | | C6H4Cl2O |
| dichromate | | C9H9Cl2NO2 |
| dichromate#nC2H3NO | | C7H6O1Cl2 |
| dichromate#nC2H3NOnH2pO2 | | C7H4O3Cl2 |
| dichromate#nC2H3NOnH2pO2pC2H3NO | | C9H7N1O4Cl2 |
| diclobenil (amide in soil) | | C7H3Cl2N |
| diclobenil (amide in soil)#pH2O | | C7H5N1O1Cl2 |
| diclofop | | C15H12Cl2O4 |
| diclofop#nC3H4O2 | | C12H8O2Cl2 |
| diclofop#nC9H8O3 | | C6H4O1Cl2 |
| diclofop-methyl | | C16H14Cl2O4 |
| diclofop-methyl | | C16H14Cl2O4 |
| diclofop-methyl#nC4H6O2 | | C12H8O2Cl2 |
| diclofop-methyl#nCH2 | | C15H12O4Cl2 |
| diclofop-methyl#nCH2 | | C15H12O4Cl2 |
| diclofop-methyl#nCH2pO | | C15H12O5Cl2 |
| diethyltoluamide (DEET) | C12H17NO | |
| diethyltoluamide (DEET)#nC2H4 | C10H13N1O1 | |
| diethyltoluamide (DEET)#nC4H8 | C8H9N1O1 | |
| diethyltoluamide (DEET)_carboxy | C12H15NO3 | |
| diethyltoluamide (DEET)_carboxy#nC2H4 | C10H11N1O3 | |
| diethyltoluamide (DEET)_carboxy#nC4H8 | C8H7N1O3 | |
| diethyltoluamide (DEET)_pO | C12H17NO2 | |
| diethyltoluamide (DEET)_pO#nC2H4 | C10H13N1O2 | |
| diethyltoluamide (DEET)_pO#nC4H8 | C8H9N1O2 | |
| Difenconazole metabolite CGA-205374 | | C16H11Cl2N3O2 |
| difenoconazole | C19H17Cl2N3O3 | |
| difenoconazole#nC3H4O | C16H13N3O2Cl2 | |
| difenoconazole#pO | C19H17N3O4Cl2 | |
| difenoconazole_nC3H4O | C16H13Cl2N3O2 | |
| difenoconazole_nC3H4O#nH2 | C16H11N3O2Cl2 | |
| difenoconazole_nC3H4O#pO | | C16H13N3O3Cl2 |
| difenoconazole_nC3H4O_carboxy | C14H10Cl2O4 | |
| difenoconazole_nC3H4O_carboxy#pC2H3NO | C16H13N1O5Cl2 | |
| dimethenamide-P | C12H18ClNO2S | |
| dimethenamide-P_M30 | C15H23NO6S2 | |
| dimethenamide-P_M8 | C11H13NO2S | |
| dimethenamide-P_M8#pH2 | | C11H15N1O2S1 |
| dimethenamide-P_M8#pH2O | C11H15N1O3S1 | |
| dimethenamide-P_M8#pH3Cl | C11H16N1O2S1Cl1 | |
| dimethenamide-P_nClpH | C12H19NO2S | |
| dimethenamide-P_nClpH#nCH2 | C11H17N1O2S1 | |
| dimethenamide-P_nClpH#pO | | C12H19N1O3S1 |
| dimethenamide-P_nHCl_pNacCys | C17H26N2O5S2 | |
| dimethenamide-P_nHCl_pNacCys#nC2H2O | C15H24N2O4S2 | |
| dimethenamide-P_nHCl_pNacCys#nC4H5NO | C13H21N1O4S2 | |
| dimethenamide-P_nHCl_pNacCys#nC4H5NO2 | C13H21N1O3S2 | |
| dimethenamide-P_nHCl_pNacCys#nC4H7NO | C13H19N1O4S2 | |
| dimethenamide-P_nHCl_pNacCys#nC5H7NO | C12H19N1O4S2 | |
| dimethenamide-P_nHCl_pNacCys#nC5H7NO2 | C12H19N1O3S2 | |
| dimethenamide-P_nHCl_pNacCys#nC5H7NO3 | C12H19N1O2S2 | |
| dimethenamide-P_nHCl_pNacCys#nC5H9N | | C12H17N1O5S2 |
| dimethenamide-P_nHCl_pNacCys#nC6H11NO3 | C11H15N1O2S2 | |
| dimethenamide-P_nHCl_pNacCys#nC6H9NO3 | C11H17N1O2S2 | |
| dimethenamide-P_nHCl_pNacCys#nNC2H3 | C15H23N1O5S2 | |
| dimethenamide-P_pO | C12H18ClNO3S | |
| dimethenamide-P_pO#nHCl | C12H17N1O3S1 | |
| dimethomorph | C21H22ClNO4 | |
| dimethomorph_nCH2 | C20H20ClNO4 | |
| dimethomorph_nCH2_pH2O | C20H22ClNO5 | |
| dimethomorph_nCH2_pH2O#nC2H4O | C18H18N1O4Cl1 | |
| dimethomorph_nCH2_pH2O#nC2H6 | | C18H16N1O5Cl1 |
| dimethomorph_nCH2_pH2O#nC4H8O2 | C16H14N1O3Cl1 | |
| dimethomorph_nCH2_pH2O#nC4H9NO | C16H13O4Cl1 | |
| dimethomorph_nCH2_pH2O#nH4 | C20H18N1O5Cl1 | |
| dimethomorph_pH2O | C21H24ClNO5 | |
| dimethomorph_pH2O#nC2H4O | C19H20N1O4Cl1 | |
| dimethomorph_pH2O#nC2H6 | | C19H18N1O5Cl1 |
| dimethomorph_pH2O#nC4H8O2 | C17H16N1O3Cl1 | |
| dimethomorph_pH2O#nC4H9NO | C17H15O4Cl1 | |
| dimethomorph_pH2O#nH4 | C21H20N1O5Cl1 | |
| disulfiram | C10H20N2S4 | |
| disulfiram#nC5H9NS2 | C5H11N1S2 | |
| disulfiram#nC6H9NS4 | C4H11N1 | |
| disulfiram#nC9H20N2S2 | C1S2 | |
| disulfiram_Diethyldithiocarbamic acid | C5H11NS2 | |
| disulfiram_Diethyldithiocarbamic acid#pCH2 | C6H13N1S2 | |
| disulfiram_Diethyldithiocarbamic acid#pOCH2 | C6H13N1O1S2 | |
| disulfiram_Diethyldithiocarbamic acid_nSpOCH2 | C6H13NOS | |
| disulfiram_Diethyldithiocarbamic acid_nSpOCH2#pC2H3NO2 | | C8H16N2O3S1 |
| disulfiram_Diethyldithiocarbamic acid_nSpOCH2#pC4H5NO3 | | C10H18N2O4S1 |
| disulfiram_Diethyldithiocarbamic acid_nSpOCH2#pO | C6H13N1O2S1 | |
| disulfiram_Diethyldithiocarbamic acid_nSpOCH2#pO2 | C6H13N1O3S1 | |
| ethephon | C2H6ClO3P | |
| ethephon_nClpO | C2H7O4P | |
| ethirimol | C11H19N3O | |
| ethirimol#nC2H4 | C9H15N3O1 | |
| ethirimol#pO | C11H19N3O2 | |
| ethirimol_nC2H4 | C9H15N3O | |
| ethirimol_nC2H4#pCH4O | C10H19N3O2 | |
| ethirimol_nC2H4#pO | C9H15N3O2 | |
| Ethyl_4-chloroindole-3-acetic acid | | C12H12ClNO2 |
| Ethyl_4-chloroindole-3-acetic acid#nC2H4 | | C10H8N1O2Cl1 |
| etofenprox | C25H28O3 | |
| etofenprox#nC2H4 | C23H24O3 | |
| etofenprox#pO | | C25H28O4 |
| etofenprox_nH2pO | C25H26O4 | |
| etofenprox_nH2pO#nC12H16 | C13H10O4 | |
| etofenprox_nH2pO#nC12H16O | C13H10O3 | |
| etofenprox_nH2pO#nC2H4 | | C23H22O4 |
| etofenprox_nH2pO#pO | | C25H26O5 |
| fenbuconazole | C19H17ClN4 | |
| fenbuconazole#pH2O2 | C19H19N4O2Cl1 | |
| fenbuconazole#pH2O3 | C19H19N4O3Cl1 | |
| fenbuconazole#pO | | C19H17N4O1Cl1 |
| fenbuconazole_nH2pO2 | C19H15ClN4O2 | |
| fenbuconazole_nH2pO2#nCHN | C18H14N3O2Cl1 | |
| fenbuconazole_nH2pO2#nO | | C19H15N4O1Cl1 |
| fenbuconazole_nHNpO2 | C19H16ClN3O2 | |
| fenbuconazole_nHNpO2#pO | C19H16N3O3Cl1 | |
| fenbuconazole_RH-7968 | C17H16ClNO | |
| fenhexamid | C14H17Cl2NO2 | |
| fenhexamid#nHCl | C14H16N1O2Cl1 | |
| fenhexamid#pCH2 | | C15H19N1O2Cl2 |
| fenhexamid#pO | | C14H17N1O3Cl2 |
| fenoxaprop | | C16H12ClNO5 |
| fenoxaprop#nC9H8O3 | | C7H4N1O2Cl1 |
| fenoxaprop#nC9H8O3nOpS | | C7H4N1O1S1Cl1 |
| fenoxaprop#nC9H8O3pO | | C7H4N1O3Cl1 |
| fenoxaprop#nC9H8O4pC5H7NO3S | | C12H11N2O4S1Cl1 |
| fenoxaprop-ethyl | | C18H16ClNO5 |
| fenoxaprop-ethyl#nC2H4 | | C16H12N1O5Cl1 |
| fenoxycarb | C17H19NO4 | |
| fenoxycarb#pCH2O2 | | C18H21N1O6 |
| fenoxycarb#pCH2O3 | | C18H21N1O7 |
| fenoxycarb#pO | C17H19N1O5 | |
| fenoxycarb#pO2 | C17H19N1O6 | |
| fenoxycarb#pO3 | C17H19N1O7 | |
| fenoxycarb_nC3H7NpO | C14H12O5 | |
| fenoxycarb_nC3H7NpO#pCH2O | C15H14O6 | |
| fenoxycarb_nC5N9NO2 | C12H10O2 | |
| fenoxycarb_nC5N9NO2#pCH2O2 | C13H12O4 | |
| fenoxycarb_nC5N9NO2#pO | | C12H10O3 |
| fenoxycarb_nC6H4 | C11H15NO4 | |
| fenoxycarb_nC6H4#pCH2O | C12H17N1O5 | |
| fenoxycarb_nC6H4#pO | C11H15N1O5 | |
| fenoxycarb_nCH4pO2 | C16H15NO6 | |
| fenoxycarb_nCH4pO2#nC2O4 | | C14H15N1O2 |
| fenoxycarb_nCH4pO2#nC6H4O | C10H11N1O5 | |
| fenoxycarb_nCH4pO2#nO | C16H15N1O5 | |
| fenoxycarb_nCH4pO2#pCH2O | C17H17N1O7 | |
| fenoxycarb_nCH4pO2#pO | C16H15N1O7 | |
| fenpropathrin | C22H23NO3 | |
| fenpropathrin#pO | C22H23N1O4 | |
| fenpropathrin_C9H13N | C13H10O3 | |
| fenpropathrin_C9H13N#pC2H3NO | C15H13N1O4 | |
| fenpropathrin_C9H13N#pC2H5NO2S | C15H15N1O5S1 | |
| fenpropathrin_C9H13N#pO | | C13H10O4 |
| fenpropathrin_nC14H9NO | C8H14O2 | |
| fenpropathrin_nC14H9NO#pO | C8H14O3 | |
| fenpropathrin_nC8H12O | C14H11NO2 | |
| fenpropathrin_nC8H12O#pO | C14H11N1O3 | |
| fenpropidin | C19H31N | |
| fenpropidin#pO | C19H31N1O1 | |
| fenpropidin#pO2 | C19H31N1O2 | |
| fenpropidin_nH2pO4 | C19H29NO4 | |
| fenpropidin_nH2pO4#nC2H2O2 | C17H27N1O2 | |
| fenpropidin_nH2pO4#nC5H6O3 | C14H23N1O1 | |
| fenpropidin_nH2pO4#nC5H8O2 | | C14H21N1O2 |
| fenpropidin_nH2pO4#nC8H15NO | C11H14O3 | |
| fenpropidin_nH2pO4#nO | | C19H29N1O3 |
| fenpropidin_nH2pO4#nO2 | | C19H29N1O2 |
| fenpropimorph | C20H33NO | |
| Fenpropimorph acid metabolite | C20H31NO3 | |
| fenpropimorph#nC14H20 | C6H13N1O1 | |
| fenpropimorph#pO | | C20H33N1O2 |
| fenpropimorph_nH2pO2 | C20H31NO3 | |
| fenpropimorph_nH2pO2#nC14H18O | | C6H13N1O2 |
| fenpropimorph_nH2pO2#pH2O | | C20H33N1O4 |
| fenpropimorph_nH2pO2#pO | C20H31N1O4 | |
| fenpropimorph_nH2pO2#pO2 | C20H31N1O5 | |
| fenpropimorph_nH2pO2_nC3H4 | C17H27NO3 | |
| fenpropimorph_nH2pO2_nC3H4#pO | C17H27N1O4 | |
| fenpropimorph_nH4pO4 | C20H29NO5 | |
| fenpropimorph_nH4pO4#nC6H11NO | C14H18O4 | |
| fenpropimorph_nH4pO4#nC6H9NO3 | | C14H20O2 |
| fenpropimorph_nH4pO4#nC9H15NO2 | C11H14O3 | |
| fenpropimorph_nH4pO4#nC9H17NO | C11H12O4 | |
| fenvalerate | C25H22ClNO3 | |
| fenvalerate#pO | C25H22N1O4Cl1 | |
| fenvalerate_C11H11ClO | C14H11NO2 | |
| fenvalerate_C11H11ClO#pO | C14H11N1O3 | |
| fenvalerate_nC12H12ClN | C13H10O3 | |
| fenvalerate_nC12H12ClN#pC2H3NO | C15H13N1O4 | |
| fenvalerate_nC12H12ClN#pC2H5NO2S | C15H15N1O5S1 | |
| fenvalerate_nC12H12ClN#pO | | C13H10O4 |
| fenvalerate_nC14H9NO | C11H13ClO2 | |
| fenvalerate_nC14H9NO#pO | C11H13O3Cl1 | |
| fipronil | C12H4Cl2F6N4OS | |
| fipronil#nC3F3NOS | C9H4N3F3Cl2 | |
| fipronil#nO | C12H4N4F6S1Cl2 | |
| fipronil#pH2O | C12H6N4O2F6S1Cl2 | |
| fipronil#pO | C12H4N4O2F6S1Cl2 | |
| fipronil_nCClF3S_pH2 | C11H6ClF3N4O | |
| fipronil_pHO2_nSOCF3 | C11H5Cl2F3N4O2 | |
| fipronil_pHO2_nSOCF3#nCHN | | C10H4N3O2F3Cl2 |
| fipronil_pHO2_nSOCF3#nH2O | | C11H3N4O1F3Cl2 |
| fipronil_pHO2_nSOCF3#nO | C11H5N4O1F3Cl2 | |
| fipronil_pHO2_nSOCF3#nO2 | C11H5N4F3Cl2 | |
| Flonicamid | C9H6F3N3O | |
| flonicamid | C9H6F3N3O | |
| flonicamid#pH2O | C9H8N3O2F3 | |
| flonicamid#pO | | C9H6N3O2F3 |
| flonicamid_nC2HN | C7H5F3N2O | |
| flonicamid_nC2HN#pO | C7H5N2O2F3 | |
| flonicamid_pO_nC2H2N2 | C7H4F3NO2 | |
| flonicamid_pO_nC2H2N2#pC2H3NO | C9H7N2O3F3 | |
| fluazifop | | C15H12F3NO4 |
| fluazifop#nC3H4O2 | | C12H8N1O2F3 |
| fluazifop#nC9H8O3 | | C6H4N1O1F3 |
| fluazifop-butyl | | C19H20F3NO4 |
| fluazifop-butyl#nC4H8 | | C15H12N1O4F3 |
| fluazifop-methyl | | C16H14F3NO4 |
| fluazifop-methyl#nCH2 | | C15H12N1O4F3 |
| fludioxonil | C12H6F2N2O2 | |
| fludioxonil#pC12H2F2N2O4 | C24H8N4O6F4 | |
| fludioxonil#pO | | C12H6N2O3F2 |
| fludioxonil_CGA265378 | C12H4F2N2O4 | |
| fludioxonil_CGA344624 | C9H5F2NO4 | |
| fludioxonil_CGA344624#nCHN | C8H4O4F2 | |
| fludioxonil_CGA344624#pC3H3NO | C12H8N2O5F2 | |
| flumethrin | C28H22Cl2FNO3 | |
| flumethrin#pO | C28H22N1O4F1Cl2 | |
| flumethrin_nC14H12Cl2O | C14H10FNO2 | |
| flumethrin_nC14H12Cl2O#pO | C14H10N1O3F1 | |
| flumethrin_nC14H8FNO | C14H14Cl2O2 | |
| flumethrin_nC14H8FNO#pO | C14H14O3Cl2 | |
| flumethrin_nC15H13Cl2N | C13H9FO3 | |
| flumethrin_nC15H13Cl2N#pC2H3NO | C15H12N1O4F1 | |
| flumethrin_nC15H13Cl2N#pC2H5NO2S | C15H14N1O5F1S1 | |
| flumethrin_nC15H13Cl2N#pO | | C13H9O4F1 |
| flumezin (no metab info: used linuron as example) | | C11H9F3N2O3 |
| flumezin (no metab info: used linuron as example)#nC2O2 | | C9H9N2O1F3 |
| flumezin (no metab info: used linuron as example)#nC2O2pO | | C9H9N2O2F3 |
| flumezin (no metab info: used linuron as example)#nC3H2O2 | | C8H7N2O1F3 |
| flumezin (no metab info: used linuron as example)#nC3H2O2pO | | C8H7N2O2F3 |
| flumezin (no metab info: used linuron as example)#nCH2 | | C10H7N2O3F3 |
| flumezin (no metab info: used linuron as example)#nCH2pO | | C10H7N2O4F3 |
| fluopicolide | C14H8Cl3F3N2O | |
| fluopicolide#pH2O2 | | C14H10N2O3F3Cl3 |
| fluopicolide#pO | C14H8N2O2F3Cl3 | |
| fluopicolide#pO2 | C14H8N2O3F3Cl3 | |
| fluopicolide_nC7H3Cl2NO | C7H5F3ClN | |
| fluopicolide_nC7H3Cl2NO#pC3H5NO2S | C10H10N2O2F3S1Cl1 | |
| fluopicolide_nC7H3Cl2NO#pC5H7NO3S | | C12H12N2O3F3S1Cl1 |
| fluopicolide_nC7H3Cl2NO#pO | | C7H5N1O1F3Cl1 |
| fluopicolide_nC7H3Cl2NO#pS | | C7H5N1F3S1Cl1 |
| fluopicolide_nC7H3Cl2NO#pSCH2 | C8H7N1F3S1Cl1 | |
| fluopicolide_nC7H3Cl2NO#pSCH2O | C8H7N1O1F3S1Cl1 | |
| fluopicolide_nC7H3ClF3N | C7H5Cl2NO | |
| fluopicolide_nC7H3ClF3N#pC2H2O | C9H7N1O2Cl2 | |
| fluopicolide_nC8H5Cl2NO | C6H3F3ClN | |
| fluopicolide_nC8H5Cl2NO#pC3H5NO2S | | C9H8N2O2F3S1Cl1 |
| fluopicolide_nC8H5Cl2NO#pC5H7NO3S | C11H10N2O3F3S1Cl1 | |
| fluopicolide_nC8H5Cl2NO#pS | C6H3N1F3S1Cl1 | |
| fluopicolide_nC8H5Cl2NO#pSCH2 | C7H5N1F3S1Cl1 | |
| fluopicolide_nC8H5Cl2NO#pSCH2O | C7H5N1O1F3S1Cl1 | |
| fluopicolide_pO_nC7H2Cl2F3N | C7H6ClNO2 | |
| fluopicolide_pO_nC7H2Cl2F3N#pC3H5NO2S | C10H11N2O4S1Cl1 | |
| fluopicolide_pO_nC7H2Cl2F3N#pC5H7NO3S | | C12H13N2O5S1Cl1 |
| fluopicolide_pO_nC7H2Cl2F3N#pS | | C7H6N1O2S1Cl1 |
| fluopicolide_pO_nC7H2Cl2F3N#pSCH2 | C8H8N1O2S1Cl1 | |
| fluopicolide_pO_nC7H2Cl2F3N#pSCH2O | C8H8N1O3S1Cl1 | |
| fluopicolide_pO_nC7H5Cl2N | C7H3ClF3NO2 | |
| fluopyram | C16H11ClF6N2O | |
| fluopyram#nH2 | C16H9N2O1F6Cl1 | |
| fluopyram#pCH2O3 | C17H13N2O4F6Cl1 | |
| fluopyram#pO2 | | C16H11N2O3F6Cl1 |
| fluopyram#pO3 | C16H11N2O4F6Cl1 | |
| fluopyram_nC8H4F3N | C8H7ClF3NO | |
| fluopyram_nC8H4F3N#pO | C8H7N1O2F3Cl1 | |
| fluopyram_nC8H5ClF3N | C8H6F3NO | |
| fluopyram_nC8H5ClF3N#pC3H5NO2S | C11H11N2O3F3S1 | |
| fluopyram_nC8H5ClF3N#pC5H7NO3S | | C13H13N2O4F3S1 |
| fluopyram_nC8H5ClF3N#pO | | C8H6N1O2F3 |
| fluopyram_nC8H5ClF3N#pS | | C8H6N1O1F3S1 |
| fluopyram_nC8H5ClF3N#pSCH2 | C9H8N1O1F3S1 | |
| fluopyram_nC8H5ClF3N#pSCH2O | C9H8N1O2F3S1 | |
| fluopyram_nC8H5ClF3N#pSCH2O2 | C9H8N1O3F3S1 | |
| fluopyram_nC8H6ClF3N2_pO | C8H5F3O2 | |
| fluopyram_nC8H6F3N_pO | C8H5ClF3NO2 | |
| fluopyram_nC8H6F3N_pO#pO | C8H5N1O3F3Cl1 | |
| fluopyram_nC9H8F3N_pO | C7H3ClF3NO2 | |
| fluopyram_pO | C16H9ClF6N2O2 | |
| fluopyram_pO#nH2 | C16H7N2O2F6Cl1 | |
| fluopyram_pO#pC3H5NO2S | | C19H14N3O4F6S1Cl1 |
| fluopyram_pO#pC5H7NO3S | C21H16N3O5F6S1Cl1 | |
| fluopyram_pO#pS | C16H9N2O2F6S1Cl1 | |
| fluopyram_pO#pSCH2 | C17H11N2O2F6S1Cl1 | |
| fluopyram_pO#pSCH2O | C17H11N2O3F6S1Cl1 | |
| fluopyram_pO#pSCH2O2 | C17H11N2O4F6S1Cl1 | |
| Fluopyram-benzamid | | C8H6F3NO |
| Fluopyram-PAA | | C8H5ClF3NO2 |
| Fluopyram-PCA | | C7H3ClF3NO2 |
| flupicolide_nCl_pH | C14H9Cl2F3N2O | |
| flupicolide_nCl_pH#pC2H2O5 | C16H11N2O6F3Cl2 | |
| flupicolide_nCl_pH#pC3H5NO2S | C17H14N3O3F3S1Cl2 | |
| flupicolide_nCl_pH#pC3H5NO3S | | C17H14N3O4F3S1Cl2 |
| flupicolide_nCl_pH#pC3H5NO4S | | C17H14N3O5F3S1Cl2 |
| flupicolide_nCl_pH#pC5H7NO3S | | C19H16N3O4F3S1Cl2 |
| flupicolide_nCl_pH#pC5H7NO4S | | C19H16N3O5F3S1Cl2 |
| flupicolide_nCl_pH#pC5H7NO5S | | C19H16N3O6F3S1Cl2 |
| flupicolide_nCl_pH#pO2 | | C14H9N2O3F3Cl2 |
| flupicolide_nCl_pH#pO3 | | C14H9N2O4F3Cl2 |
| flupicolide_nCl_pH#pO4 | | C14H9N2O5F3Cl2 |
| flupicolide_nCl_pH#pS | | C14H9N2O1F3S1Cl2 |
| flupicolide_nCl_pH#pSCH2 | C15H11N2O1F3S1Cl2 | |
| flupicolide_nCl_pH#pSCH2O | C15H11N2O2F3S1Cl2 | |
| flupicolide_nCl_pH#pSCH2O2 | C15H11N2O3F3S1Cl2 | |
| flupicolide_nCl_pH#pSCH2O3 | C15H11N2O4F3S1Cl2 | |
| flupicolide_nCl_pH#pSO | C14H9N2O2F3S1Cl2 | |
| flupicolide_nCl_pH#pSO2 | C14H9N2O3F3S1Cl2 | |
| flutolanil | C17H16F3NO2 | |
| flutolanil#pO | C17H16N1O3F3 | |
| flutolanil_C3H6 | C14H10F3NO2 | |
| flutolanil_C3H6#pCH2 | C15H12N1O2F3 | |
| flutolanil_C3H6#pCH2O | C15H12N1O3F3 | |
| flutolanil_C3H6#pO | | C14H10N1O3F3 |
| flutolanil_nH2_pO2 | C17H14F3NO4 | |
| flutriafol | C16H13F2N3O | |
| flutriafol#pCH2O | C17H15N3O2F2 | |
| flutriafol#pCH2O2 | C17H15N3O3F2 | |
| flutriafol#pH2O2 | C16H15N3O3F2 | |
| flutriafol#pO | | C16H13N3O2F2 |
| flutriafol#pO2 | | C16H13N3O3F2 |
| flutriafol_nC2HN3_pO | C14H12F2O2 | |
| folpet | C9H4Cl3NO2S | |
| folpet#pO | C9H4N1O3S1Cl3 | |
| folpet_DMS_acid | C4H10O6S4 | |
| folpet_DMS_acid#pO | C4H10O7S4 | |
| folpet_phtalic_acid | C8H6O4 | |
| folpet_phtalic_acid#nH2O | C8H4O3 | |
| folpet_PI | C8H5NO2 | |
| folpet_PI#pH2O | C8H7N1O3 | |
| folpet_TTC | C3H5NO2S2 | |
| fosthiazate | C9H18NO3PS2 | |
| fosthiazate#pH4 | C9H22N1O3P1S2 | |
| fosthiazate#pH4O | C9H22N1O4P1S2 | |
| fosthiazate_2-butane_sulfonic acid | C4H10O3S | |
| fosthiazate_nC3H3NS | C6H15O3PS | |
| fosthiazate_nC4H8S_pO | C5H10NO4PS | |
| fosthiazate_nC6H13O2PS | C3H5NOS | |
| haloxyfop | | C15H11ClF3NO4 |
| haloxyfop#nC3H4O2 | | C12H7N1O2F3Cl1 |
| haloxyfop#nC9H8O3 | | C6H3N1O1F3Cl1 |
| haloxyfop-ethoxyethyl | | C19H19ClF3NO5 |
| haloxyfop-ethoxyethyl#nC4H8O | | C15H11N1O4F3Cl1 |
| haloxyfop-methyl | | C16H13ClF3NO4 |
| haloxyfop-methyl#nCH2 | | C15H11N1O4F3Cl1 |
| Herbicides_2,4,5-T_ | | C8H5Cl3O3 |
| Herbicides_2,4-D_ | | C8H6Cl2O3 |
| Hydroxytebuconazole | | C16H22ClN3O2 |
| imazilil | C14H14Cl2N2O | |
| imazilil#nC3H4 | C11H10N2O1Cl2 | |
| imazilil#pH2O2 | C14H16N2O3Cl2 | |
| imazilil#pH2O4 | C14H16N2O5Cl2 | |
| imazilil#pO | C14H14N2O2Cl2 | |
| imazilil_metabolite3B | C16H17Cl2N3O4 | |
| imazilil_nC5H4_pO | | C9H10Cl2N2O2 |
| Imidacloprid | C9H10ClN5O2 | |
| imidacloprid | C9H10ClN5O2 | |
| Imidacloprid olefin | | C9H8ClN5O2 |
| imidacloprid#nH2 | C9H8N5O2Cl1 | |
| imidacloprid#nN2 | | C9H10N3O2Cl1 |
| imidacloprid#nN2O | C9H10N3O1Cl1 | |
| imidacloprid#nO | C9H10N5O1Cl1 | |
| imidacloprid#pO | C9H10N5O3Cl1 | |
| Imidacloprid, 5-hydroxy | | C9H10ClN5O3 |
| imidacloprid_nC3H5ClN4 | C6H5NO2 | |
| imidacloprid_nC3H5ClN4#pC3H5NO2S | | C9H10N2O4S1 |
| imidacloprid_nC3H5ClN4#pC5H7NO3S | C11H12N2O5S1 | |
| imidacloprid_nC3H5ClN4#pO2 | C6H5N1O4 | |
| imidacloprid_nC3H5ClN4#pS | | C6H5N1O2S1 |
| imidacloprid_nC3H5ClN4#pSCH2 | C7H7N1O2S1 | |
| imidacloprid_nC3H5ClN4#pSCH2O | C7H7N1O3S1 | |
| imidacloprid_nC3H5ClN4#pSCH2O2 | C7H7N1O4S1 | |
| imidacloprid_nC3H5ClN4_pC2H3NO | C8H8N2O3 | |
| imidacloprid_nC3H5ClN4_pC2H3NO#pC3H5NO2S | C11H13N3O5S1 | |
| imidacloprid_nC3H5ClN4_pC2H3NO#pC5H7NO3S | | C13H15N3O6S1 |
| imidacloprid_nC3H5ClN4_pC2H3NO#pS | | C8H8N2O3S1 |
| imidacloprid_nC3H5ClN4_pC2H3NO#pSCH2 | C9H10N2O3S1 | |
| imidacloprid_nC3H5ClN4_pC2H3NO#pSCH2O | C9H10N2O4S1 | |
| imidacloprid_nC3H5ClN4_pC2H3NO#pSCH2O2 | C9H10N2O5S1 | |
| imidacloprid_nC3H6N4 | C6H4ClNO2 | |
| imidacloprid_nC3H6N4#pC2H3NO | C8H7N2O3Cl1 | |
| imidacloprid_nC6H6ClN | C3H4N4O2 | |
| imidacloprid_nC6H6ClN#pH2 | C3H6N4O2 | |
| imidacloprid_nNO2_pH | C9H11ClN4 | |
| imidacloprid_nO2_pH2 | C9H12ClN5 | |
| imidacloprid_nO2_pH2#pC3O | C12H12N5O1Cl1 | |
| indoxacarb | C22H17ClF3N3O7 | |
| indoxacarb#nC | C21H17N3O7F3Cl1 | |
| indoxacarb#nC12H6ClNO4 | C10H11N2O3F3 | |
| indoxacarb#nC12H7ClO3 | C10H10N3O4F3 | |
| indoxacarb#nC13H9ClN2O4 | C9H8N1O3F3 | |
| indoxacarb#nC2H2O | C20H15N3O6F3Cl1 | |
| indoxacarb#nC2H2O2 | | C20H15N3O5F3Cl1 |
| indoxacarb#nC2H4O | | C20H13N3O6F3Cl1 |
| indoxacarb#nC3H2O2 | C19H15N3O5F3Cl1 | |
| indoxacarb#pO | C22H17N3O8F3Cl1 | |
| iprodione | C13H13Cl2N3O3 | |
| Iprodione metabolite M1 | | C9H6Cl2N2O2 |
| Iprodione metabolite M650F06 | | C10H7Cl2N3O3 |
| iprodione#nC4H7NO | | C9H6N2O2Cl2 |
| iprodione#pO | C13H13N3O4Cl2 | |
| iprodione#pO2 | C13H13N3O5Cl2 | |
| iprodione_nC3H6 | C10H7Cl2N3O3 | |
| iprodione_nC3H6#nC2O | C8H7N3O2Cl2 | |
| iprodione_pO_nC3H6 | C10H7Cl2N3O4 | |
| iprodione_pO_nC3H6#nC2O | C8H7N3O3Cl2 | |
| ivermectin B1a/avermectin H2B1a | C48H74O14 | |
| ivermectin B1a/avermectin H2B1a#nC5H10O2 | C43H64O12 | |
| ivermectin B1a/avermectin H2B1a#pC2H4O2 | C50H78O16 | |
| ivermectin B1a/avermectin H2B1a#pCH2O | | C49H76O15 |
| ivermectin B1a_nCH2 | C47H72O14 | |
| ivermectin B1a_nCH2#pCH2O2 | C48H74O16 | |
| ivermectin B1a_nCH2#pO | | C47H72O15 |
| ivermectin B1b/avermectin H2B1b | C47H72O14 | |
| ivermectin B1b/avermectin H2B1b#nC5H10O2 | C42H62O12 | |
| ivermectin B1b/avermectin H2B1b#pC2H4O2 | C49H76O16 | |
| ivermectin B1b/avermectin H2B1b#pCH2O | | C48H74O15 |
| ivermectin B1b_nCH2 | C46H70O14 | |
| ivermectin B1b_nCH2#pCH2O2 | C47H72O16 | |
| ivermectin B1b_nCH2#pO | | C46H70O15 |
| jasmolin I | C21H30O3 | |
| jasmolin I#pO | C21H30O4 | |
| jasmolin I#pO2 | C21H30O5 | |
| jasmolin I_nC10H14O | C11H16O2 | |
| jasmolin I_nC10H14O#pH2O2 | C11H18O4 | |
| jasmolin I_nC11H14O | C10H16O2 | |
| jasmolin I_nC11H14O#pH2O2 | C10H18O4 | |
| jasmolin I_nC11H14O#pO | | C10H16O3 |
| jasmolin I_nC11H16_pO | C10H14O4 | |
| jasmolin I_nH2_pO2 | C21H28O5 | |
| jasmolin I_nH2_pO2#pH2O2 | C21H30O7 | |
| jasmolin II | C22H30O5 | |
| jasmolin II#pH2O2 | C22H32O7 | |
| jasmolin II#pO | | C22H30O6 |
| jasmolin II#pO2 | C22H30O7 | |
| jasmolin II_nC11H14O | C11H16O4 | |
| jasmolin II_nC11H14O#nCH2 | C10H14O4 | |
| jasmolin II_nC11H14O3 | C11H16O2 | |
| jasmolin II_nC11H14O3#pH2O2 | C11H18O4 | |
| jasmolin II_nCH2 | C21H28O5 | |
| jasmolin II_nCH2#pH2O2 | C21H30O7 | |
| kresoxim-methyl | C18H19NO4 | |
| kresoxim-methyl_nC11H11NO3 | C7H8O | |
| kresoxim-methyl_nC11H11NO3#pO | C7H8O2 | |
| kresoxim-methyl_nC11H11NO3#pO2 | C7H8O3 | |
| kresoxim-methyl_nC11H13NO | C7H6O3 | |
| kresoxim-methyl_nC7H6 | C11H13NO4 | |
| kresoxim-methyl_nC7H6#nCH2 | C10H11N1O4 | |
| kresoxim-methyl_nC7H6#nH2O | C11H11N1O3 | |
| kresoxim-methyl_nC7H8_pO | C11H11NO5 | |
| kresoxim-methyl_nCH2 | C17H17NO4 | |
| kresoxim-methyl_nCH2#nCH2 | C16H15N1O4 | |
| kresoxim-methyl_nH2_pO2 | C18H17NO6 | |
| kresoxim-methyl_nH2_pO2#nC2H4 | C16H13N1O6 | |
| kresoxim-methyl_nH2_pO2#nCH2 | | C17H15N1O6 |
| kresoxim-methyl_pO | C18H19NO5 | |
| kresoxim-methyl_pO#nCH2 | C17H17N1O5 | |
| kresoxim-methyl_pO#nCH2H4 | C17H15N1O5 | |
| kresoxim-methyl_pO2 | C18H19NO6 | |
| kresoxim-methyl_pO2#nCH2H6 | C17H13N1O6 | |
| kresoxim-methyl_pO2#nCH4 | C17H15N1O6 | |
| kresoxim-methyl_pO3 | C18H19NO7 | |
| kresoxim-methyl_pO3#nCH2H6 | C17H13N1O7 | |
| kresoxim-methyl_pO3#nCH4 | C17H15N1O7 | |
| linuron | C9H10Cl2N2O2 | |
| linuron#nC2H4O | | C7H6N2O1Cl2 |
| linuron#nCH2 | C8H8N2O2Cl2 | |
| linuron#nCH2O | C8H8N2O1Cl2 | |
| linuron_pCH2O | C10H12Cl2N2O3 | |
| linuron_pO | C9H10Cl2N2O3 | |
| linuron_pO#nC2H4O | | C7H6N2O2Cl2 |
| linuron_pO#nCH2 | C8H8N2O3Cl2 | |
| linuron_pO#nCH2O | C8H8N2O2Cl2 | |
| lufenuron | C17H8Cl2F8N2O3 | |
| lufenuron#nC10H3Cl2F6NO2 | C7H5N1O1F2 | |
| lufenuron#nC10H4Cl2F6N2O | C7H4O2F2 | |
| lufenuron#nC7H2F2O | | C10H6N2O2F6Cl2 |
| lufenuron#nC8H3F2NO2 | C9H5N1O1F6Cl2 | |
| MCPA | C9H9ClO3 | |
| MCPA#pO | | C9H9O4Cl1 |
| mecoprop | | C10H11ClO3 |
| mecoprop#nH2pO2 | | C10H9O5Cl1 |
| mecoprop#pO | | C10H11O4Cl1 |
| mepanipyrim | C14H13N3 | |
| mepanipyrim#pH2O | C14H15N3O1 | |
| mepanipyrim#pH2O2 | C14H15N3O2 | |
| mepanipyrim#pH4O | | C14H17N3O1 |
| mepanipyrim#pH4O2 | C14H17N3O2 | |
| mepanipyrim#pH4O3 | C14H17N3O3 | |
| mepanipyrim#pO | | C14H13N3O1 |
| metamitron | | C10H10N4O |
| metamitron | | C10H10N4O |
| metamitron#pC3H5NO3S | | C13H15N5O4S1 |
| metamitron#pC5H7NO4S | | C15H17N5O5S1 |
| metamitron#pO | | C10H10N4O2 |
| metamitron#pSCH2O | | C11H12N4O2S1 |
| metamitron#pSCH2O2 | | C11H12N4O3S1 |
| metamitron#pSCH2O3 | | C11H12N4O4S1 |
| metamitron#pSO | | C10H10N4O2S1 |
| metamitron_nNH | | C10H9N3O |
| metamitron_nNH#pC3H5NO3S | | C13H14N4O4S1 |
| metamitron_nNH#pC5H7NO4S | | C15H16N4O5S1 |
| metamitron_nNH#pH2O | | C10H11N3O2 |
| metamitron_nNH#pO | | C10H9N3O2 |
| metamitron_nNH#pO2 | | C10H9N3O3 |
| metamitron_nNH#pSCH2O | | C11H11N3O2S1 |
| metamitron_nNH#pSCH2O2 | | C11H11N3O3S1 |
| metamitron_nNH#pSCH2O3 | | C11H11N3O4S1 |
| metamitron_nNH#pSO | | C10H9N3O2S1 |
| metazachlor | | C14H16ClN3O |
| metazachlor#pO | | C14H16N3O2Cl1 |
| metazachlor_nCl_pH | | C14H17N3O |
| metazachlor_nCl_pH#pC3H4O3S | | C17H21N3O4S1 |
| metazachlor_nCl_pH#pC3H5NO2S | | C17H22N4O3S1 |
| metazachlor_nCl_pH#pC5H7NO3S | | C19H24N4O4S1 |
| metazachlor_nCl_pH#pS | | C14H17N3O1S1 |
| metazachlor_nCl_pH#pSCH2 | | C15H19N3O1S1 |
| metazachlor_nCl_pH#pSCH2O | | C15H19N3O2S1 |
| metazachlor_nCl_pH#pSCH2O2 | | C15H19N3O3S1 |
| metazachlor_nCl_pOH | | C14H17N3O2 |
| metazachlor_nCl_pOH#pC3H5NO2S | | C17H22N4O4S1 |
| metazachlor_nCl_pOH#pC5H7NO3S | | C19H24N4O5S1 |
| metazachlor_nCl_pOH#pS | | C14H17N3O2S1 |
| metazachlor_nCl_pOH#pSCH2 | | C15H19N3O2S1 |
| metazachlor_nCl_pOH#pSCH2O | | C15H19N3O3S1 |
| metazachlor_nCl_pOH#pSCH2O2 | | C15H19N3O4S1 |
| metazachlor_nH2_pO2 | | C14H14ClN3O3 |
| metconazole | | C17H22ClN3O |
| metconazole#pO | | C17H22N3O2Cl1 |
| metconazole#pO2 | | C17H22N3O3Cl1 |
| metconazole#pO2nH2pO2 | | C17H20N3O5Cl1 |
| metconazole#pOnH2pO2 | | C17H20N3O4Cl1 |
| metham_ methyldithiocarbamic acid | | C2H5NS2 |
| metham_ methyldithiocarbamic acid#nCH5N | | C1S2 |
| metham_ methyldithiocarbamic acid#nCS2 | | C1H5N1 |
| metham_ methyldithiocarbamic acid#pCH2 | | C3H7N1S2 |
| metham_ methyldithiocarbamic acid#pOCH2 | | C3H7N1O1S2 |
| metham_ methyldithiocarbamic acid_nSpOCH2 | | C3H7NOS |
| metham_ methyldithiocarbamic acid_nSpOCH2#pC2H3NO2 | | C5H10N2O3S1 |
| metham_ methyldithiocarbamic acid_nSpOCH2#pC4H5NO3 | | C7H12N2O4S1 |
| metham_ methyldithiocarbamic acid_nSpOCH2#pO | | C3H7N1O2S1 |
| metham_ methyldithiocarbamic acid_nSpOCH2#pO2 | | C3H7N1O3S1 |
| Methyl_4-chloroindole-3-acetic acid | | C11H10ClNO2 |
| Methyl_4-chloroindole-3-acetic acid#nCH2 | | C10H8N1O2Cl1 |
| metolachlor | | C15H22ClNO2 |
| Metolachlor mercapturate | | C20H30N2O5S |
| metolachlor_nCH2 | C14H20ClNO2 | |
| metolachlor_nCHCl | C14H21NO2 | |
| metolachlor_nCHCl#pC3H5NO2S | C17H26N2O4S1 | |
| metolachlor_nCHCl#pC5H7NO3S | | C19H28N2O5S1 |
| metolachlor_nCHCl#pS | | C14H21N1O2S1 |
| metolachlor_nCHCl#pSCH2 | C15H23N1O2S1 | |
| metolachlor_nCHCl#pSCH2O | C15H23N1O3S1 | |
| metolachlor_nCHCl#pSCH2O2 | C15H23N1O4S1 | |
| metolachlor_nCHCl_nH2_pO2 | C14H19NO4 | |
| metolachlor_nCl_pH | C15H23NO2 | |
| metolachlor_nCl_pH#nC4H8 | C11H15N1O2 | |
| metolachlor_nCl_pH#pC3H5NO2S | C18H28N2O4S1 | |
| metolachlor_nCl_pH#pC5H7NO3S | | C20H30N2O5S1 |
| metolachlor_nCl_pH#pS | | C15H23N1O2S1 |
| metolachlor_nCl_pH#pSCH2 | C16H25N1O2S1 | |
| metolachlor_nCl_pH#pSCH2O | C16H25N1O3S1 | |
| metolachlor_nCl_pH#pSCH2O2 | C16H25N1O4S1 | |
| metrafenon | C19H21BrO5 | |
| metrafenon#nC2H4 | C17H17O5Br1 | |
| metrafenon#nCH2 | | C18H19O5Br1 |
| metrafenon#pO | | C19H21O6Br1 |
| metrafenon_nBr_pH | C19H20O6 | |
| metrafenon_nH2_pO | C19H19BrO6 | |
| milbemectin_ethyl | C32H46O7 | |
| milbemectin_ethyl#pO | C32H46O8 | |
| milbemectin_ethyl#pO2 | C32H46O9 | |
| milbemectin_methyl | C31H44O7 | |
| milbemectin_methyl#pO | C31H44O8 | |
| milbemectin_methyl#pO2 | C31H44O9 | |
| milneb | C12H22N4S4 | |
| milneb#nC11H22N4S2 | C1S2 | |
| myclobutanil | C15H17ClN4 | |
| myclobutanil#pO | C15H17N4O1Cl1 | |
| myclobutanil#pO2 | C15H17N4O2Cl1 | |
| myclobutanil_nH2_pO2 | C15H15ClN4O2 | |
| myclobutanil_nH2_pO2#nO | C15H15N4O1Cl1 | |
| myclobutanil_nNH_pO3 | C15H16ClN3O3 | |
| nabam/amobam/maneb/mancozeb/zineb/mancopper/metiram/tecoram_ethylenebis(dithiocarbamic acid) | C4H8N2S4 | |
| nabam/amobam/maneb/mancozeb/zineb/mancopper/metiram/tecoram_ethylenebis(dithiocarbamic acid)#nH2S | C4H6N2S3 | |
| nabam/amobam/maneb/mancozeb/zineb/mancopper/metiram/tecoram_ethylenebis(dithiocarbamic acid)#nCH2S3 | C3H6N2S1 | |
| nabam/amobam/maneb/mancozeb/zineb/mancopper/metiram/tecoram_ethylenebis(dithiocarbamic acid)#nC3H8N2S2 | C1S2 | |
| nabam/amobam/maneb/mancozeb/zineb/mancopper/metiram/tecoram_ethylenebis(dithiocarbamic acid)_ETU | C3H6N2S | |
| nabam/amobam/maneb/mancozeb/zineb/mancopper/metiram/tecoram_ethylenebis(dithiocarbamic acid)_ETU#pO | C3H6N2O1S1 | |
| nabam/amobam/maneb/mancozeb/zineb/mancopper/metiram/tecoram_ethylenebis(dithiocarbamic acid)_ETU#pCH2 | C4H8N2S1 | |
| nabam/amobam/maneb/mancozeb/zineb/mancopper/metiram/tecoram_ethylenebis(dithiocarbamic acid)_ETU_nH2S_pO2 | C3H4N2O2 | |
| nabam/amobam/maneb/mancozeb/zineb/mancopper/metiram/tecoram_ethylenebis(dithiocarbamic acid)_ETU_nS_pO | C3H6N2O | |
| Orgophosphorous_met_DEAMPY | | C8H15N3O |
| Orgophosphorous_met_IMPY | | C7H12N2O |
| Orgophosphorous_met_MDA | | C6H11PO6S2 |
| Orgophosphorous_met_PNP | | C6H5NO3 |
| Orgophosphorous_met_TCPY | | C5H2Cl3NO |
| ortho-phenylphenol | C12H10O | |
| ortho-phenylphenol#pO | C12H10O2 | |
| ortho-phenylphenol_quinone | C12H8O2 | |
| oxamyl | C7H13N3O3S | |
| oxamyl#nC2H3NO | C5H10N2O2S1 | |
| oxamyl#nC3H7NO2S | C4H6N2O1 | |
| oxamyl#nC4H9NO2S | C3H4N2O1 | |
| oxamyl_dimethylaxamic_acid | C4H7NO3 | |
| oxamyl_dimethylaxamic_acid#nCH2 | C3H5N1O3 | |
| oxamyl_nCH2 | C6H11N3O3S | |
| oxamyl_nCH2#nC2H3NO | C4H8N2O2S1 | |
| oxyfluorfen | C15H11ClF3NO4 | |
| oxyfluorfen#nC8H7NO3 | | C7H4O1F3Cl1 |
| oxyfluorfen#nCH2 | C14H9N1O4F3Cl1 | |
| oxyfluorfen#nCH4 | C14H7N1O4F3Cl1 | |
| oxyfluorfen#nNO2nH2pOHnCF3pCO2H | C15H11O5Cl1 | |
| oxyfluorfen#nNO2pNCH2O | C16H13N1O3F3Cl1 | |
| oxyfluorfen#nNO2pNCH2OnC2H4 | | C14H9N1O3F3Cl1 |
| oxyfluorfen#nNO2pNH2 | C15H13N1O2F3Cl1 | |
| oxyfluorfen#nNO2pNH2nC2H4 | | C13H9N1O2F3Cl1 |
| oxyfluorfen#nNO2pNH4C2OnC2H4 | C15H11N1O3F3Cl1 | |
| oxyfluorfen#nNO2pOH | | C15H12O3F3Cl1 |
| oxyfluorfen#nNO2pOHnCF3pCO2H | C15H13O5Cl1 | |
| paraclox | C8H6ClNO3 | |
| penconazole | C13H15Cl2N3 | |
| penconazole#nC11H12Cl2 | C2H3N3 | |
| penconazole#pO | C13H15N3O1Cl2 | |
| penconazole#pO2 | C13H15N3O2Cl2 | |
| penconazole_nH2_pO2 | C13H13Cl2N3O2 | |
| penconazole_nH2_pO2#nC2H4 | C11H9N3O2Cl2 | |
| penconazole_nH2_pO2#nCH2 | | C12H11N3O2Cl2 |
| penconazole_nH2_pO3 | C13H13Cl2N3O3 | |
| penconazole_nH2_pO3#nCH2 | C12H11N3O3Cl2 | |
| penconazole_nH2_pO3#pO | C13H13N3O4Cl2 | |
| pencycuron | C19H21ClN2O | |
| pencycuron#nC10H13NpO2 | | C9H8N1O3Cl1 |
| pencycuron#nC5H8 | C14H13N2O1Cl1 | |
| pencycuron#nC5H8pO | C14H13N2O2Cl1 | |
| pencycuron#nC7H5NO | C12H16N1Cl1 | |
| pencycuron#pCH2O2 | | C20H23N2O3Cl1 |
| pencycuron#pO | | C19H21N2O2Cl1 |
| pencycuron#pO2 | C19H21N2O3Cl1 | |
| pendimethalin | C13H19N3O4 | |
| pendimethalin#pC2H2 | | C15H21N3O4 |
| pendimethalin#pC2H2O | | C15H21N3O5 |
| pendimethalin#pO | C13H19N3O5 | |
| pendimethalin#pO2 | C13H19N3O6 | |
| pendimethalin_nH2_pO2 | C13H17N3O6 | |
| pendimethalin_nH2_pO2#pC2H2O | C15H19N3O7 | |
| pendimethalin_nH2_pO2#pC2H2O2 | C15H19N3O8 | |
| pendimethalin_nH2_pO2#pO | C13H17N3O7 | |
| pentachlorophenol | | C6H1Cl5O |
| permethrin | C21H20Cl2O3 | |
| permethrin#pO | C21H20O4Cl2 | |
| permethrin_nC13H10O | C8H10Cl2O2 | |
| permethrin_nC13H10O#pO | C8H10O3Cl2 | |
| permethrin_nC8H10Cl2 | C13H10O3 | |
| permethrin_nC8H10Cl2#pC2H3NO | C15H13N1O4 | |
| permethrin_nC8H10Cl2#pC2H5NO2S | C15H15N1O5S1 | |
| permethrin_nC8H10Cl2#pO | | C13H10O4 |
| permethrin_nC8H8Cl2O | C13H12O2 | |
| permethrin_nC8H8Cl2O#pO | C13H12O3 | |
| phenothrin | C23H26O3 | |
| phenothrin#pO | C23H26O4 | |
| phenothrin_nC10H14O | C13H12O2 | |
| phenothrin_nC10H14O#pO | C13H12O3 | |
| phenothrin_nC10H16 | C13H10O3 | |
| phenothrin_nC10H16#pC2H3NO | C15H13N1O4 | |
| phenothrin_nC10H16#pC2H5NO2S | C15H15N1O5S1 | |
| phenothrin_nC10H16#pO | | C13H10O4 |
| phenothrin_nC13H10O | C10H16O2 | |
| phenothrin_nC13H10O#pO | C10H16O3 | |
| phosphamidon | | C10H19ClNO5P |
| phosphamidon#nC2H4 | | C8H15N1O5P1Cl1 |
| phosphamidon#nC2H5O3P | | C8H14N1O2Cl1 |
| phosphamidon#nC2H5O3PnC2H4 | | C6H10N1O2Cl1 |
| phosphamidon#nCH2 | | C9H17N1O5P1Cl1 |
| piperonylbutoxide | C19H30O5 | |
| piperonylbutoxide_nC | C18H30O6 | |
| piperonylbutoxide_nC#nC2H4 | C16H26O6 | |
| piperonylbutoxide_nC#nC4H8O | C14H22O5 | |
| piperonylbutoxide_nC#nC6H12O2 | C12H18O4 | |
| piperonylbutoxide_nC#nC8H16O3 | C10H14O3 | |
| piperonylbutoxide_nH2_pO2 | C19H28O7 | |
| piperonylbutoxide_nH2_pO2#nC2H4 | C17H24O7 | |
| piperonylbutoxide_nH2_pO2#nC4H8O | C15H20O6 | |
| piperonylbutoxide_nH2_pO2#nC6H12O2 | C13H16O5 | |
| piperonylbutoxide_nH2_pO2#nC8H16O3 | C11H12O4 | |
| piperonylbutoxide_pO | C19H30O6 | |
| piperonylbutoxide_pO#nC2H4 | C17H26O6 | |
| piperonylbutoxide_pO#nC4H8O | C15H22O5 | |
| piperonylbutoxide_pO#nC6H12O2 | C13H18O4 | |
| piperonylbutoxide_pO#nC8H16O3 | C11H14O3 | |
| pirimicarb | C11H18N4O2 | |
| pirimicarb#pO | C11H18N4O3 | |
| pirimicarb_nC2H4 | C9H14N4O2 | |
| pirimicarb_nC2H4#nC3H5NO | C6H9N3O1 | |
| pirimicarb_nC3H5NO | C8H13N3O | |
| pirimicarb_nC3H5NO#pC3H5NO3S | C11H18N4O4S1 | |
| pirimicarb_nC3H5NO#pC5H7NO4S | | C13H20N4O5S1 |
| pirimicarb_nC3H5NO#pO | | C8H13N3O2 |
| pirimicarb_nC3H5NO#pSCH2O | C9H15N3O2S1 | |
| pirimicarb_nC3H5NO#pSCH2O2 | C9H15N3O3S1 | |
| pirimicarb_nC3H5NO#pSCH2O3 | C9H15N3O4S1 | |
| pirimicarb_nC3H5NO#pSO | | C8H13N3O2S1 |
| pirimicarb_nC4H7NO | C7H11N3O | |
| pirimicarb_nC4H7NO#pC3H5NO3S | C10H16N4O4S1 | |
| pirimicarb_nC4H7NO#pC5H7NO4S | | C12H18N4O5S1 |
| pirimicarb_nC4H7NO#pO | | C7H11N3O2 |
| pirimicarb_nC4H7NO#pSCH2O | C8H13N3O2S1 | |
| pirimicarb_nC4H7NO#pSCH2O2 | C8H13N3O3S1 | |
| pirimicarb_nC4H7NO#pSCH2O3 | C8H13N3O4S1 | |
| pirimicarb_nC4H7NO#pSO | | C7H11N3O2S1 |
| pirimicarb_nC5H9NO | C6H9N3O | |
| pirimicarb_nC5H9NO#pC3H5NO3S | C9H14N4O4S1 | |
| pirimicarb_nC5H9NO#pC5H7NO4S | C11H16N4O5S1 | |
| pirimicarb_nC5H9NO#pO | C6H9N3O2 | |
| pirimicarb_nC5H9NO#pSCH2O | C7H11N3O2S1 | |
| pirimicarb_nC5H9NO#pSCH2O2 | C7H11N3O3S1 | |
| pirimicarb_nC5H9NO#pSCH2O3 | C7H11N3O4S1 | |
| pirimicarb_nC5H9NO#pSO | | C6H9N3O2S1 |
| pirimicarb_nCH2 | C10H16N4O2 | |
| pirimicarb_nCH2#nC3H5NO | C7H11N3O1 | |
| Pirimiphos-methyl | | C11H20N3O3PS |
| pirimiphos-methyl | C11H20N3O3PS | |
| pirimiphos-methyl#nC2H4 | C9H16N3O3P1S1 | |
| pirimiphos-methyl#nC4H8 | C7H12N3O3P1S1 | |
| pirimiphos-methyl#pO | | C11H20N3O4P1S1 |
| pirimiphos-methyl_nC2H5PO2S | C9H15N3O | |
| pirimiphos-methyl_nC2H5PO2S#nC2H4 | C7H11N3O1 | |
| pirimiphos-methyl_nC2H5PO2S#nC4H8 | C5H7N3O1 | |
| pirimiphos-methyl_nC2H5PO2S#pO | | C9H15N3O2 |
| pirimiphos-methyl_nC9H13N3 | C2H7O3PS | |
| pirimiphos-methyl_nC9H13N3#nCH2 | C1H5O3P1S1 | |
| pirimiphos-methyl_nC9H13N4S_pO | C2H7O4P | |
| pirimiphos-methyl_nC9H13N4S_pO#nCH2 | C1H5O4P1 | |
| pirimiphos-methyl_nCH2 | C10H18N3O3PS | |
| pirimiphos-methyl_nCH2#nC2H4 | C8H14N3O3P1S1 | |
| pirimiphos-methyl_nCH2#nC4H8 | C6H10N3O3P1S1 | |
| pirimiphos-methyl_nCH2#pO | | C10H18N3O4P1S1 |
| pirimiphos-methyl_nSpO | C11H20N3O4P | |
| pirimiphos-methyl_nSpO#nC2H4 | C9H16N3O4P1 | |
| pirimiphos-methyl_nSpO#nC4H8 | C7H12N3O4P1 | |
| pirimiphos-methyl_nSpO#pO | | C11H20N3O5P1 |
| Pirimiphos-methyl-N-desethyl | | C9H16N3O3PS |
| prallethrin | C19H24O3 | |
| prallethrin#pO | C19H24O4 | |
| prallethrin_nC10H14O | C9H10O2 | |
| prallethrin_nC10H14O#pC3H5NO2S | C12H15N1O4S1 | |
| prallethrin_nC10H14O#pC5H7NO3S | | C14H17N1O5S1 |
| prallethrin_nC10H14O#pH2O | | C9H12O3 |
| prallethrin_nC10H14O#pO | | C9H10O3 |
| prallethrin_nC10H14O#pS | | C9H10O2S1 |
| prallethrin_nC10H14O#pSCH2 | C10H12O2S1 | |
| prallethrin_nC10H14O#pSCH2O | C10H12O3S1 | |
| prallethrin_nC10H14O#pSCH2O2 | C10H12O4S1 | |
| prallethrin_nC9H8O | C10H16O2 | |
| prallethrin_nC9H8O#pO | C10H16O3 | |
| prochloraz | C15H16Cl3N3O2 | |
| Prochloraz metabolite BTS 44595 | | C12H15Cl3N2O2 |
| Prochloraz metabolite BTS 44596 | | C13H15Cl3N2O3 |
| prochloraz_nC2HN_pO | C13H15Cl3N2O3 | |
| prochloraz_nC2HN_pO#nC | C12H15N2O3Cl3 | |
| prochloraz_nC2HN_pO#nC5H10N2 | C8H5O3Cl3 | |
| prochloraz_nC2HN_pO#nC5H8N2 | | C8H7O3Cl3 |
| prochloraz_nC2HN_pO#nCO | | C12H15N2O2Cl3 |
| prochloraz_nC6H7N | C9H9Cl3N2O2 | |
| prochloraz_nC6H7N#nC3H6N2O | C6H3O1Cl3 | |
| prochloraz_nC6H7N#nCH2N2 | | C8H7O2Cl3 |
| procymidone | | C13H11Cl2NO2 |
| procymidone#nH2pO2 | | C13H9N1O4Cl2 |
| procymidone#pH2O | | C13H13N1O3Cl2 |
| procymidone#pH2OnH2pO2 | | C13H11N1O5Cl2 |
| procymidone#pH2OpO | | C13H13N1O4Cl2 |
| procymidone#pO | | C13H11N1O3Cl2 |
| Propamocarb | | C9H20N2O2 |
| propamocarb | C9H20N2O2 | |
| propamocarb#nCH2 | C8H18N2O2 | |
| propamocarb_nH2_pO | C9H18N2O3 | |
| propamocarb_nH2_pO#nCH2 | C8H16N2O3 | |
| propamocarb_nH2_pO2 | C9H18N2O4 | |
| propamocarb_nH2_pO2#nCH2 | C8H16N2O4 | |
| propamocarb_pO | C9H20N2O3 | |
| propamocarb_pO#nCH2 | C8H18N2O3 | |
| propamocarb_pO#pO | | C9H20N2O4 |
| propiconazole | C15H17Cl2N3O2 | |
| propiconazole#nC13H14Cl2O2 | C2H3N3 | |
| propiconazole#pO | | C15H17N3O3Cl2 |
| propiconazole_nC5H10O | C10H7Cl2N3O | |
| propiconazole_nC5H10O#nO | | C10H7N3Cl2 |
| propiconazole_nC5H10O#pH2 | C10H9N3O1Cl2 | |
| propiconazole_nC5H10O#pH2O | C10H9N3O2Cl2 | |
| propiconazole_nC5H9Cl | C10H8ClN3O2 | |
| propiconazole_nC5H9Cl#nO | | C10H8N3O1Cl1 |
| propiconazole_nC5H9Cl#pH2 | C10H10N3O2Cl1 | |
| propiconazole_nC5H9Cl#pH2O | C10H10N3O3Cl1 | |
| propiconazole_nH2_pO2 | C15H15Cl2N3O4 | |
| propiconazole_nH2_pO2#nC2H4 | C13H11N3O4Cl2 | |
| propiconazole_nH2_pO2#nCH2 | | C14H13N3O4Cl2 |
| propiconazole_nH2_pO3 | C15H15Cl2N3O5 | |
| propiconazole_nH2_pO3#nC2H4 | C13H11N3O5Cl2 | |
| propiconazole_nH2_pO3#nCH2 | | C14H13N3O5Cl2 |
| propineb_propylenebis(dithiocarbamate) | C5H10N2S4 | |
| propineb_propylenebis(dithiocarbamate)#nC4H10N2S2 | C1S2 | |
| propineb_propylenebis(dithiocarbamate)#nCH2S3 | | C4H8N2S1 |
| propineb_propylenebis(dithiocarbamate)#nH2S | | C5H8N2S3 |
| propineb_propylenebis(dithiocarbamate)_PTU | C4H8N2S | |
| propineb_propylenebis(dithiocarbamate)_PTU_nH2SpO | C4H6N2O2 | |
| propineb_propylenebis(dithiocarbamate)_PTU_nSpO | C4H8N2O | |
| propyzamide | C12H11Cl2NO | |
| propyzamide#nC5H6 | | C7H5N1O1Cl2 |
| propyzamide#nC5H6O3 | | C7H5N1Cl2 |
| propyzamide#nC5H7NpO | | C7H4O2Cl2 |
| propyzamide#nC5H7NpOnH2OpC2H5NO2 | | C9H7N1O3Cl2 |
| propyzamide#pH2 | | C12H13N1O1Cl2 |
| propyzamide#pH2O | C12H13N1O2Cl2 | |
| propyzamide#pH2O2 | C12H13N1O3Cl2 | |
| propyzamide#pH2O3 | C12H13N1O4Cl2 | |
| propyzamide#pH4O | C12H15N1O2Cl2 | |
| propyzamide#pH4O2 | C12H15N1O3Cl2 | |
| propyzamide#pHnCl | C12H12N1O1Cl1 | |
| prothioconazole | C14H15Cl2N3OS | |
| prothioconazole#nC12H12Cl2OS | | C2H3N3 |
| prothioconazole#pCH2 | C15H17N3O1S1Cl2 | |
| prothioconazole#pO | C14H15N3O2S1Cl2 | |
| prothioconazole_nS | C14H15Cl2N3O | |
| prothioconazole_nS#pCH2O2 | | C15H17N3O3Cl2 |
| prothioconazole_nS#pH2O2 | | C14H17N3O3Cl2 |
| prothioconazole_nS#pO | C14H15N3O2Cl2 | |
| prothioconazole_nS#pO2 | C14H15N3O3Cl2 | |
| prothioconazole_nS_pO | C14H15Cl2N3O2 | |
| Prothioconazole-desthio | | C14H15Cl2N3O |
| pymetrozine | C10H11N5O | |
| pymetrozine#pO | C10H11N5O2 | |
| pymetrozine_ nC4H4N4O2 | C6H5NO | |
| pymetrozine_ nC4H4N4O2#pCH3NO | C7H8N2O2 | |
| pymetrozine_ nC4H4N4O2#pNH | | C6H6N2O1 |
| pymetrozine_ nC4H4N4O2#pO | | C6H5N1O2 |
| pymetrozine_nC6H3N | C4H8N4O | |
| pymetrozine_nC6H3N#nNH | C4H7N3O1 | |
| pymetrozine_nC6H5N_pO | C4H6N4O2 | |
| pymetrozine_nC6H5N_pO#nNH | C4H5N3O2 | |
| pymetrozine_nC6H5N_pO#pC2H2O | C6H8N4O3 | |
| pymetrozine_nH2_pO2 | C10H9N5O3 | |
| Pyraclostrobin | C19H18ClN3O4 | |
| Pyraclostrobin#pC3H5NO2S | | C22H23N4O6S1Cl1 |
| Pyraclostrobin#pC5H7NO3S | | C24H25N4O7S1Cl1 |
| Pyraclostrobin#pO | C19H18N3O5Cl1 | |
| Pyraclostrobin#pO2 | C19H18N3O6Cl1 | |
| Pyraclostrobin#pS | C19H18N3O4S1Cl1 | |
| Pyraclostrobin#pSCH2 | C20H20N3O4S1Cl1 | |
| Pyraclostrobin#pSCH2O | C20H20N3O5S1Cl1 | |
| Pyraclostrobin#pSCH2O2 | C20H20N3O6S1Cl1 | |
| Pyraclostrobin_nC10H11NO3 | C9H7ClN2O | |
| Pyraclostrobin_nC10H11NO3#pO | C9H7N2O2Cl1 | |
| Pyraclostrobin_nC9H5ClN2 | C10H13NO4 | |
| Pyraclostrobin_nC9H5ClN2#nCH2 | | C9H11N1O4 |
| Pyraclostrobin_nC9H5ClN2#nCH2O | C9H11N1O3 | |
| Pyraclostrobin_nC9H7ClN2_pO | C10H11NO5 | |
| Pyraclostrobin_nC9H7ClN2_pO#nCH2O | C9H9N1O4 | |
| Pyraclostrobin_nCH2O | C18H16ClN3O3 | |
| Pyraclostrobin_nCH2O#pO | C18H16N3O4Cl1 | |
| Pyraclostrobin_nCH2O#pO2 | C18H16N3O5Cl1 | |
| pyrethrin I | C21H28O3 | |
| pyrethrin I#pO | C21H28O4 | |
| pyrethrin I#pO2 | C21H28O5 | |
| pyrethrin I_nC10H14O | C11H14O2 | |
| pyrethrin I_nC10H14O#pH2O2 | C11H16O4 | |
| pyrethrin I_nC11H12O | C10H16O2 | |
| pyrethrin I_nC11H12O#pH2O2 | C10H18O4 | |
| pyrethrin I_nC11H12O#pO | | C10H16O3 |
| pyrethrin I_nC11H14_pO | C10H14O4 | |
| pyrethrin I_nH2_pO2 | C21H26O5 | |
| pyrethrin I_nH2_pO2#pH2O2 | C21H28O7 | |
| pyrethrin II | C22H28O5 | |
| pyrethrin II#pH2O2 | C22H30O7 | |
| pyrethrin II#pO | | C22H28O6 |
| pyrethrin II#pO2 | C22H28O7 | |
| pyrethrin II_nC11H12O | C11H16O4 | |
| pyrethrin II_nC11H12O#nCH2 | C10H14O4 | |
| pyrethrin II_nC11H14O3 | C11H14O2 | |
| pyrethrin II_nC11H14O3#pH2O2 | C11H16O4 | |
| pyrethrin II_nCH2 | C21H26O5 | |
| pyrethrin II_nCH2#pH2O2 | C21H28O7 | |
| Pyrethroid_met_3-PBA | | C13H10O3 |
| Pyrethroid_met_4-F-3-PBA | | C13H9FO3 |
| Pyrethroid_met_cis-DBCA | | C8H10Br2O2 |
| Pyrethroid_met_cis-DCCA | | C8H10Cl2O2 |
| Pyrethroid_met_trans-DCCA | | C8H10Cl2O2 |
| pyrichlor | | C5H2Cl3NO |
| pyrimethanil | C12H13N3 | |
| Pyrimethanil metabolite M605F002/M605F003 | | C12H13N3O |
| Pyrimethanil metabolite M605F003/M605F002 | | C12H13N3O |
| pyrimethanil#pO | C12H13N3O1 | |
| pyrimethanil#pO2 | C12H13N3O2 | |
| pyriproxyfen | C20H19NO3 | |
| pyriproxyfen#pO | C20H19N1O4 | |
| pyriproxyfen#pO2 | C20H19N1O5 | |
| pyriproxyfen_nC12H10 | | C8H9NO3 |
| pyriproxyfen_nC12H8O | | C8H11NO2 |
| pyriproxyfen_nC5H3N | C15H16O3 | |
| pyriproxyfen_nC5H3N#pO | C15H16O4 | |
| pyriproxyfen_nC6H4 | | C14H15NO3 |
| pyriproxyfen_nC8H9NO | C12H10O2 | |
| pyriproxyfen_nC8H9NO#pO | C12H10O3 | |
| quizalofop | C17H13ClN2O4 | |
| quizalofop#nC3H4O2 | C14H9N2O2Cl1 | |
| quizalofop#nC9H8O3 | C8H5N2O1Cl1 | |
| quizalofop-ethyl | | C19H17ClN2O4 |
| quizalofop-ethyl#nC2H4 | | C17H13N2O4Cl1 |
| quizalofop-methyl | | C18H15ClN2O4 |
| quizalofop-methyl#nCH2 | | C17H13N2O4Cl1 |
| quizalofop-tefuryl | | C22H21ClN2O5 |
| quizalofop-tefuryl#nC5H8O | | C17H13N2O4Cl1 |
| resmethrin | C22H26O3 | |
| resmethrin#pO | C22H26O4 | |
| resmethrin_nC10H14O | C12H12O2 | |
| resmethrin_nC10H14O#pO | C12H12O3 | |
| resmethrin_nC10H16 | C12H10O3 | |
| resmethrin_nC10H16#pC2H3NO | C14H13N1O4 | |
| resmethrin_nC10H16#pC2H5NO2S | C14H15N1O5S1 | |
| resmethrin_nC10H16#pO | | C12H10O4 |
| resmethrin_nC12H10O | C10H16O2 | |
| resmethrin_nC12H10O#pO | C10H16O3 | |
| spinosyn A | C41H65NO10 | |
| spinosyn A#nC2H4 | C39H61N1O10 | |
| spinosyn A#nC8H15NO | | C33H50O9 |
| spinosyn A#nCH2 | | C40H63N1O10 |
| spinosyn A#pO | C41H65N1O11 | |
| spinosyn A_nCH2 | C40H63NO10 | |
| spinosyn A_nCH2#nC8H15NO | C32H48O9 | |
| spinosyn A_nCH2#pO | | C40H63N1O11 |
| spinosyn D | C42H67NO10 | |
| spinosyn D#nC2H4 | C40H63N1O10 | |
| spinosyn D#nC8H15NO | | C34H52O9 |
| spinosyn D#nCH2 | | C41H65N1O10 |
| spinosyn D#pO | C42H67N1O11 | |
| spinosyn D_nCH2 | C41H65NO10 | |
| spinosyn D_nCH2#nC8H15NO | C33H50O9 | |
| spinosyn D_nCH2#pO | | C41H65N1O11 |
| spirodiclofen | C21H24Cl2O4 | |
| spirodiclofen_nC13H18O | C8H6Cl2O3 | |
| spirodiclofen_nC13H18O#nCH2O | C7H4O2Cl2 | |
| spirodiclofen_nC13H18O#nH2 | | C8H4O3Cl2 |
| spirodiclofen_nC6H10 | C15H14Cl2O4 | |
| spirodiclofen_nC6H10#nH2 | C15H12O4Cl2 | |
| spirodiclofen_nC6H10#nH2O | C15H12O3Cl2 | |
| spirodiclofen_nC6H10#pH2O | C15H16O5Cl2 | |
| spirodiclofen_nC6H10O | C15H14Cl2O3 | |
| spirotetramat | C21H27NO5 | |
| Spirotetramat Metabolite BYI08330-cis-enol | | C18H23NO3 |
| spirotetramat_nC3H4O2 | C18H23NO3 | |
| spirotetramat_nC3H4O2#nCH2 | C17H21N1O3 | |
| spirotetramat_nC3H4O2#pO | | C18H23N1O4 |
| spirotetramat_nC4H6O | C17H21NO4 | |
| spirotetramat_nC4H6O#pH2O | C17H23N1O5 | |
| spirotetramat_nC4H6O#pO | | C17H21N1O5 |
| spirotetramat_nC4H6O#pO2 | C17H21N1O6 | |
| spirotetramat_nC4H6O#pO3 | C17H21N1O7 | |
| spirotetramat_nC4H8O | C17H19NO4 | |
| spirotetramat_nC4H8O#pO | C17H19N1O5 | |
| spirotetramat_nC4H8O#pO2 | C17H19N1O6 | |
| spirotetramat_nC4H8O#pO3 | C17H19N1O7 | |
| spiroxamine | C18H35NO2 | |
| spiroxamine#nC2H4 | C16H31N1O2 | |
| spiroxamine#nC3H6 | C15H29N1O2 | |
| spiroxamine_nC10H16 | C8H19NO2 | |
| spiroxamine_nC8H17NO | C10H18O | |
| spiroxamine_nC8H17NO#pO | C10H18O2 | |
| spiroxamine_nH2pO2 | C18H33NO4 | |
| spiroxamine_nH2pO2#nC2H4 | C16H29N1O4 | |
| spiroxamine_nH2pO2#nC3H6 | C15H27N1O4 | |
| spiroxamine_nH2pO2#pO | C18H33N1O5 | |
| spiroxamine_pO | C18H35NO3 | |
| spiroxamine_pO#nC2H4 | C16H31N1O3 | |
| spiroxamine_pO#nC3H6 | C15H29N1O3 | |
| sulcotrione | C14H13ClO5S | |
| sulcotrione#nC6H6O | C8H7O4S1Cl1 | |
| sulcotrione#nH2 | C14H11O5S1Cl1 | |
| sulcotrione#pH2O | C14H15O6S1Cl1 | |
| sulcotrione#pO | C14H13O6S1Cl1 | |
| sulcotrione_nCl_pH | C14H14O5S | |
| sulcotrione_nCl_pH#nC6H6O | C8H8O4S1 | |
| sulcotrione_nCl_pH#pH2O | | C14H16O6S1 |
| tebuconazole | C16H22ClN3O | |
| tebuconazole#pO | C16H22N3O2Cl1 | |
| tebuconazole#pO2 | C16H22N3O3Cl1 | |
| tebuconazole_nC14H19ClO | C2H3N3 | |
| tebuconazole_nC2H3N3 | C14H21ClO2 | |
| tebuconazole_nH2_pO2 | C16H20ClN3O3 | |
| tebuconazole_nH2_pO2#nCO2 | C15H20N3O1Cl1 | |
| tebuconazole_nH2_pO2#pO | | C16H20N3O4Cl1 |
| tebufenpyrad | C18H24ClN3O | |
| tebufenpyrad#nCH2 | C17H22N3O1Cl1 | |
| tebufenpyrad_nC11H14 | C7H10ClN3O | |
| tebufenpyrad_nC11H14#pO | C7H10N3O2Cl1 | |
| tebufenpyrad_nC11H16_pO | C7H8ClN3O2 | |
| tebufenpyrad_nC12H17N_pO | C6H7ClN2O2 | |
| tebufenpyrad_nH2_pO2 | C18H22ClN3O3 | |
| tebufenpyrad_nH2_pO2#pO | C18H22N3O4Cl1 | |
| tebufenpyrad_nH4_pO3 | C18H20ClN3O4 | |
| tebufenpyrad_nH4_pO3#pO | C18H20N3O5Cl1 | |
| tebufenpyrad_pO | C18H24ClN3O2 | |
| tebufenpyrad_pO#nCH2 | C17H22N3O2Cl1 | |
| tebufenpyrad_pO#nH2 | C18H22N3O2Cl1 | |
| tebufenpyrad_pO#pO | | C18H24N3O3Cl1 |
| tecoram | C10H18N4S8 | |
| tecoram#nC3H5NS2 | C7H13N3S6 | |
| tecoram#nC6H10N2S4 | C4H8N2S4 | |
| terbuthylazine | C9H16ClN5 | |
| terbuthylazine#pO | C9H16N5O1Cl1 | |
| terbuthylazine_nC2H4 | C7H12ClN5 | |
| terbuthylazine_nC2H4#pO | C7H12N5O1Cl1 | |
| terbuthylazine_nC2H4Cl_pH | C7H13N5 | |
| terbuthylazine_nC2H4Cl_pH#pC3H5NO2S | C10H18N6O2S1 | |
| terbuthylazine_nC2H4Cl_pH#pC5H7NO3S | | C12H20N6O3S1 |
| terbuthylazine_nC2H4Cl_pH#pS | | C7H13N5S1 |
| terbuthylazine_nC2H4Cl_pH#pSCH2 | C8H15N5S1 | |
| terbuthylazine_nC2H4Cl_pH#pSCH2O | C8H15N5O1S1 | |
| terbuthylazine_nC2H4Cl_pH#pSCH2O2 | C8H15N5O2S1 | |
| terbuthylazine_nC2H4Cl_pHO | C7H13N5O | |
| terbuthylazine_nC2H4Cl_pHO#pC3H5NO2S | C10H18N6O3S1 | |
| terbuthylazine_nC2H4Cl_pHO#pC5H7NO3S | | C12H20N6O4S1 |
| terbuthylazine_nC2H4Cl_pHO#pO | | C7H13N5O2 |
| terbuthylazine_nC2H4Cl_pHO#pS | | C7H13N5O1S1 |
| terbuthylazine_nC2H4Cl_pHO#pSCH2 | C8H15N5O1S1 | |
| terbuthylazine_nC2H4Cl_pHO#pSCH2O | C8H15N5O2S1 | |
| terbuthylazine_nC2H4Cl_pHO#pSCH2O2 | C8H15N5O3S1 | |
| terbuthylazine_nC2H5Cl_pO3 | C7H11N5O3 | |
| terbuthylazine_nC2H6_pO2 | C7H10ClN5O2 | |
| terbuthylazine_nH2_pO2 | C9H14ClN5O2 | |
| tertraconazole_nC11H8Cl2F4O | C2H3N3 | |
| tetrachlorophenol | | C6H2Cl4O |
| tetraconazole | C13H11Cl2F4N3O | |
| tetraconazole#pO | C13H11N3O2F4Cl2 | |
| tetraconazole_nC2F4 | C11H11Cl2N3O | |
| tetraconazole_nC2F4_nC2HN3 | C9H10Cl2O | |
| tetraconazole_nC2F4_nC2HN3#pC3H5NO2S | C12H15N1O3S1Cl2 | |
| tetraconazole_nC2F4_nC2HN3#pC5H7NO3S | | C14H17N1O4S1Cl2 |
| tetraconazole_nC2F4_nC2HN3#pS | | C9H10O1S1Cl2 |
| tetraconazole_nC2F4_nC2HN3#pSCH2 | C10H12O1S1Cl2 | |
| tetraconazole_nC2F4_nC2HN3#pSCH2O | C10H12O2S1Cl2 | |
| tetraconazole_nC2F4_nC2HN3#pSCH2O2 | C10H12O3S1Cl2 | |
| tetraconazole_nC2F4H2_pO | C11H9Cl2N3O2 | |
| tetraconazole_nC2F4H2_pO_nC2HN3 | C9H8Cl2O2 | |
| tetraconazole_nC2F4H2_pO_nC2HN3#pC3H5NO2S | C12H13N1O4S1Cl2 | |
| tetraconazole_nC2F4H2_pO_nC2HN3#pC5H7NO3S | | C14H15N1O5S1Cl2 |
| tetraconazole_nC2F4H2_pO_nC2HN3#pS | | C9H8O2S1Cl2 |
| tetraconazole_nC2F4H2_pO_nC2HN3#pSCH2 | C10H10O2S1Cl2 | |
| tetraconazole_nC2F4H2_pO_nC2HN3#pSCH2O | C10H10O3S1Cl2 | |
| tetraconazole_nC2F4H2_pO_nC2HN3#pSCH2O2 | C10H10O4S1Cl2 | |
| tetraconazole_nF2_pO2 | C13H11Cl2F2N3O3 | |
| tetramethrin | C19H25NO4 | |
| tetramethrin#pO | C19H25N1O5 | |
| tetramethrin_nC10H14O | C9H11NO3 | |
| tetramethrin_nC10H14O#nCH2O | C8H9N1O2 | |
| tetramethrin_nC9H9NO2 | C10H16O2 | |
| tetramethrin_nC9H9NO2#pO | C10H16O3 | |
| tetramethrin_TCDA | C8H12O4 | |
| tetramethrin_TCDA#pO | C8H12O5 | |
| tetramethrin_TPI | C8H9NO2 | |
| tetramethrin_TPI#pH2 | C8H11N1O2 | |
| tetramethrin_TPI#pH2O | C8H11N1O3 | |
| tetramethrin_TPI#pH2SO3 | C8H11N1O5S1 | |
| tetramethrin_TPI#pO | | C8H9N1O3 |
| thiabendazole | C10H7N3S | |
| thiabendazole#pO | C10H7N3O1S1 | |
| thiabendazole_nCNS_pOH | C9H8N2O | |
| Thiacloprid | | C10H9ClN4S |
| thiacloprid | C10H9ClN4S | |
| thiacloprid#pH2O | C10H11N4O1S1Cl1 | |
| thiacloprid_nC2H2S | C8H7ClN4 | |
| thiacloprid_nC2H2S#pC3H5NO2S | C11H12N5O2S1Cl1 | |
| thiacloprid_nC2H2S#pC5H7NO3S | | C13H14N5O3S1Cl1 |
| thiacloprid_nC2H2S#pNH | | C8H8N5Cl1 |
| thiacloprid_nC2H2S#pS | | C8H7N4S1Cl1 |
| thiacloprid_nC2H2S#pSCH2 | C9H9N4S1Cl1 | |
| thiacloprid_nC2H2S#pSCH2O | C9H9N4O1S1Cl1 | |
| thiacloprid_nC2H2S#pSCH2O2 | C9H9N4O2S1Cl1 | |
| thiacloprid_nCClN_pH2O4 | C9H11N3O4S | |
| thiacloprid_pC2H4O5_nN2 | C12H13ClN2O5S | |
| thiacloprid_pC2H4O5_nN2#nC3H2O2 | C9H11N2O3S1Cl1 | |
| thiacloprid_pC2H4O5_nN2#nC4H2O3 | C8H11N2O2S1Cl1 | |
| thiacloprid_pC2H4O5_nN2#nC4H4O3 | | C8H9N2O2S1Cl1 |
| thiacloprid_pC2H4O5_nN2#nC4H6ClNO | | C8H7N1O4S1 |
| thiacloprid_pC2H4O5_nN2#nC4H6O2 | C8H7N2O3S1Cl1 | |
| thiacloprid_pC2H4O5_nN2#nC6H9NO3 | C6H4N1O2S1Cl1 | |
| thiacloprid_pC3H8O4_nN2 | C13H17ClN2O4S | |
| thiacloprid_pC3H8O4_nN2#nC4H7ClO | C9H10N2O3S1 | |
| thiacloprid_pCH6O2 | C11H15ClN4O2S | |
| thiacloprid_pCH6O2#nH2 | | C11H13N4O2S1Cl1 |
| thiacloprid_pCH6O2#nH2O | C11H13N4O1S1Cl1 | |
| thiacloprid_pCH6O3 | C11H15ClN4O3S | |
| thiacloprid_pCH6O3#nC3H6O2 | C8H9N4O1S1Cl1 | |
| thiacloprid_pCH6O3#nC5H7NO2S | C6H8N3O1Cl1 | |
| thiacloprid_pCH6O3#nC6H8ClNO | | C5H7N3O2S1 |
| thiacloprid_pCH6O3#nC7H10ClNO2 | C4H5N3O1S1 | |
| thiacloprid_pCH6O3#nC7H12ClNO2 | C4H3N3O1S1 | |
| thiacloprid_pCH6O3#nC7H12ClNO3 | C4H3N3S1 | |
| thiacloprid_pCH6O3#nC7H8ClN | | C4H7N3O3S1 |
| thiacloprid_pCH6O3#nH4O | | C11H11N4O2S1Cl1 |
| thiacloprid_pO | C10H9ClN4OS | |
| thiacloprid_pO#nH2 | C10H7N4O1S1Cl1 | |
| thiacloprid_pO_nS | C10H9ClN4O | |
| thiacloprid_pO_nS#pH4O2 | C10H13N4O3Cl1 | |
| thiamethoxam | C8H10ClN5O3S | |
| thiamethoxam#nC2H2O | C6H8N5O2S1Cl1 | |
| thiamethoxam#nC3H4O | C5H6N5O2S1Cl1 | |
| thiamethoxam#nC3H4O | C5H6N5O2S1Cl1 | |
| thiamethoxam#nC3H5N | | C5H5N4O3S1Cl1 |
| thiamethoxam#nN2O | C8H10N3O2S1Cl1 | |
| thiamethoxam_C4H6N4O3 | C4H4ClNS | |
| thiamethoxam_C4H6N4O3#pC3H5NO2S | | C7H9N2O2S2Cl1 |
| thiamethoxam_C4H6N4O3#pC5H7NO3S | C9H11N2O3S2Cl1 | |
| thiamethoxam_C4H6N4O3#pC5H7NO4S | C9H11N2O4S2Cl1 | |
| thiamethoxam_C4H6N4O3#pS | | C4H4N1S2Cl1 |
| thiamethoxam_C4H6N4O3#pSCH2 | C5H6N1S2Cl1 | |
| thiamethoxam_C4H6N4O3#pSCH2O | C5H6N1O1S2Cl1 | |
| thiamethoxam_C4H6N4O3#pSCH2O2 | C5H6N1O2S2Cl1 | |
| thiamethoxam_nC2HClO | C8H11N5O3S | |
| thiamethoxam_nC2HClO#pC3H5NO2S | C11H16N6O5S2 | |
| thiamethoxam_nC2HClO#pC5H7NO3S | | C13H18N6O6S2 |
| thiamethoxam_nC2HClO#pS | | C8H11N5O3S2 |
| thiamethoxam_nC2HClO#pSCH2 | C9H13N5O3S2 | |
| thiamethoxam_nC2HClO#pSCH2O | C9H13N5O4S2 | |
| thiamethoxam_nC2HClO#pSCH2O2 | C9H13N5O5S2 | |
| thiamethoxam_nC3H5ClN4O_pS | C5H5NO2S2 | |
| thiamethoxam_nC4H8N4O | C4H2ClNO2S | |
| thiamethoxam_nClN2O_pH | C8H11N3O2S | |
| thiamethoxam_nClN2O_pH#pC3H5NO2S | C11H16N4O4S2 | |
| thiamethoxam_nClN2O_pH#pC5H7NO3S | | C13H18N4O5S2 |
| thiamethoxam_nClN2O_pH#pS | | C8H11N3O2S2 |
| thiamethoxam_nClN2O_pH#pSCH2 | C9H13N3O2S2 | |
| thiamethoxam_nClN2O_pH#pSCH2O | C9H13N3O3S2 | |
| thiamethoxam_nClN2O_pH#pSCH2O2 | C9H13N3O4S2 | |
| thiamethoxam_nNO2_pH | C8H11ClN4OS | |
| thiamethoxam_nNO2_pH#nC2H2O | C6H9N4S1Cl1 | |
| thiamethoxam_nNO2_pH#nC2H3N | C6H8N3O1S1Cl1 | |
| thiamethoxam_nNO2_pH#nC3H4O | | C5H7N4S1Cl1 |
| thiamethoxam_nNO2_pH#nC3H5N | C5H6N3O1S1Cl1 | |
| thiamethoxam_nNO2_pH#nC4H6N2O | | C4H5N2S1Cl1 |
| thiamethoxam_pC3H4 | C11H14ClN5O3S | |
| thiamethoxam_pC3H4#nC2H2O | C9H12N5O2S1Cl1 | |
| thiamethoxam_pC3H4#nC4H4O2 | C7H10N5O1S1Cl1 | |
| Thiametoxam metabolite CGA 355190 | | C8H10ClN3O2S |
| thiophanate-methyl | C12H14N4O4S2 | |
| thiophanate-methyl#pO | C12H14N4O5S2 | |
| thiophanate-methyl_nC3H5NO2S2 | C9H9N3O2 | |
| thiophanate-methyl_nC3H5NO2S2#nC2H2O2 | C7H7N3 | |
| thiophanate-methyl_nC3H5NO2S2#pO | | C9H9N3O3 |
| thiophanate-methyl_nS_pO | C12H14N4O5S | |
| thiophanate-methyl_nS_pO#pO | C12H14N4O6S1 | |
| thiophanate-methyl_nS2_pO2 | C12H14N4O6 | |
| thiophanate-methyl_nS2_pO2#pO | C12H14N4O7 | |
| thiram | C6H12N2S4 | |
| thiram#nC3H5NS2 | C3H7N1S2 | |
| thiram#nC4H5NS4 | C2H7N1 | |
| thiram#nC5H12N2S2 | C1S2 | |
| tolclofos-methyl | C9H11Cl2O3PS | |
| tolclofos-methyl#nC2H5O2PS | C7H6O1Cl2 | |
| tolclofos-methyl#nC2H5O2PSpO | C7H6O2Cl2 | |
| tolclofos-methyl#nCH2 | | C8H9O3P1S1Cl2 |
| tolclofos-methyl#nSpO | | C9H11O4P1Cl2 |
| transfluthrin | C15H12Cl2F4O2 | |
| transfluthrin#pO | C15H12O3F4Cl2 | |
| transfluthrin_FB-Ac | C7H2F4O2 | |
| transfluthrin_FB-Ac#pC2H3NO | C9H5N1O3F4 | |
| transfluthrin_FB-Ac#pC2H5NO2S | C9H7N1O4F4S1 | |
| transfluthrin_nC7H2F4 | C8H10Cl2O2 | |
| transfluthrin_nC7H2F4#pO | C8H10O3Cl2 | |
| transfluthrin_nC8H8Cl2O | C7H4F4O | |
| triadimenol | C14H18ClN3O2 | |
| triadimenol#nC8H13N3O | C6H5O1Cl1 | |
| triadimenol#nCH4 | C13H14N3O2Cl1 | |
| triadimenol#pO | | C14H18N3O3Cl1 |
| triadimenol_nH2 (triadimefon) | C14H16ClN3O2 | |
| triadimenol_nH2 (triadimefon)#nO | C14H16N3O1Cl1 | |
| triadimenol_nH2 (triadimefon)_nC_pO2 | C13H16ClN3O4 | |
| triadimenol_nH2 (triadimefon)_nC_pO2#pO | C13H16N3O5Cl1 | |
| triadimenol_nH2 (triadimefon)_pO | C14H16ClN3O3 | |
| triadimenol_nH2 (triadimefon)_pO#nC | | C13H16N3O3Cl1 |
| triadimenol_nH2 (triadimefon)_pO#nC4H8 | C10H8N3O3Cl1 | |
| triadimenol_nH2_pO2 | C14H16ClN3O4 | |
| trichlorophenol | | C6H3Cl3O |
| triclopyr | | C7H4Cl3NO3 |
| trifloxystrobin | C20H19F3N2O4 | |
| Trifloxystrobin metabolite CGA 321113 | C19H17F3N2O4 | |
| trifloxystrobin#nC2H4 | C18H15N2O4F3 | |
| trifloxystrobin#nC3H4O2 | C17H15N2O2F3 | |
| trifloxystrobin#nC3H5NO2 | C17H14N1O2F3 | |
| trifloxystrobin#nCH2 | | C19H17N2O4F3 |
| trifloxystrobin_nC10H10F3N_pO | C10H9NO5 | |
| trifloxystrobin_nC10H8F3N | C10H11NO4 | |
| trifloxystrobin_nC10H8F3N#nC2H5N | C8H6O4 | |
| trifloxystrobin_nC10H8F3N#nC2H5NO2 | C8H6O2 | |
| trifloxystrobin_nC10H8F3N#nCH2 | C9H9N1O4 | |
| trifloxystrobin_nC11H11NO3 | C9H8F3NO | |
| trifloxystrobin_nC11H11NO3#pO | C9H8N1O2F3 | |
| trifloxystrobin_nC11H12N2O | C9H7F3O3 | |
| trifloxystrobin_nC11H12N2O#nCH2O | C8H5O2F3 | |
| trifloxystrobin_pO | C20H19F3N2O5 | |
| trifloxystrobin_pO#nC2H4 | C18H15N2O5F3 | |
| trifloxystrobin_pO#nC2H5N | C18H14N1O5F3 | |
| trifloxystrobin_pO#nC3H5NO | C17H14N1O4F3 | |
| trifloxystrobin_pO#nC3H6O | | C17H13N2O4F3 |
| trifloxystrobin_pO#nC3H7N | C17H12N1O5F3 | |
| trifloxystrobin_pO#nCH2 | | C19H17N2O5F3 |
| trifloxystrobin_pO2 | C20H19F3N2O6 | |
| trifloxystrobin_pO2#nCH2 | C19H17N2O6F3 | |
| trifloxystrobin_pO2#nCH4 | C19H15N2O6F3 | |
| triticonazole | C17H20ClN3O | |
| triticonazole#nH2pO2 | C17H18N3O3Cl1 | |
| triticonazole#nH2pO3 | C17H18N3O4Cl1 | |
| triticonazole#pO | | C17H20N3O2Cl1 |
| triticonazole#pO2 | C17H20N3O3Cl1 | |
